# Frequency-oriented hierarchical fusion network for single image raindrop removal

**DOI:** 10.1371/journal.pone.0301439

**Published:** 2024-05-23

**Authors:** Juncheng Wang, Jie Zhang, Shuai Guo, Bo Li

**Affiliations:** 1 Qingdao University of Technology, School of Humanities and Foreign Languages, Qingdao, China; 2 Qingdao University of Technology, School of Science, Qingdao, China; 3 Chinese Academy of Forestry, Research Institute of Forestry Policy and Information, Beijing, China; Purdue University, UNITED STATES

## Abstract

Single image raindrop removal aims at recovering high-resolution images from degraded ones. However, existing methods primarily employ pixel-level supervision between image pairs to learn spatial features, thus ignoring the more discriminative frequency information. This drawback results in the loss of high-frequency structures and the generation of diverse artifacts in the restored image. To ameliorate this deficiency, we propose a novel frequency-oriented Hierarchical Fusion Network (HFNet) for raindrop image restoration. Specifically, to compensate for spatial representation deficiencies, we design a dynamic adaptive frequency loss (DAFL), which allows the model to adaptively handle the high-frequency components that are difficult to recover. To handle spatially diverse raindrops, we propose a hierarchical fusion network to efficiently learn both contextual information and spatial features. Meanwhile, a calibrated attention mechanism is proposed to facilitate the transfer of valuable information. Comparative experiments with existing methods indicate the advantages of the proposed algorithm.

## Introduction

Image restoration in severe weather has always been a research topic in low-level vision tasks [1–4]. When the imaging device captures a variety of distracting factors such as rain, snow, and fog in bad weather may cause the missing of structure and degrade the visual quality. In recent years, there has been an increasing interest in the study of single-image deraining, and significant advances have been made [5, 6]. However, single image raindrop removal is extremely challenging due to the diversity and uncertainty of raindrop morphology.

Previous studies of single-image raindrop removal are mainly summarized into two trends: physical model-based and deep learning-based [7–13]. Traditional physical model-based approaches rely on prior information to establish mathematical models and decompose the raindrop image into raindrop maps and clean background images by sparse coding and dictionary learning [14–16]. Although the model-based approach can produce expected results, it can only extract the shallow features of raindrop images [17, 18]. Since the deep semantic information can not be well represented, such methods will result in artifacts or color distortion in the restored image. Conversely, the deep learning-driven solutions have yielded more promising outcomes because of its ability to represent image content and semantic information through non-linear mapping. These methods accomplish restoration from rain image to rain-free image by introducing constraints related to the physical properties of raindrops. Specifically, Qian et al. [19] introduced the recurrent attention mechanism to locate the raindrop regions and used adversarial training to accomplish raindrop image restoration. Based on the positive and negative effects of raindrops on the background, Shao et al. [20] proposed the uncertainty mask to enhance the representation accuracy of raindrops. In addition, some progressive restoration models are meticulously tailored to facilitate the reconstruction of textures and structures.

**Fig 1:**
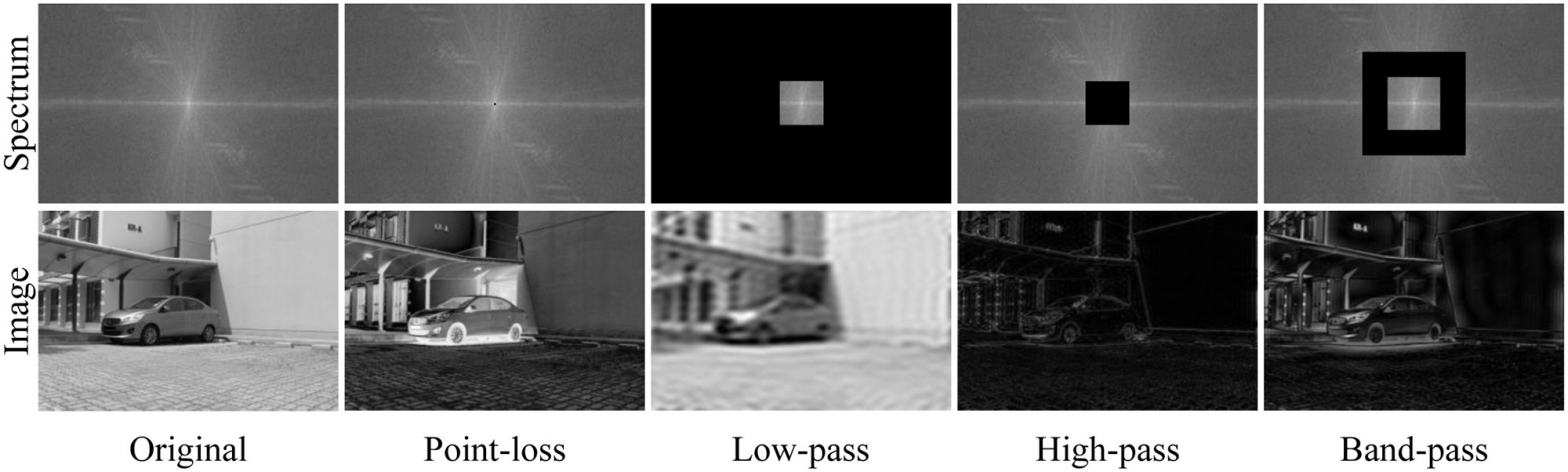


However, previous raindrop removal efforts [19–23] usually narrow the gap between the real image and the restored image in the spatial-domain. Despite achieving satisfactory performance in some scenarios, the restored images still suffer from distortion. This is because the frequency-domain distance between images is also an important prior knowledge that they do not take into account. The frequency domain representation contains two advantages: (1) Global properties. According to the mathematical formulation of the Fourier transform [24], each frequency in the Fourier domain is the result of the summation of all pixels in the spatial domain. Therefore, the frequency domain representation has global perceptual capability. (2) Discriminative features. In contrast to representing an image in the spatial domain, different frequencies can be clearly separated in the Fourier domain. As shown in Fig 1, when the frequency of a point or different regions are missing in the spectrum, the entire generated image is altered and corresponds to the presence of different artifacts. Therefore, reconstructing these missing frequencies can preserve the structural integrity of the background. Motivated by the advantage of frequency-domain representation, we we intend to exploit the frequency distance constraint to compensate for the spatial representation deficiencies. Our DAFL facilitates the recovery of clear images with more details.

Based on the aforementioned analysis, we propose a novel frequency-aware hierarchical fusion network (HFNet) for removing raindrops. To be specific, we propose a dynamic adaptive frequency loss (DAFL) to narrow the gap between the restored image and the real image in the frequency domain. This objective function allows the network to focus on hard frequencies with dynamic weights, and preserve a more complete image structure by applying frequency distance constraints at different flexible scales. Then, we design a hierarchical fusion network which boosts the restoration quality by fusing enriched features from different stages. The proposed framework consists of three stages, each of which first employ a recurrent processing module (RPM) to extract reliable raindrop location information. During the first two stages, an efficient UNet is adopted to obtain multi-scale complementary features. Subsequently, we devise the calibrated attention module (MCAB) to guide the delivery of valuable information. At the final stage, we design a cascade resolution module (CRM) to restore high-resolution images by exploring deep texture details.

The primary contributions are listed below:

- We propose a dynamic adaptive frequency loss (DAFL) to complement the shortcomings of spatial constraints, which effectively focuses on the learning of high-frequency information that is hard to generate.

- We design hierarchical feature fusion networks to learn enriched contextual information and spatial features. Meanwhile, a calibrated attention mechanism is proposed to facilitate the transfer of useful information in different stages.

- Experiments validate that our method has significant advantages over existing methods and is more robust to spatially diverse raindrop degradation.

## Related work

### Rain removal

The study of raindrop image restoration is gradually attracting the attention of researchers due to its challenge and significance. Initially, researchers explored the work of video rain removal based on time domain and sparse property analysis [25–27]. Compared with video rain removal that can achieve rain removal from the spatio-temporal correlation between frames [28, 29], single-image rain removal is more challenging. This is because it obtains less prior information, especially for raindrop removal with more complex situations. The current research on single-image raindrop removal tasks is mainly divided into traditional model-driven methods [14, 16, 30, 31] and deep learning-based data-driven methods [19–22, 32, 33].

The single-image rain removal work was first developed using a traditional model-based approach. These methods rely more on the properties of the rain map and the background scene to construct and optimize the constraint function. For example, Kang et al. [30] introduce a morphological component analysis method to deal with raindrop removal by applying a bilateral filter. To further enhance rain removal efficiency, Gu et al. [31] propose a model that involves both convolutional properties and synthetic sparse representation to effectively extract the image texture layer and flexibly model different image structure types. Nevertheless, these methods are more laborious in processing images with heavy raindrop density or similar semantic information between the raindrop map and the background image.

Deep learning is currently making dramatic advances in low-level vision tasks, including single image raindrop removal. Eigen et al. [32] trained a convolutional neural network to map raindrop images to clean ones. However, this approach is not suitable for handling complex raindrop scenarios because of the simple network structure. Later, Qian et al. [19] propose an attention generative adversarial network (AttGAN), which introduces the recurrent attention module into the GAN to highlight the raindrop region. To better utilize the interaction between different blocks, Liu et al. [33] design a dual residual connection network (DuRN) in a modular fashion to facilitate reconstructing the structure and texture of an image. Shao et al. [20] explore the effect of raindrop uncertainty on the background and design a multi-scale attentional network to recover more complete details by utilizing multi-scale features. Unfortunately, these methods primarily explore the spatial features and neglect the more discriminative frequency information, resulting in artifacts on the recovered image.

### Multi-stage hierarchical learning

In contrast to single-stage deraining methods, multi-stage learning achieves better results by increasing the depth of the network and focusing on different features at each stage [34–37]. However, we note that the feature extraction operations of upsampling and downsampling result in the missing of essential features in the image. Simultaneously, the increased complexity of the network makes the contribution of each module unclear. To ameliorate these limitation, Ren et al. [21] devide a progressive recurrent network, which provides a better and simpler deraining network. Along this direction, Jiang et al. [22] analyze the complementary properties of rain patterns at different scales and design a multi-scale fusion network to promote the detail rendering in recovered images.

Different from existing raindrop removal models in [19–22, 32, 33], we propose a frequency-level constraints to ensure that the restored image and the clear image are as consistent as possible in the frequency domain. Besides, the designed hierarchical feature fusion networks makes full use of the multi-stage feature, dramatically reducing the consumption of valuable information by incorporating intermediate attributes from various periods. Meanwhile, The experiment in Section IV shows the superiority of our method.

## Proposed method

**Fig 2:**
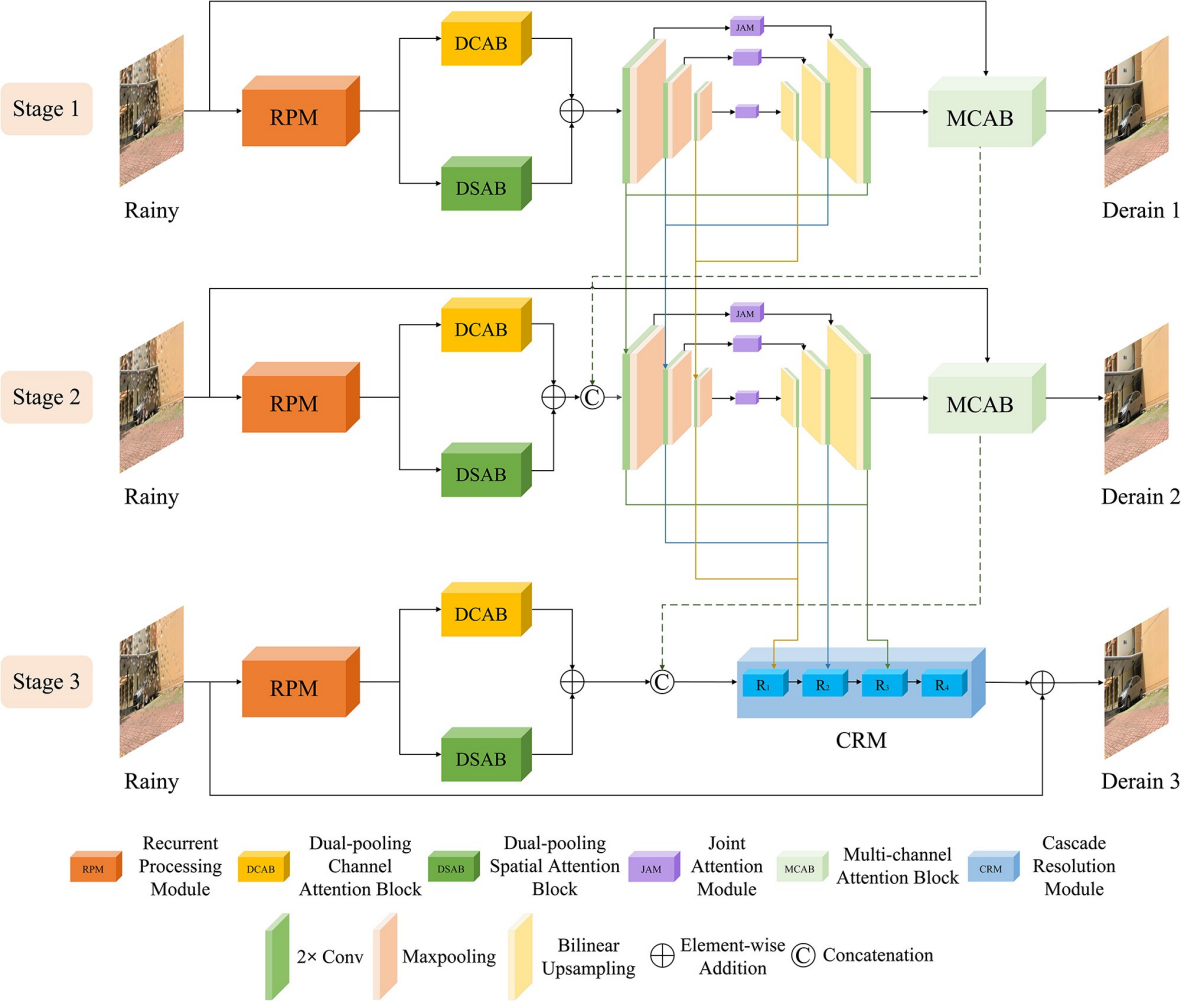


In this section, we propose a frequency-guided hierarchical fusion network (HFNet) to remove raindrops by utilizing frequency representation and cross-stage progressive feature fusion. The overview of the designed HFNet is displayed in Fig 2. We will detail the implementation of the individual modules in the next part.

### Hierarchical fusion network

It is depicted in Fig 2 that our HFNet takes the form of a three-stage. In each stage, we extract profitable multi-scale features and accurate spatial details through the cooperation of several modules. Also, a variety of attention modules is introduced to highlight the critical region.

#### Recurrent processing module

**Fig 3:**
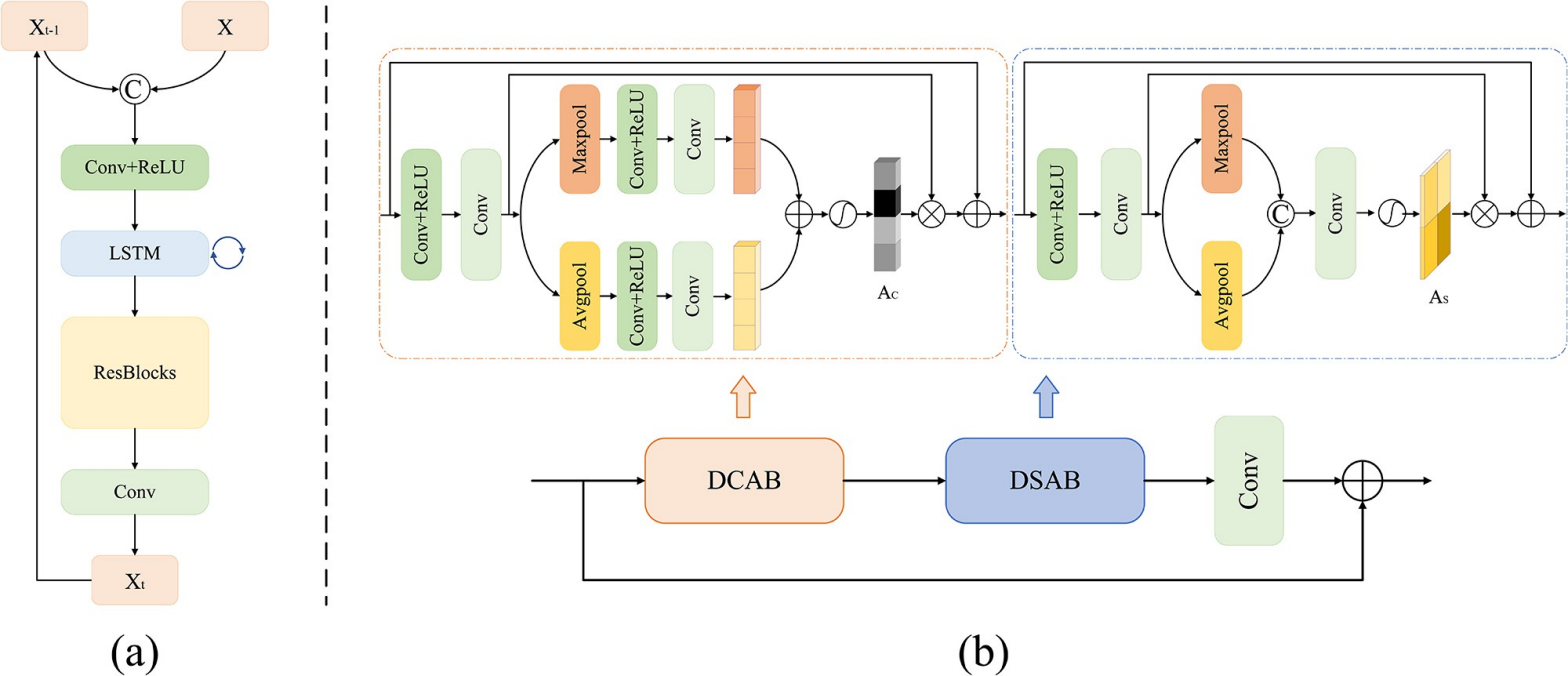


Fig 3(a) shows the structure of the RPM module. It is mainly composed of convolution layer, Long Short-Term Memory (LSTM) unit, and ResBlocks [38, 39]. The input of LSTM comes from both the output of the front convolution layer and the LSTM in the previous iteration. Meanwhile, we adopt five residual blocks in ResBlocks, each containing two convolution layers and a ReLU activation function. RPM can gradually extract the deep representation of raindrop images through the recurrent iteration. Each iteration uses the original raindrop image and the output of RPM in the previous iteration as the input, which can be expressed as:

$$\begin{array}{c} X_{t}=f_{RPM}\left(X_{t-1},X\right), \end{array}$$ (1)

where *f*_*RPM*_ denotes the RPM recurrent operation. Also, *X*, *X*_*t*−1_ and *X*_*t*_ mean the original image, output features of the previous stage, and output features of the current stage, respectively.

#### Encoder-decoder with joint attention block

Different from the previous ones [40, 41], the adopted encoder-decoder incorporates a JAM module at the skip connection. JAM includes a dual-pooling channel attention block (DCAB) and a dual-pooling spatial attention block (DSAB) in series, as well as a residual structure, as shown in Fig 3(b). This strategy can not only pass the original feature at different scales to the decoder, but also amplify the essential features and suppress the effect of potentially disturbing features. Here our encoder adopts double convolution and max-pooling to complete multiple downsampling operations. While the decoder adopts bilinear upsampling operation and double convolution to double the size of the feature map and halve the number of channels, respectively. As depicted in Fig 2, we also design cross-stage fusion connections between the encoder-decoder of the first two stages to help enrich the network with raindrop features.

#### Multi-channel attention module

**Fig 4:**
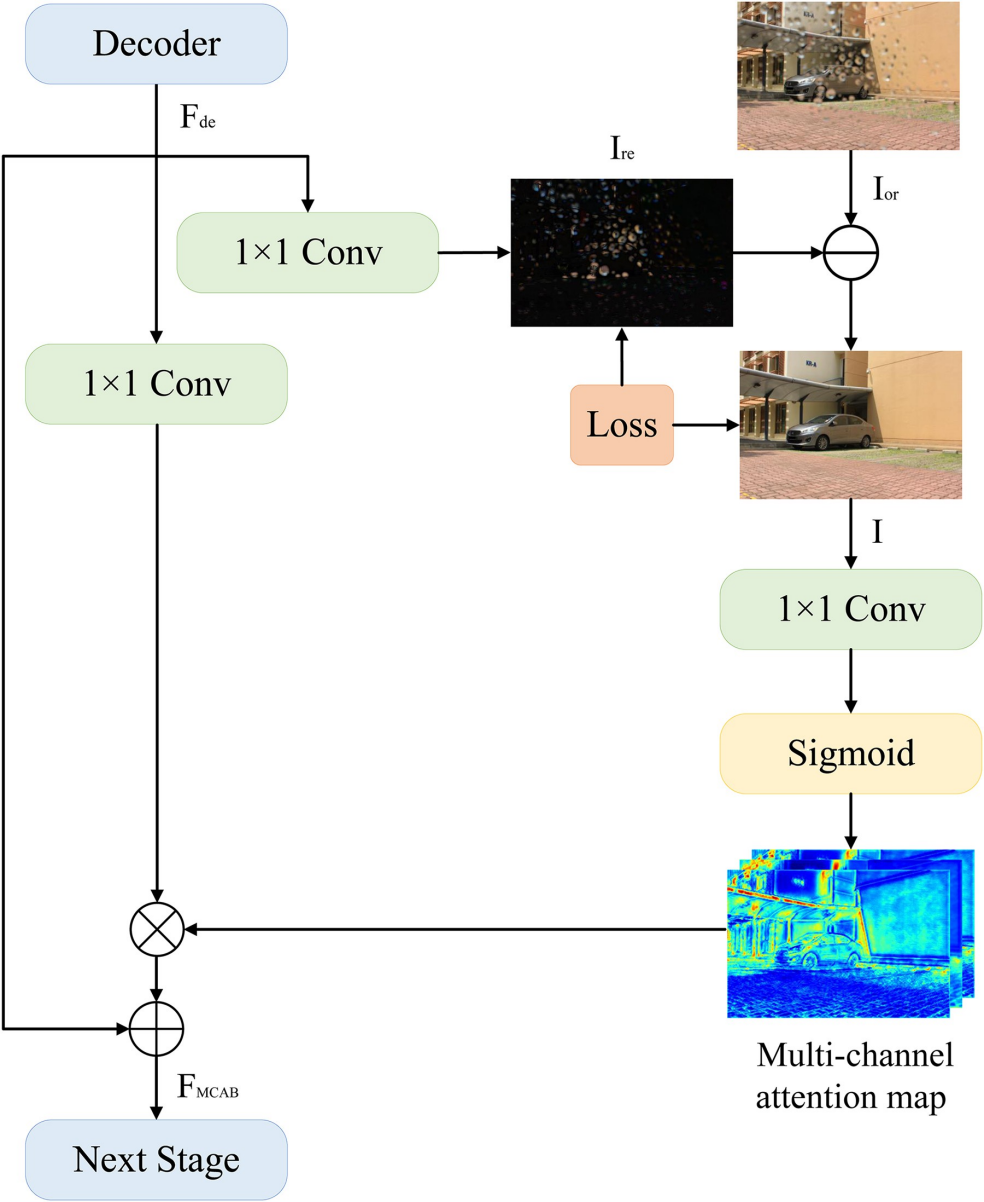


Instead of using a single-channel mask to predict the clean image, we first employ a multi-channel soft mask to assist in the restoration process. Next, we subtract the residual image from the original raindrop image to acquire the clean image under the constraint of the ground-truth image. As displayed in Fig 4, our MCAB block outputs restored rain-free images at the current stage. Synchronously, it generates a multi-channel attention map to highlight important information and fuse with the features of the next stage. We introduce MCAB in the first two stages, which considerably enhances the texture profile of the recovered images.

More specifically, the input of MCAB comes from features *F*_*de*_ extracted by the decoder and the original image *I*_*or*_. The channel of *F*_*de*_ will be adjusted from *C* to 3 through a convolution operation, which is also the acquisition process of residual image *I*_*re*_. Then *I*_*re*_ is subtracted by *I*_*or*_ to obtain the restored image *I*, which can be expressed as:

$$\begin{array}{c} I=I_{or}-I_{re}. \end{array}$$ (2)

We also perform a convolution and sigmoid operation on *I* to generate the multi-channel attention map *A*_*mc*_. It is adopted to mark the usefulness of the information in *F*_*de*_ and combine the result with *F*_*de*_ as the output feature of MCAB:

$$\begin{array}{c} F_{MCAB}=A_{mc}⊗\left(f_{c}\left(F_{de}\right)\right)+F_{de}, \end{array}$$ (3)

where *f*_*c*_ means the conv 1 × 1 operation and ⊗ means the operation of element-wise product.

#### Cascade resolution module

In the final stage, we designed a CRM module to acquire high-resolution images by extracting degradation details at various hierarchical levels. As in the bottom of Fig 2, the module consists of four resolution blocks *R*_*i*_, each of which is implemented by multiple DCABs. The first three *R*_*i*_ blocks receive features from the encoder-decoder and the output of the previous block, respectively. Afterwards, the feature scales are integrated by up-sampling operation. Each block is assembled in a cascading fashion, which can be expressed as:

$$\begin{array}{c} x_{1}=R_{1}\left(x_{0}\right)+f_{up\times 4}\left(F_{en_{1}}+F_{de_{1}}\right), \end{array}$$ (4)

$$\begin{array}{c} x_{2}=R_{2}\left(x_{1}\right)+f_{up\times 2}\left(F_{en_{2}}+F_{de_{2}}\right), \end{array}$$ (5)

$$\begin{array}{c} x_{3}=R_{3}\left(x_{2}\right)+f_{up}\left(F_{en_{3}}+F_{de_{3}}\right), \end{array}$$ (6)

$$\begin{array}{c} x_{4}=R_{4}\left(x_{3}\right), \end{array}$$ (7)

where *x*_*i*_ denotes the input of *R*_*i*+1_, *i* ∈ [0, 3]. $F_{en_{i}}$ and $F_{de_{i}}$ denotes the feature output by the encoder and decoder respectively. Also, *f*_*up*×2_ and *f*_*up*×4_ denotes the scale amplification of the features by 2 and 4 times after upsampling.

### Objective function

In order to adaptively focus on difficult-to-synthesize high-frequency components, we design an adaptive frequency loss with multiple flexible scale factors:

$$\begin{array}{c} \begin{array}{c} L_{adap}^{j}=\frac{1}{MN}\sum_{u=0}^{M-1}\sum_{v=0}^{N-1}w_{j}\left(u,v\right){\left|I(u,v)-G(u,v)\right|}^{2}, \end{array} \end{array}$$ (8)

where (*u*, *v*) means the coordinates of the spatial frequencies, and *I*(*u*, *v*) and *G*(*u*, *v*) denote the restored image and ground truth image at (*u*, *v*), respectively. *w*_*j*_(*u*, *v*)(*j* = 1, 2, 3) is the weight of the spatial frequencies with *j*-th flexible scale factors, indicated as:

$$\begin{array}{c} \begin{array}{c} w_{j}\left(u,v\right)=\mu_{j}{\left|I(u,v)-G(u,v)\right|}^{\alpha_{j}}, \end{array} \end{array}$$ (9)

where *μ*_1_, *μ*_2_ and *μ*_3_ denote trade-off coefficients of 0.5, 0.3 and 0.2, respectively. Also, *α*_0_, *α*_1_ and *α*_2_ are 1, 1/2 and 1/3, respectively, which represent flexible scale factors. Therefore, the total loss is formally as:

$$\begin{array}{c} \begin{array}{c} L_{adap}=\sum_{j=1}^{J}L_{adap}^{j},\;J=3, \end{array} \end{array}$$ (10)

where *J* denotes the number of flexible scale factors. When the frequency content at a specific coordinate belongs to the hard frequency, the function adaptively allocates a larger weight to attract the attention of the network, while the weight is reduced for easy frequencies.

Besides, we set the following loss function at each stage *S* to optimize HFNet:

$$\begin{array}{c} \begin{array}{c} L=L_{char}+\lambda_{1}L_{edge}+\lambda_{2}L_{adap}, \end{array} \end{array}$$ (11)

where λ_1_ and λ_2_ is set to 0.05 and 1 respectively [42]. $L_{char}$ denotes the Charbonnier loss [43], which approximates the distance between the recovered and clean images in pixels:

$$\begin{array}{c} \begin{array}{c} L_{char}=\sqrt{{∥I-G∥}^{2}+\varepsilon^{2}}, \end{array} \end{array}$$ (12)

where *I* and *G* denotes the restored image and ground-truth image, respectively, and constant *ε* empirically set to 10^−3^. Besides, we take edge loss $L_{edge}$ to constrain the edge texture consistency between the restored image and the ground-truth image:

$$\begin{array}{c} \begin{array}{c} L_{edge}=\sqrt{{∥\Delta I-\Delta G∥}^{2}+\varepsilon^{2}}, \end{array} \end{array}$$ (13)

where Δ donates the Laplacian operator [44].

**Algorithm 1** Training steps of the proposed method.

**Require**: *I*_*or*_: original raindrop image; *G*: ground-truth image; *S*_*i*_: number of stages; *θ*: initialization parameters of the network;

**Ensure**: *I*: restored image; *M*_*trained*_: trained raindrop removal model;

 1: Set the initialization parameters *θ*: *train*_*batch* = 4; λ_1_ = 0.05; λ_2_ = 1; *ε* = 10^−3^;

 2: **for** *epoch* = 1 to *epoch*_*num* **do**;

 3:  Recurrent feature extraction: *F*_*RPM*_ = *f*_*RPM*_(*I*_*or*_);

 4:  Parallel attention features: *F*_*PAB*_ = *f*_*PAB*_(*F*_*RPM*_);

 5:  Multi-scale features: *F*_*ED*_ = *f*_*ED*_(*F*_*PAB*_);

 6:  Multi-channel attention: *F*_*MCAB*_ = *f*_*MCAB*_(*F*_*ED*_);

 7:  Cross-stage feature fusion: $F_{S_{i}}=S_{i}\left(I_{or},F_{S_{i-1}}\right)$;

 8:  Raindrop removal: $I=f_{CRM}\left(I_{or},F_{S_{i-1}}\right)$;

 9:  Charbonnier loss: $L_{char}=\sqrt{{∥I-G∥}^{2}+\varepsilon^{2}}$;

 10:  Edge loss: $L_{edge}=\sqrt{{∥\Delta I-\Delta G∥}^{2}+\varepsilon^{2}}$;

 11:  Adaptive frequency loss: $L_{adap}=\sum_{j=1}^{J}L_{adap}^{j}$;

 12:  Total loss: $L=L_{char}+\lambda_{1}L_{edge}+\lambda_{2}L_{adap}$;

 13:  Optimize the network with L;

 14: **endfor**

 15: **return** *M*_*trained*_.

## Experiments

We evaluate the effectiveness of the proposed method on PSNR [45], SSIM [46], LPIPS [47], and FID [48] metrics. Our details of the realization, comparison experiments and ablation studies are provided below.

### Implementation details

We utilize an NVIDIA RTX 2080Ti GPU to train our HFNet. The learning rate employed for training is set to [2 × 10^−6^, 1 × 10^−4^]. *β*_1_ and *β*_2_ are set to 0.9 and 0.999 to serve as Adam’s optimization parameters. The images are cropped to 256 × 256 in a batch size of 4 as input. We finished the training with 1000 epochs. Algorithm 1 gives the specific steps for training HFNet.

### Dataset

The dataset we used contains two types. One was gathered by Qian et al. [19] which included a training set (861) and two test sets. The numbers of *Test*_*a*_ and *Test*_*b*_ are 58 and 249, respectively. This dataset contains raindrop images with different scenes, densities, and transparencies. The other is images we took in real raindrop scenes from nature to verify the generalizability of the model.

### Comparative experiments

#### Quantitative comparison

Table 1 compares the raindrop removal results of our method and existing methods on different metrics. It can be seen that our method has a considerable improvement compared to DSC [14], pix2pix [49], and DDN [49]. And our method also outperforms AttGAN [19] under the same conditions. Although PReNet [21] and MSPFN [22] utilize a cross-stage learning approach, they do not consider the multi-channel complementary feature and the frequency-domain distance between images. As shown in Table 1, our method has improved by about 1.06dB and 1.11dB on *Test*_*a*_ compared to MSPFN and UMAN, respectively. Moreover, it expands the gain on *Test*_*b*_ to 0.8dB and 2.01dB, respectively. Additionally, we note that the proposed method is inferior to AttGAN in LPIPS on *Test*_*a*_. It is mainly because *Test*_*a*_ has fewer samples and tends to overfit the network. On *Test*_*b*_ with more samples, our method performs optimally in all metrics except in FID is similar to UMAN. Such results also indicates the strength of our method in generalization.

**Table 1:** Quantitative evaluation results on *Test*_*a*_ and *Test*_*b*_.

Dataset	Metric	DSC [13]	pix2pix [49]	DDN [50]	AttGAN [19]	PReNet [21]	MSPFN [22]	UMAN [20]	Ours
*Test*_*a*_	PSNR↑	24.13	26.79	29.12	31.57	31.54	31.52	31.47	**32.58**
	SSIM↑	0.8548	0.8644	0.8913	0.9023	0.9310	0.9287	0.9235	**0.9422**
	LPIPS↓	0.185	0.179	0.125	**0.048**	0102	0.113	0.063	0.054
	FID↓	120.667	94.591	77.114	52.808	62.570	56.429	39.080	**32.842**
*Test*_*b*_	PSNR↑	23.13	23.50	24.52	24.92	25.92	26.56	25.35	**27.36**
	SSIM↑	0.7321	0.7150	0.7702	0.8090	0.8435	0.8466	0.8197	**0.8527**
	LPIPS↓	0.168	0.409	0.358	0.213	0.118	0.126	1.120	**0.094**
	FID↓	67.757	69.760	67.120	43.551	36.611	34.510	21.583	**21.568**

#### Qualitative comparison

**Fig 5:**
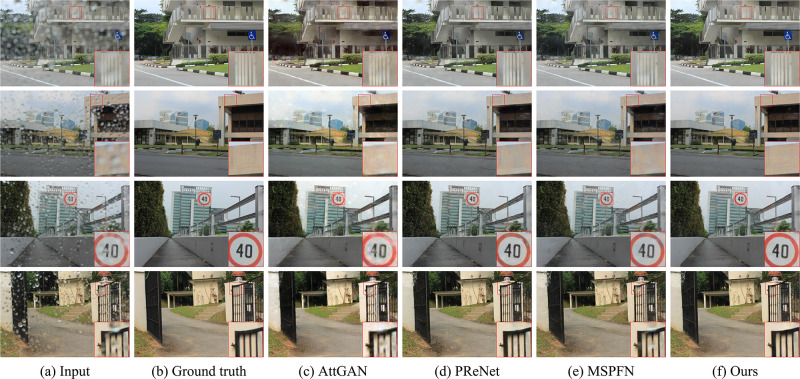


Fig 5 shows the visual results of various deraining methods for raindrops with different shapes, densities, and transparencies. It can be seen that all methods achieve satisfactory removal effects for the image with small density raindrops, but our approach has better authenticity in global and local perception. Since the shape and transparency of raindrops become diverse with increasing density, there will be slight artifacts in the comparison method when processing images with higher density raindrops. While, our method does not suffer significantly in terms of visual quality. The primary reason is that our soft mask learns more accurate raindrop shape and transparency during training. For removing small shapes and highly transparent raindrops, AttGAN and MSPFN leaves a noticeable amount of unremoved raindrops. PReNet also appears larger artifacts and distortion. In contrast, the images restored by our strategy have sharper textures.

**Fig 6:**
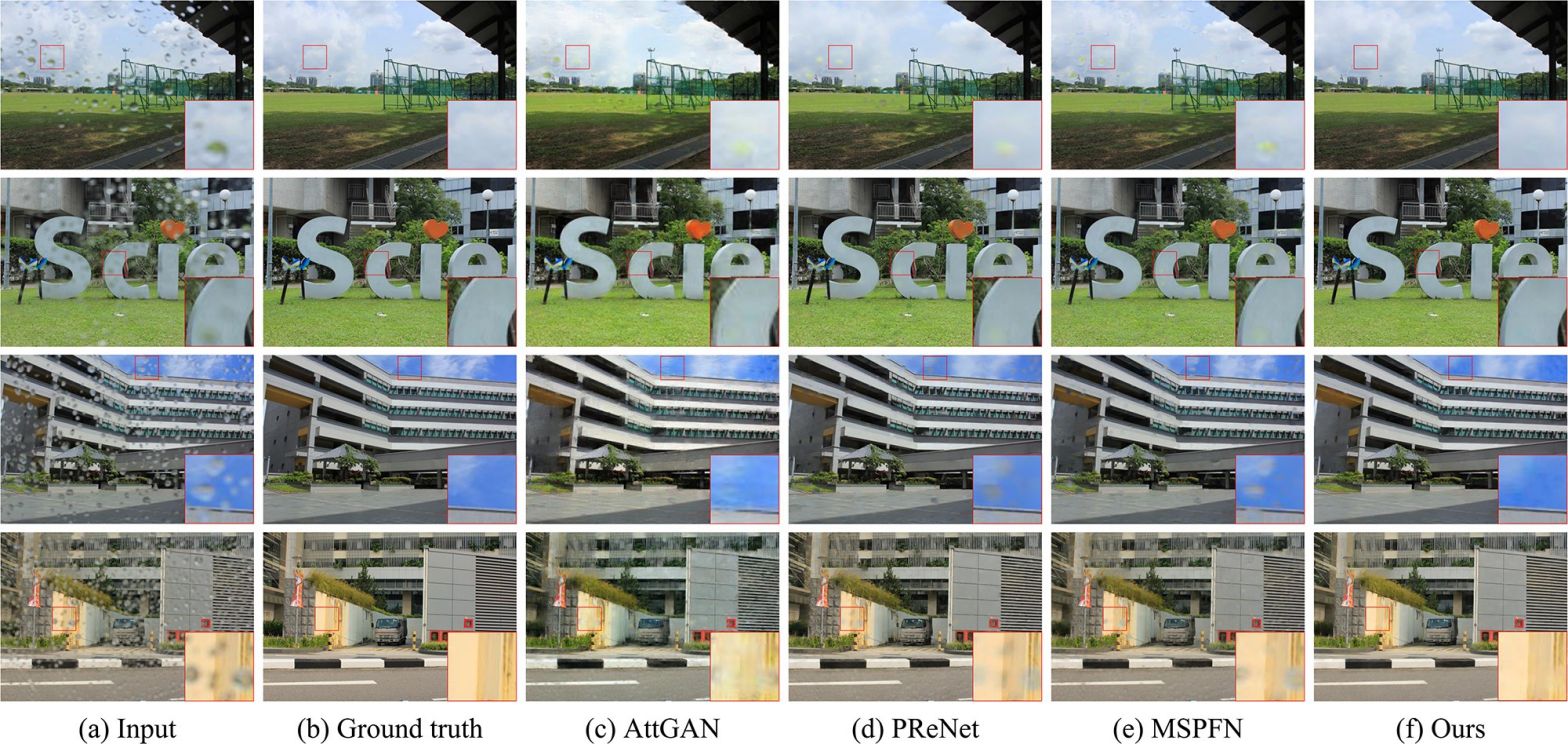


Fig 6 demonstrates the advantages of the proposed method in processing images in which the color and content of raindrops are changed by background targets. As can be seen from the figure, albeit the compared methods can remove most of the raindrops, none of them can recover the regions with color-varying or content-changing well. Conversely, our approach has a tremendous advantage in removing raindrops with such changes.

#### Results on natural images

**Fig 7:**
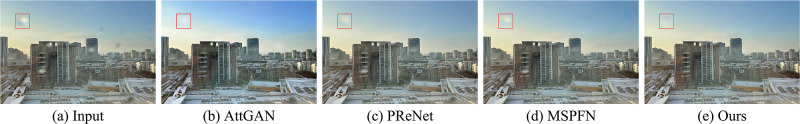


We further verify the generalization of our method on natural raindrop images. As shown in Fig 7, our method exhibits fewer artifacts for raindrops with different shapes, colors, and transparency. This illustrates the better generalization of our proposed method. We also find that for some of the rainy regions similar to rain streaks, our method accomplishes the restoration with high-quality image details. It also indicates that our method is applicable to the removal of rain streaks.

### Ablation studies

#### Effectiveness of each component

To verify the contribution of each module in the network, we compare the performance of the entire HFNet with the network after removing a particular module. We set up the following experiments: N-1 (without cross-stage connections (CSC)), N-2 (without CRM), N-3 (without MCAB), N-4 (without JAM), N-5 (without RPM), N-6 (without DSAB), N-7 (without DCAB). All experiments are performed under the same conditions except for the removed modules in each experiment.

**Fig 8:**
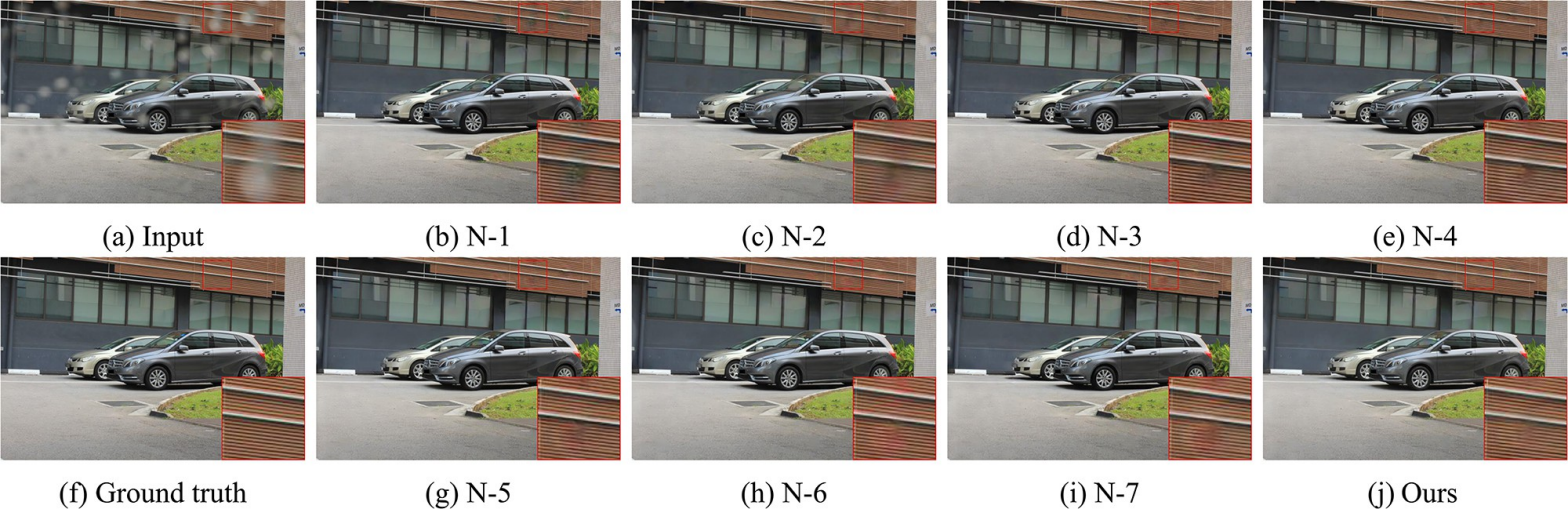


**Fig 9:**
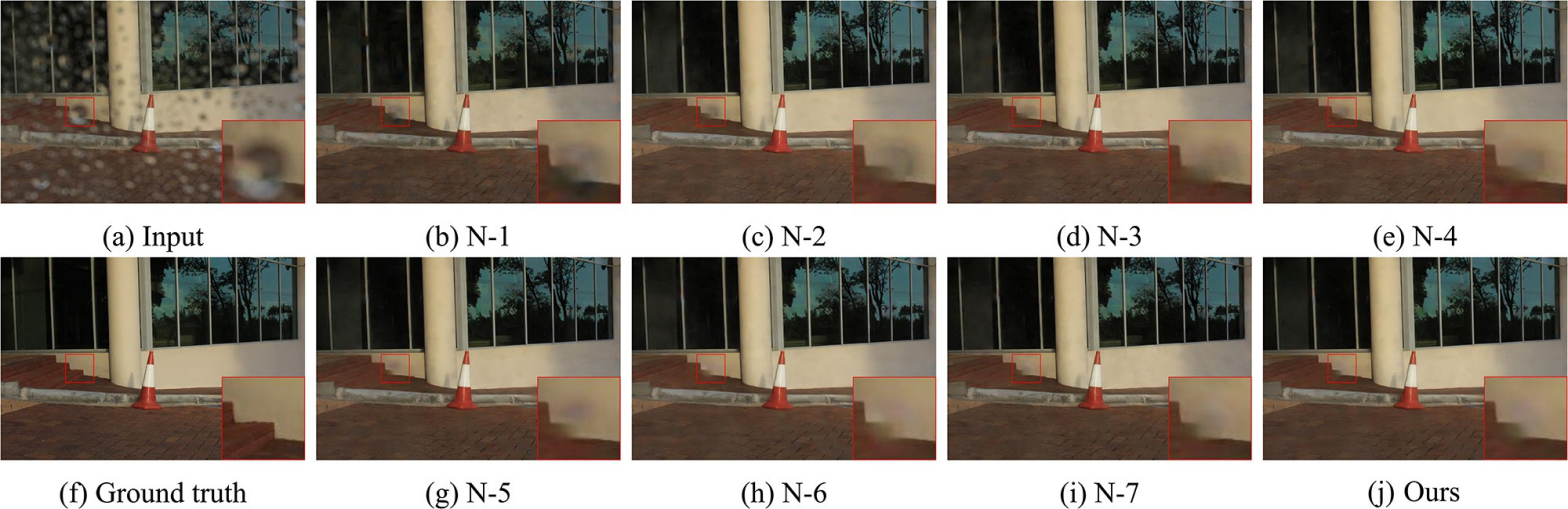


From Table 2, it can be seen a slight degradation in the performance of the network after removing DCAB and DSAB from HFNet. Since RPM provides reliable image features and JAM focuses on important region in terms of channel and spatial dimensions, the effect on the raindrop removal results is greater after removing RPM and JAM. Meanwhile, there is a significant performance reduction after removing MCAB, CRM and CSC. This is primarily due to the lack of some complementary feature details under multi-channel and cross-stage. The PSNR at this setting is correspondingly reduced by about 0.61dB, 0.93dB, and 1.04dB on *Test*_*a*_. This result reveals the vital contribution of these three modules to the whole network. The visual comparison results are shown in Figs 8 and 9.

**Table 2:** Results of ablation studies on *Test*_*a*_ and *Test*_*b*_. “✔” and “×” refer to with and without the module.

Network	DCAB	DSAB	RPM	JAM	MCAB	CRM	CSC	*Test*_*a*_	
								PSNR ↑	SSIM ↑
N-1	✔	✔	✔	✔	✔	✔	×	31.54	0.9224
N-2	✔	✔	✔	✔	✔	×	✔	31.65	0.9325
N-3	✔	✔	✔	✔	×	✔	✔	31.97	0.9368
N-4	✔	✔	✔	×	✔	✔	✔	32.38	0.9420
N-5	✔	✔	×	✔	✔	✔	✔	32.36	0.9419
N-6	✔	×	✔	✔	✔	✔	✔	32.43	0.9421
N-7	×	✔	✔	✔	✔	✔	✔	32.47	0.9421
Ours	✔	✔	✔	✔	✔	✔	✔	**32.58**	**0.9422**

#### Number of sub-modules in CRM

Besides, we verify the impact of the number of sub-modules in CRM on raindrop removal. As shown in Table 3, with the increase of *N*_*s*_, the effectiveness of raindrop removal presents an upward trend. The network achieves the best performance when *N*_*s*_ is 5. Furthermore, the difference between the raindrop removal results with *N*_*s*_ set to 4 and 5 is not significant, while taking 5 in *N*_*s*_ will increase the complexity. Considering the computational efficiency of the network, we set *N*_*s*_ of CSFNet as 4.

**Table 3:** Ablation studies of the number of sub-modules (*N*_*s*_) in CRM.

Dataset	Metric	*N*_*s*_ = 1	*N*_*s*_ = 2	*N*_*s*_ = 3	*N*_*s*_ = 4	*N*_*s*_ = 5
*Test*_*a*_	PSNR↑	31.73	32.19	32.44	32.58	**32.60**
	SSIM↑	0.9356	0.9393	0.9406	**0.9422**	**0.9422**
*Test*_*b*_	PSNR↑	26.92	27.12	27.26	27.35	**27.36**
	SSIM↑	0.8498	0.8514	0.8520	**0.8527**	**0.8527**
–	Param (M)	2.93	3.12	3.30	3.49	3.67
	FLOPS (G)	22.61	25.55	28.51	31.49	34.52

#### Effectiveness of loss function

**Fig 10:**
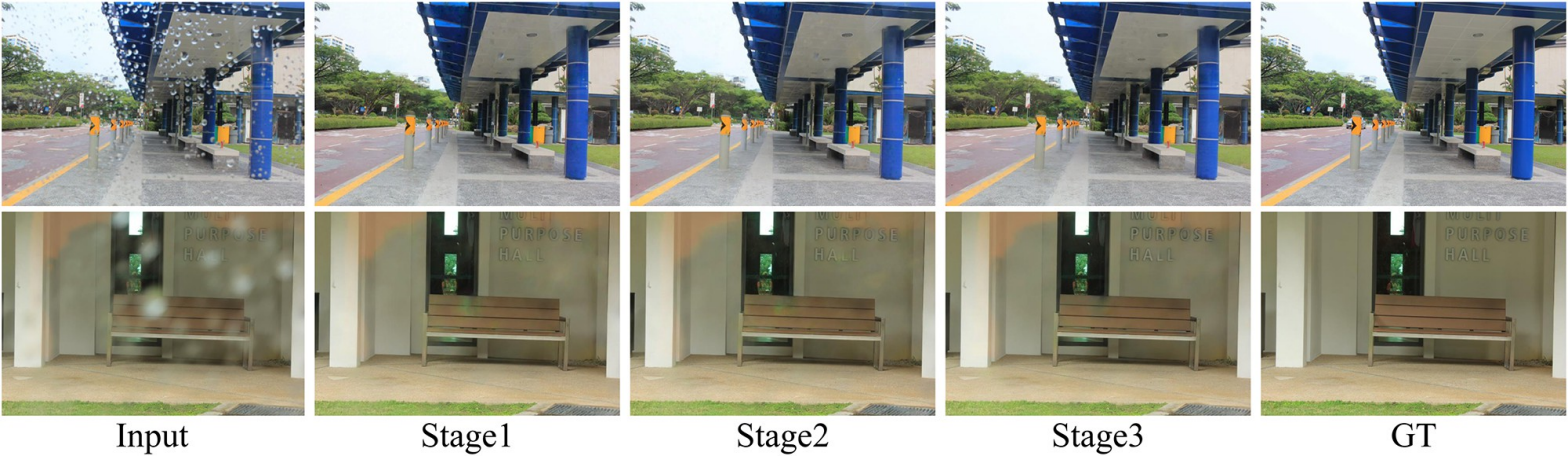


We further performed ablation studies on the loss function in Table 4. It can be noticed that the performance of the model gradually improves with the addition of different losses. The experimental results show that the adaptive frequency loss with multi-flexible scale factors can improve the authenticity of the restored image. The reason for this is that the constraints at different scales present complementary effects. We also visualize the output results of each stagein in Fig 10. It can be seen that the visual quality of the restored image gradually improves as the number of stages increases.

**Table 4:** Ablation studies of the loss function on *Test*_*a*_ and *Test*_*b*_.

Loss	*Test*_*a*_		*Test*_*b*_	
	PSNR↑	SSIM↑	PSNR↑	SSIM↑
$L_{char}$	31.87	0.9361	26.87	0.8497
$L_{char}+L_{edge}$	32.28	0.9412	27.12	0.8518
$L_{char}+L_{edge}+L_{adap}\left(\alpha =1\right)$	32.51	0.9420	27.33	0.8526
$L_{char}+L_{edge}+L_{adap}\left(\alpha =1/2\right)$	32.51	0.9421	27.31	0.8525
$L_{char}+L_{edge}+L_{adap}\left(\alpha =1/3\right)$	32.50	0.9418	27.31	0.8525
Ours	**32.58**	**0.9422**	27.36	0.8527

## Conclusion

In this paper, we propose a novel frequency-aware Hierarchical Fusion Network (HFNet) for raindrop image restoration. The exploration of frequency representation allows the network to extract rich and accurate raindrop features. Meanwhile, we utilize the synergistic cooperation of hierarchical fusion and calibrated attention mechanism to preserve a more complete background structure. We verify the superiority of the proposed method on real raindrop images and the generalization in natural scenes. The experimental results indicate that our strategy is capable of reconstructing more convincing images in the presence of diverse appearances and background-affected raindrops. In future work, we will investigate the impact of external potential factors (atmospheric light, etc) on raindrop removal.

## Supporting information

S1 Data (ZIP)

S2 Data (ZIP)
