# Supplementary figures and images for: Frequency-oriented hierarchical fusion network for single image raindrop removal (part 2 of 2)
Source: PLoS One. 2024 May 23;19(5):e0301439. doi: 10.1371/journal.pone.0301439 (PMC11115234; doi:10.1371/journal.pone.0301439)

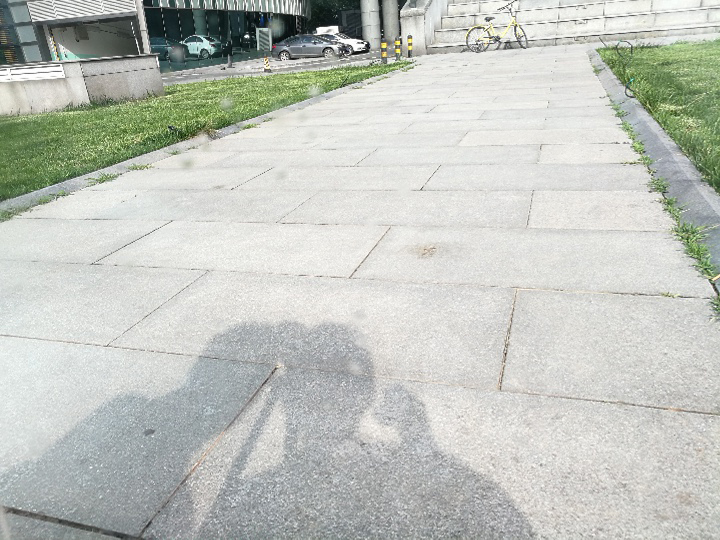

Supplement: S1 Data — (ZIP) [file pone.0301439.s001.zip › test_b/data/138_rain.jpg]

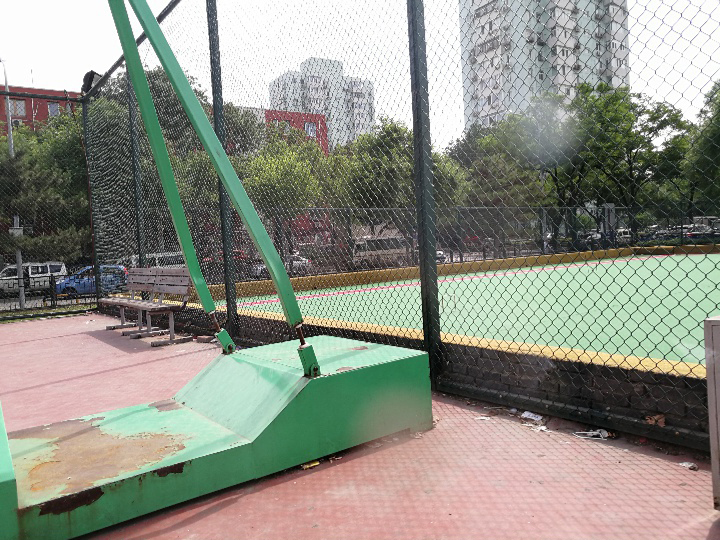

Supplement: S1 Data — (ZIP) [file pone.0301439.s001.zip › test_b/data/139_rain.jpg]

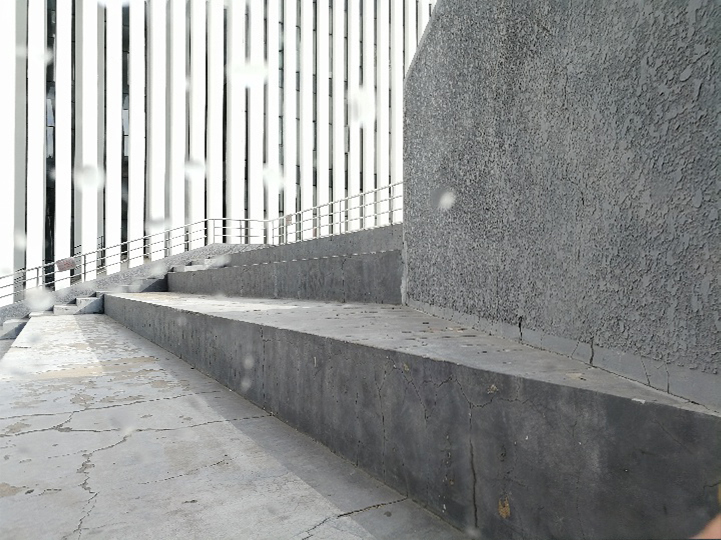

Supplement: S1 Data — (ZIP) [file pone.0301439.s001.zip › test_b/data/13_rain.jpg]

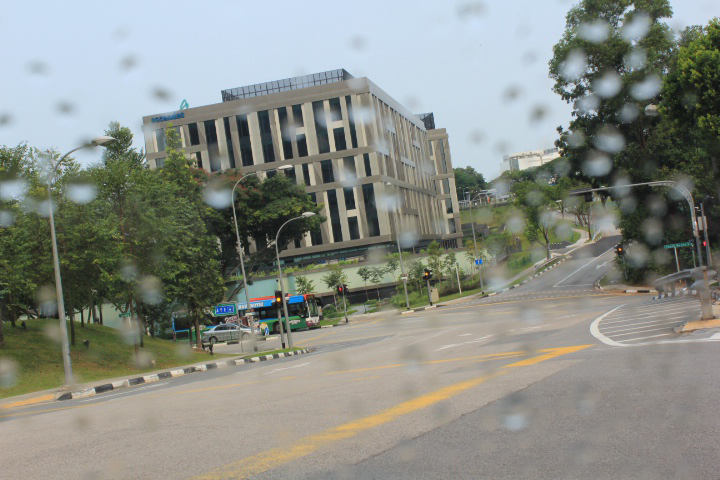

Supplement: S1 Data — (ZIP) [file pone.0301439.s001.zip › test_b/data/140_rain.jpg]

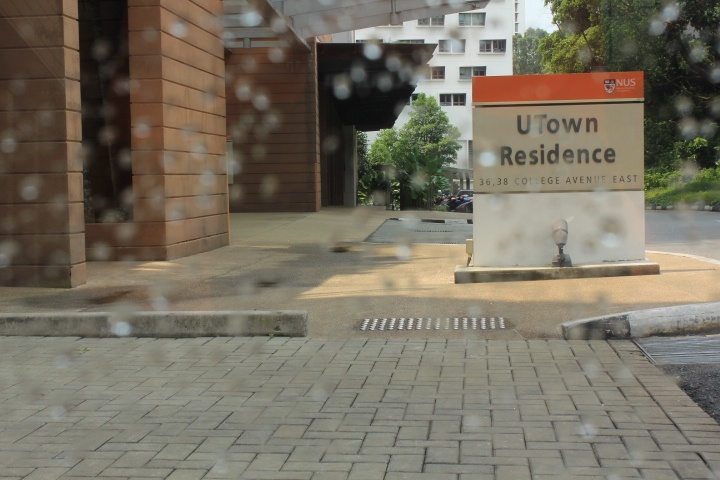

Supplement: S1 Data — (ZIP) [file pone.0301439.s001.zip › test_b/data/141_rain.jpg]

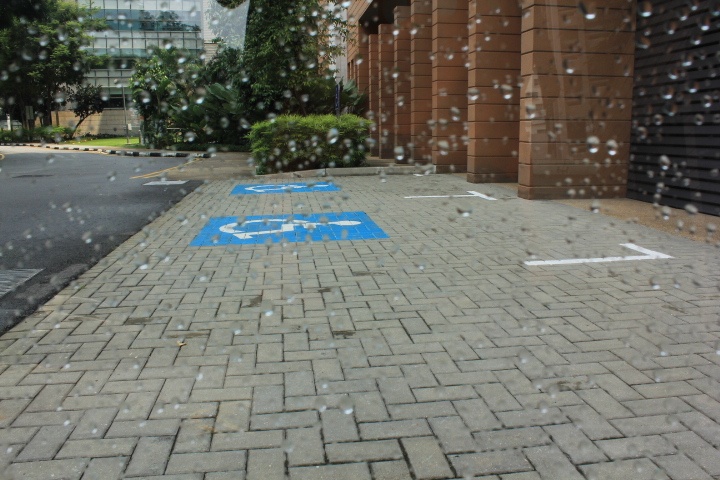

Supplement: S1 Data — (ZIP) [file pone.0301439.s001.zip › test_b/data/142_rain.jpg]

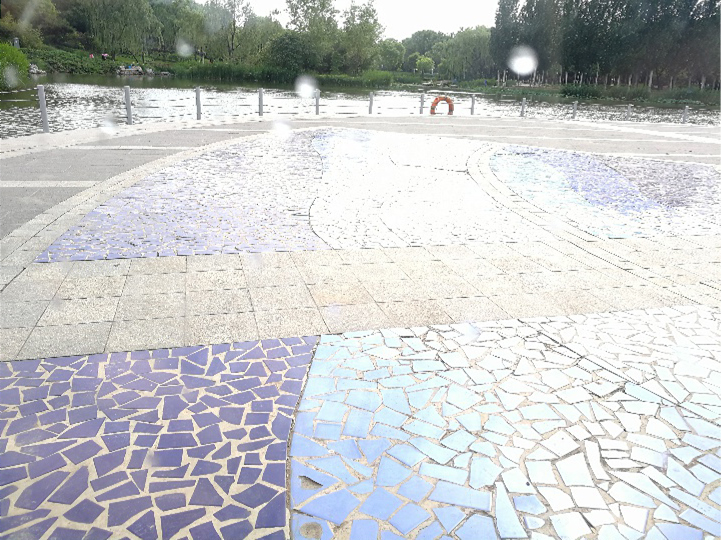

Supplement: S1 Data — (ZIP) [file pone.0301439.s001.zip › test_b/data/143_rain.jpg]

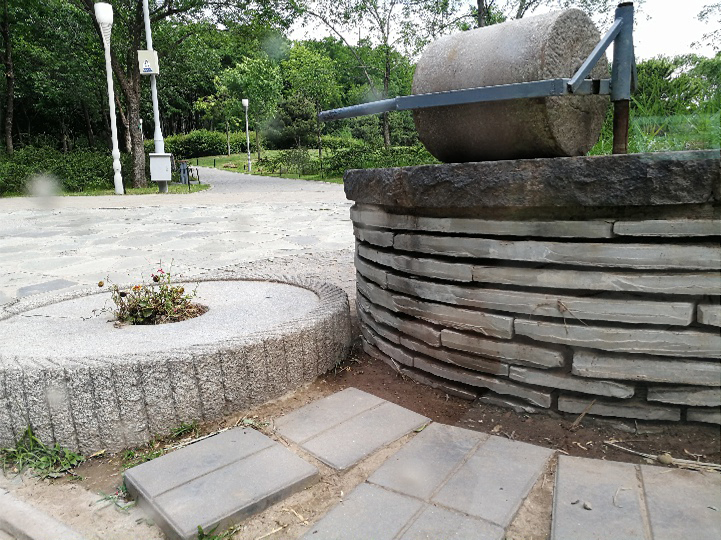

Supplement: S1 Data — (ZIP) [file pone.0301439.s001.zip › test_b/data/144_rain.jpg]

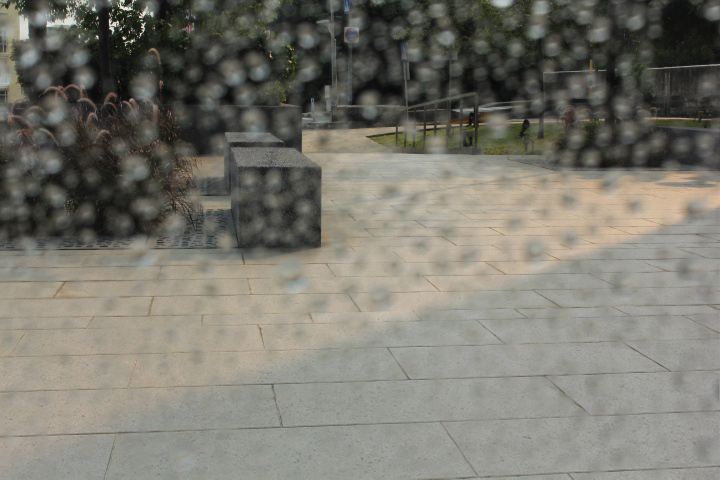

Supplement: S1 Data — (ZIP) [file pone.0301439.s001.zip › test_b/data/145_rain.jpg]

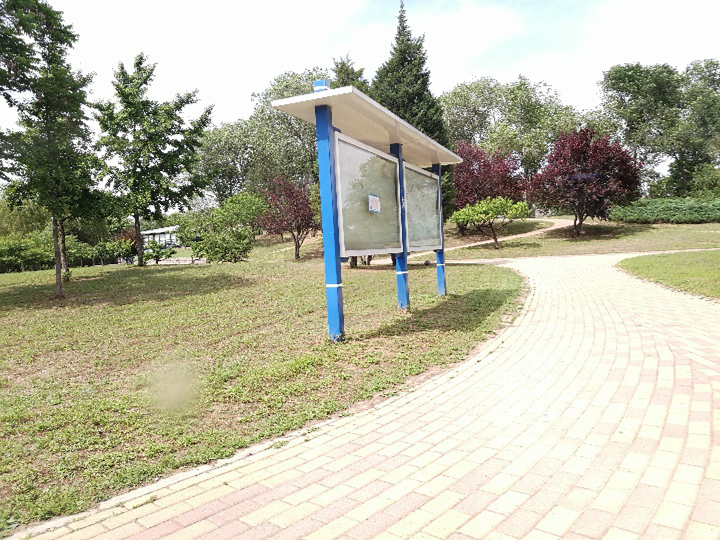

Supplement: S1 Data — (ZIP) [file pone.0301439.s001.zip › test_b/data/146_rain.jpg]

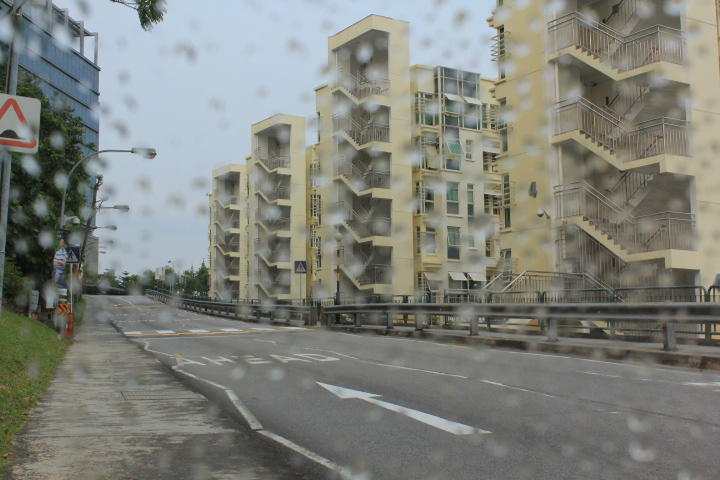

Supplement: S1 Data — (ZIP) [file pone.0301439.s001.zip › test_b/data/147_rain.jpg]

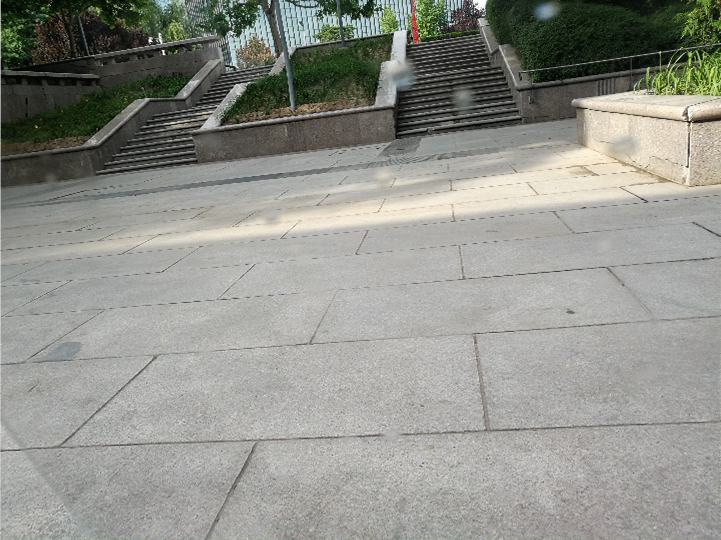

Supplement: S1 Data — (ZIP) [file pone.0301439.s001.zip › test_b/data/148_rain.jpg]

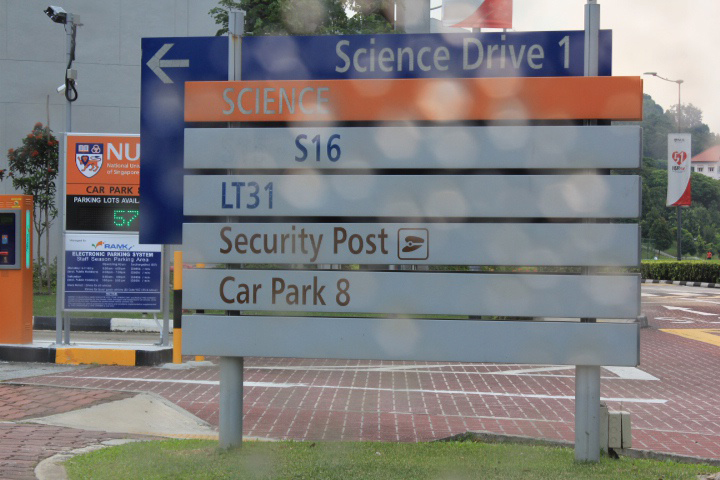

Supplement: S1 Data — (ZIP) [file pone.0301439.s001.zip › test_b/data/149_rain.jpg]

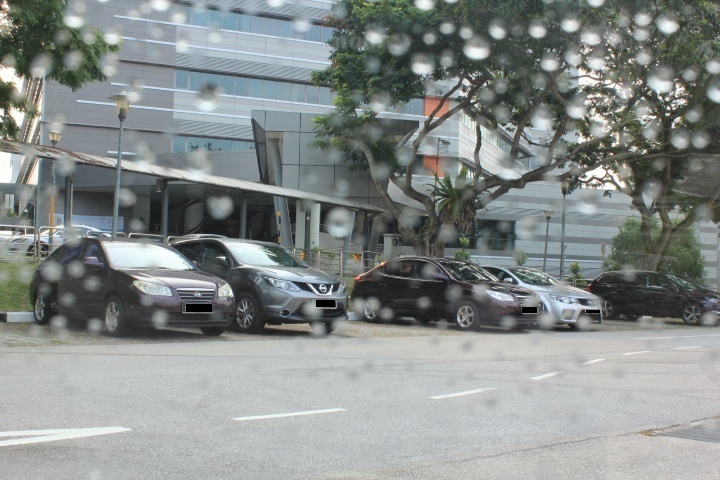

Supplement: S1 Data — (ZIP) [file pone.0301439.s001.zip › test_b/data/14_rain.jpg]

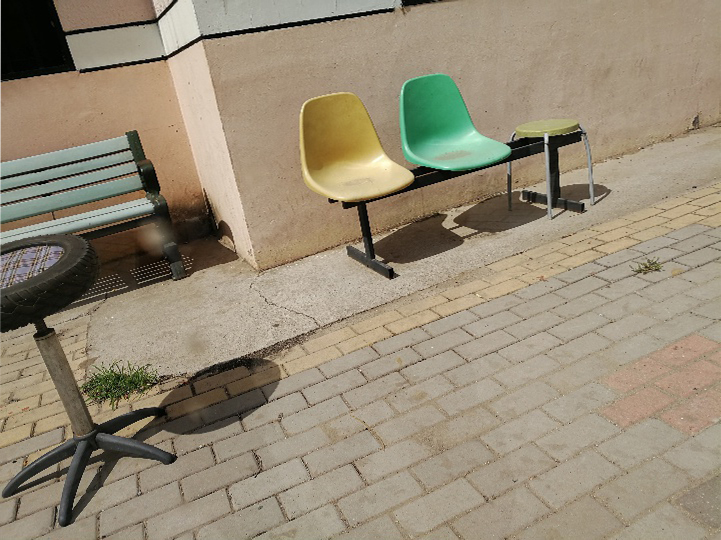

Supplement: S1 Data — (ZIP) [file pone.0301439.s001.zip › test_b/data/150_rain.jpg]

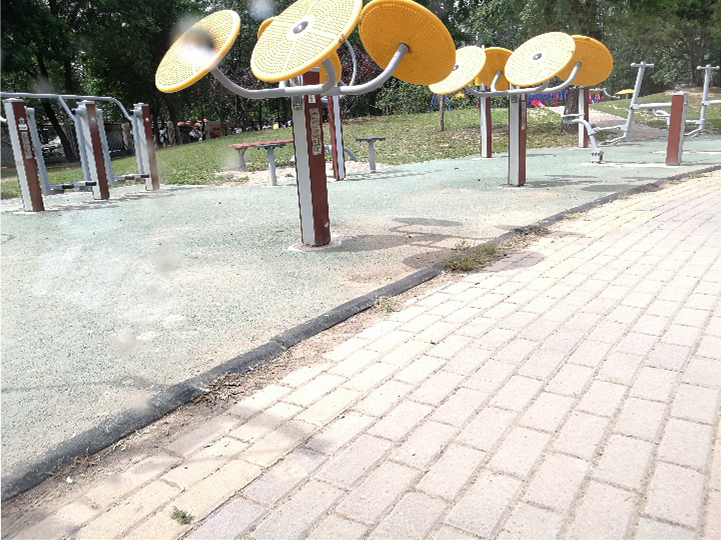

Supplement: S1 Data — (ZIP) [file pone.0301439.s001.zip › test_b/data/151_rain.jpg]

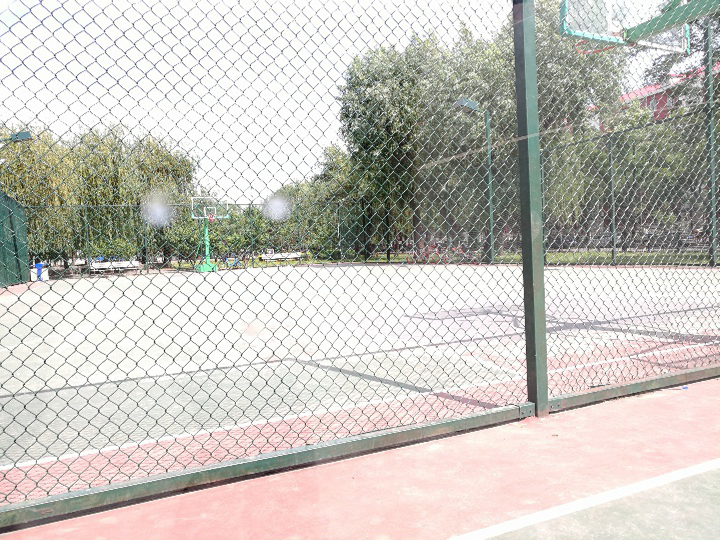

Supplement: S1 Data — (ZIP) [file pone.0301439.s001.zip › test_b/data/152_rain.jpg]

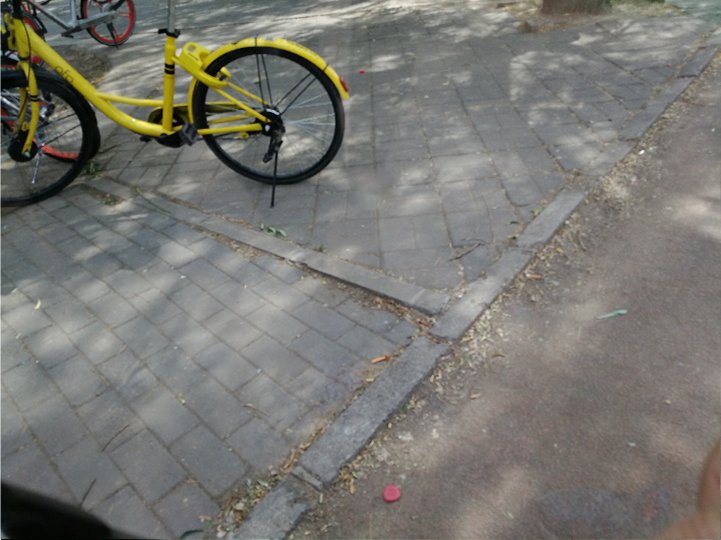

Supplement: S1 Data — (ZIP) [file pone.0301439.s001.zip › test_b/data/153_rain.jpg]

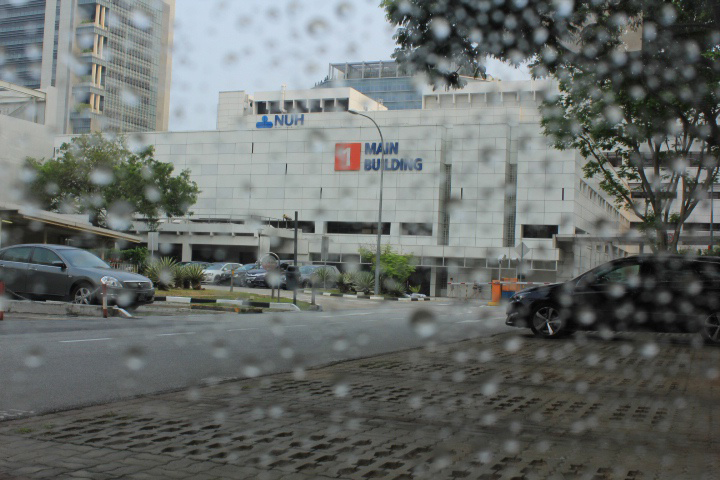

Supplement: S1 Data — (ZIP) [file pone.0301439.s001.zip › test_b/data/154_rain.jpg]

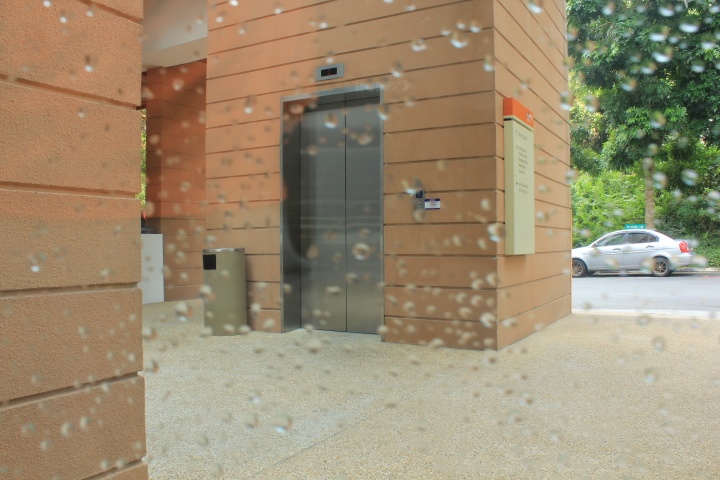

Supplement: S1 Data — (ZIP) [file pone.0301439.s001.zip › test_b/data/155_rain.jpg]

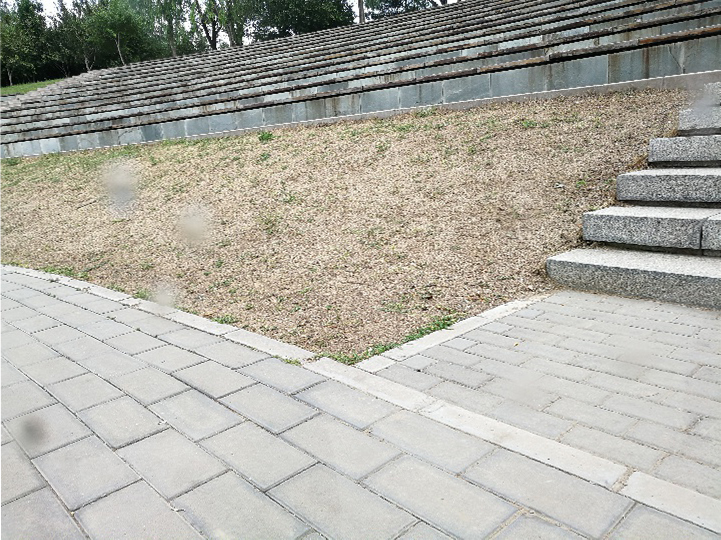

Supplement: S1 Data — (ZIP) [file pone.0301439.s001.zip › test_b/data/156_rain.jpg]

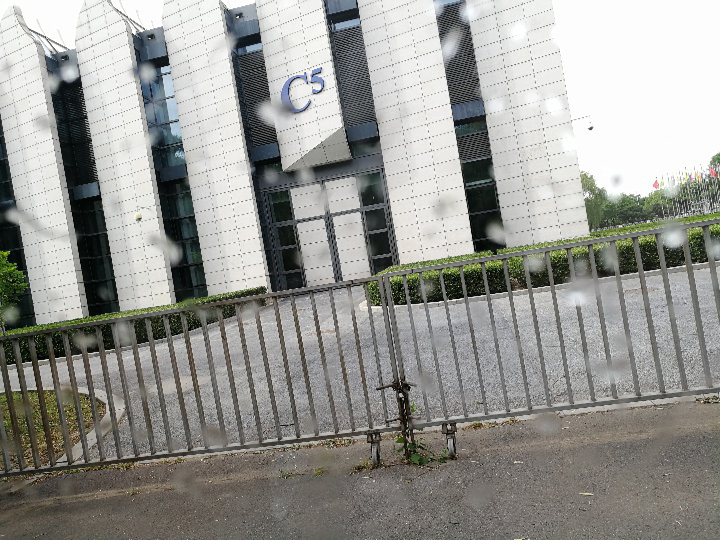

Supplement: S1 Data — (ZIP) [file pone.0301439.s001.zip › test_b/data/157_rain.jpg]

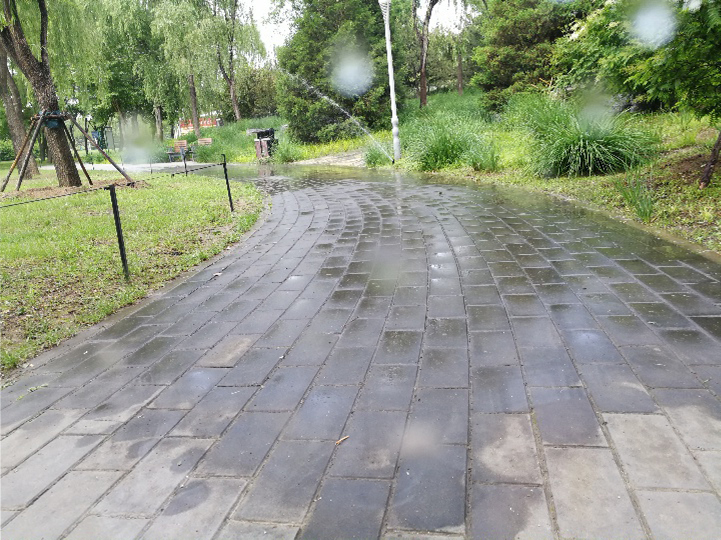

Supplement: S1 Data — (ZIP) [file pone.0301439.s001.zip › test_b/data/158_rain.jpg]

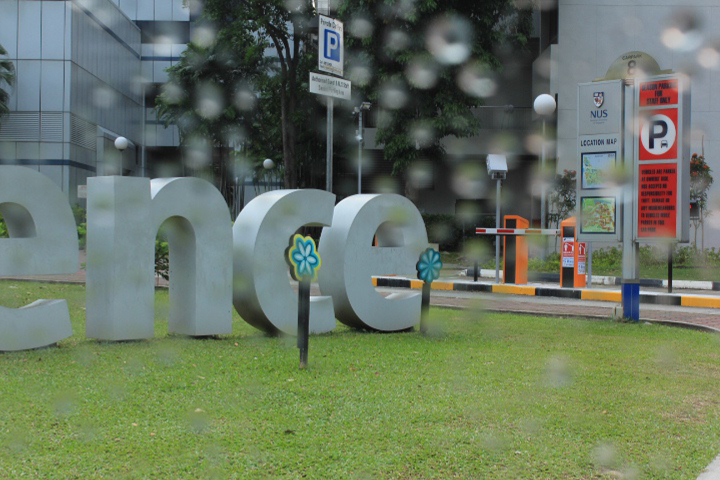

Supplement: S1 Data — (ZIP) [file pone.0301439.s001.zip › test_b/data/159_rain.jpg]

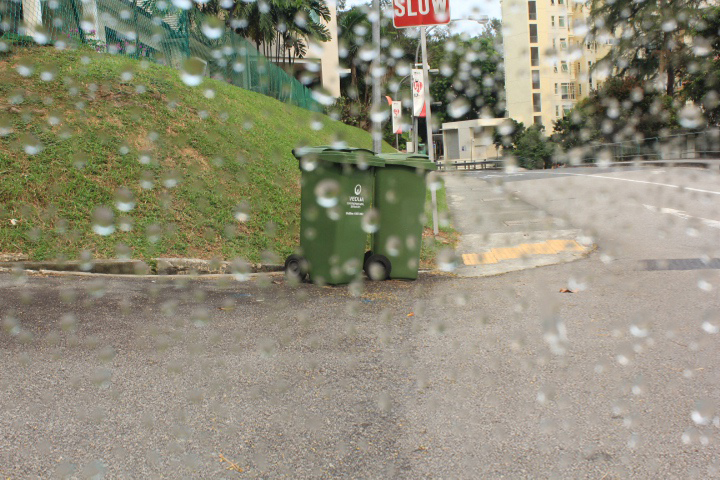

Supplement: S1 Data — (ZIP) [file pone.0301439.s001.zip › test_b/data/15_rain.jpg]

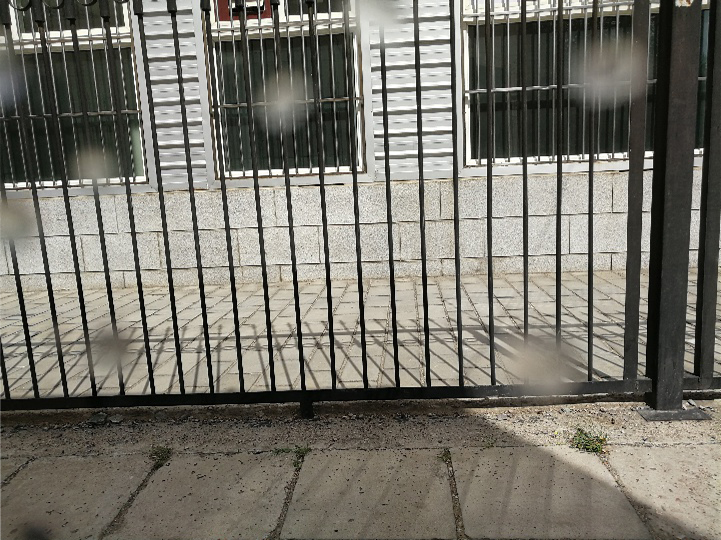

Supplement: S1 Data — (ZIP) [file pone.0301439.s001.zip › test_b/data/160_rain.jpg]

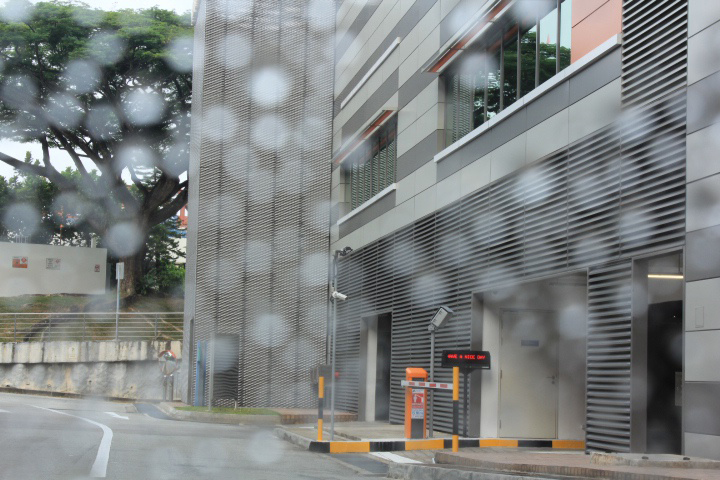

Supplement: S1 Data — (ZIP) [file pone.0301439.s001.zip › test_b/data/161_rain.jpg]

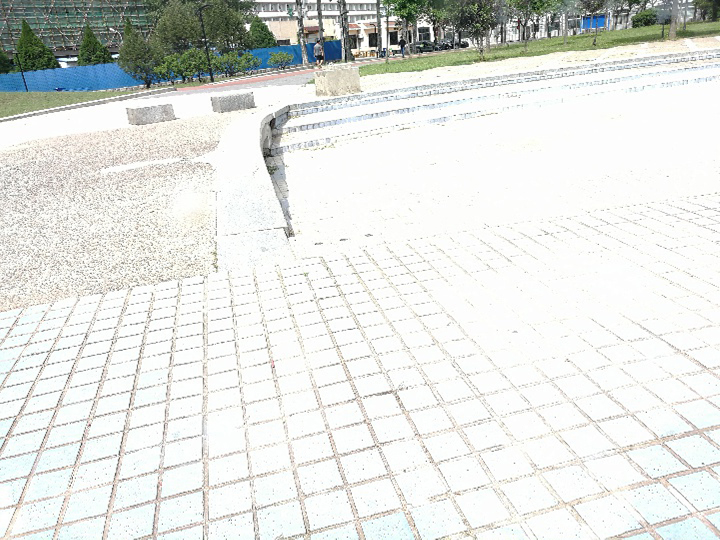

Supplement: S1 Data — (ZIP) [file pone.0301439.s001.zip › test_b/data/162_rain.jpg]

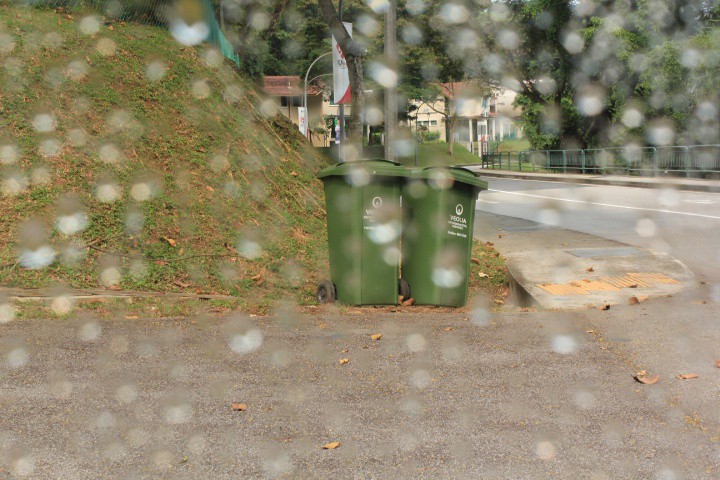

Supplement: S1 Data — (ZIP) [file pone.0301439.s001.zip › test_b/data/163_rain.jpg]

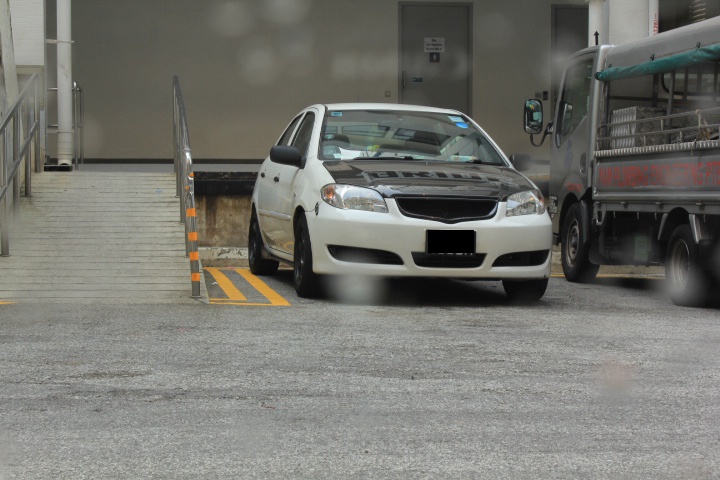

Supplement: S1 Data — (ZIP) [file pone.0301439.s001.zip › test_b/data/164_rain.jpg]

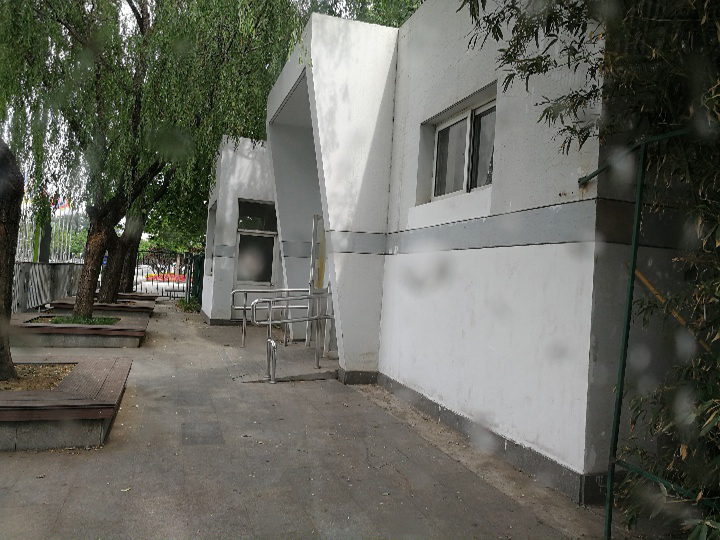

Supplement: S1 Data — (ZIP) [file pone.0301439.s001.zip › test_b/data/165_rain.jpg]

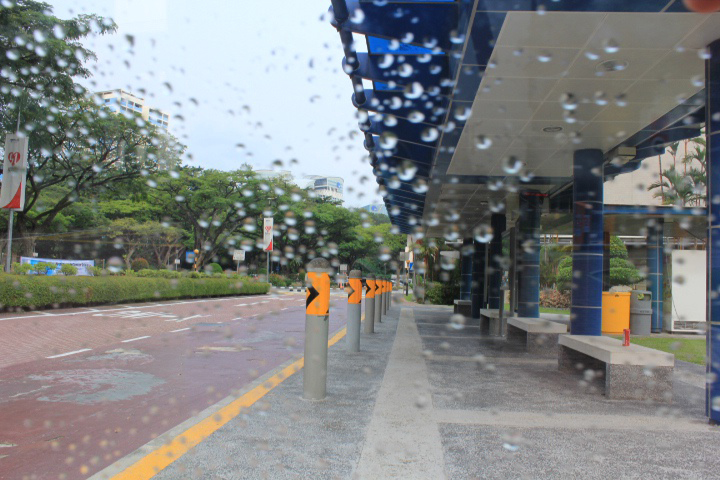

Supplement: S1 Data — (ZIP) [file pone.0301439.s001.zip › test_b/data/166_rain.jpg]

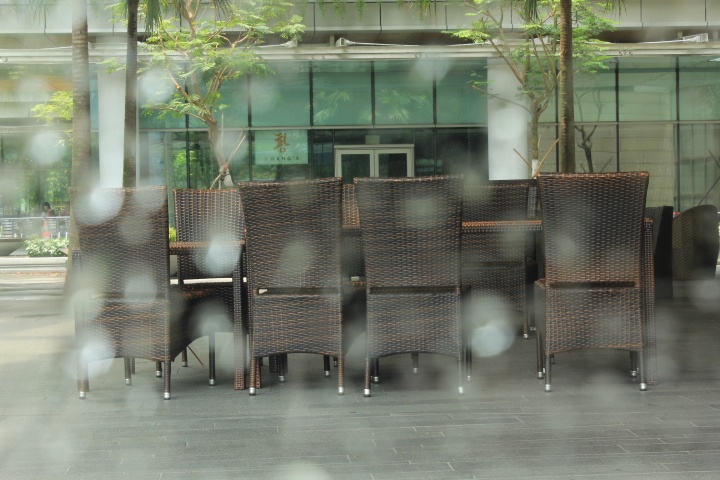

Supplement: S1 Data — (ZIP) [file pone.0301439.s001.zip › test_b/data/167_rain.jpg]

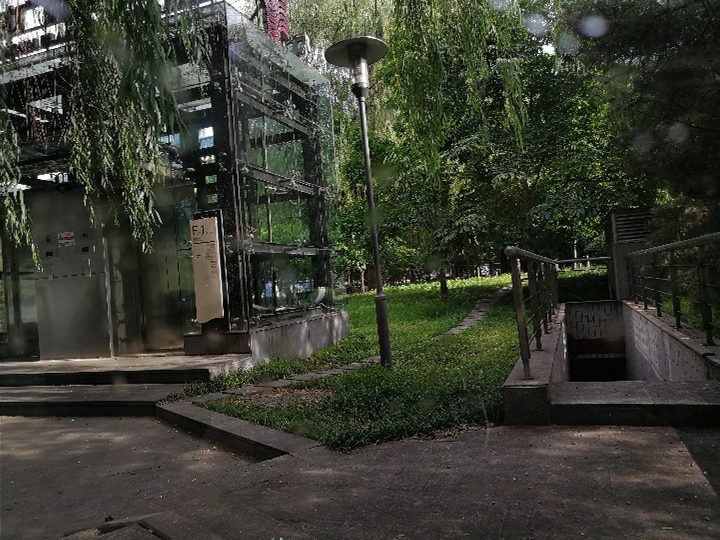

Supplement: S1 Data — (ZIP) [file pone.0301439.s001.zip › test_b/data/168_rain.jpg]

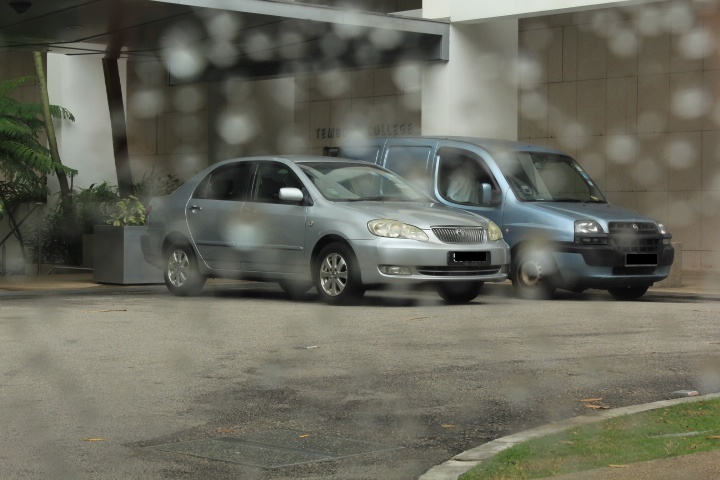

Supplement: S1 Data — (ZIP) [file pone.0301439.s001.zip › test_b/data/169_rain.jpg]

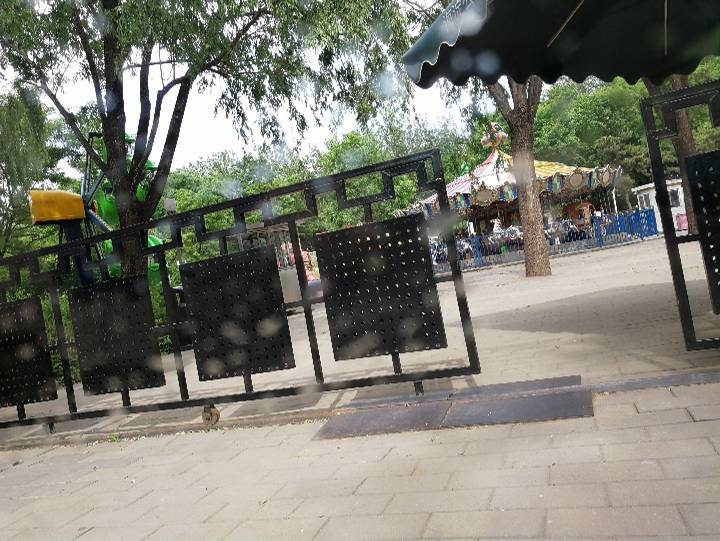

Supplement: S1 Data — (ZIP) [file pone.0301439.s001.zip › test_b/data/16_rain.jpg]

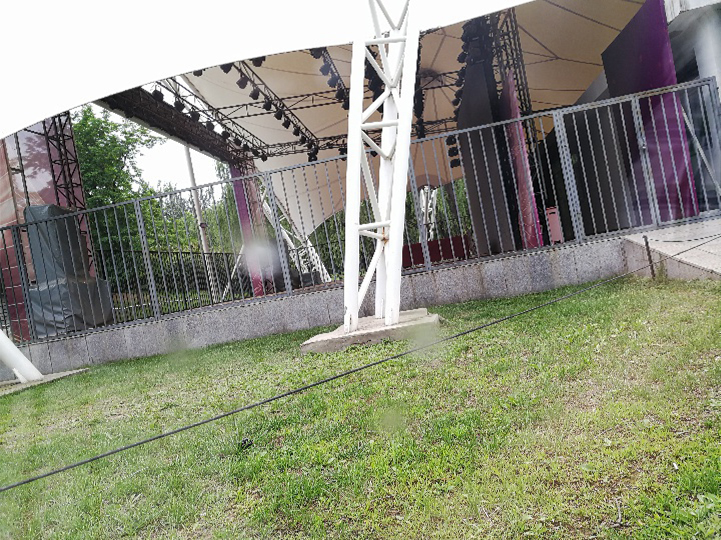

Supplement: S1 Data — (ZIP) [file pone.0301439.s001.zip › test_b/data/170_rain.jpg]

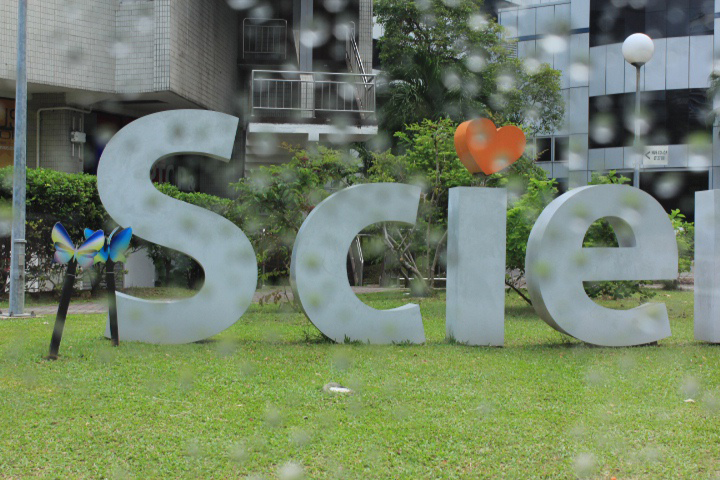

Supplement: S1 Data — (ZIP) [file pone.0301439.s001.zip › test_b/data/171_rain.jpg]

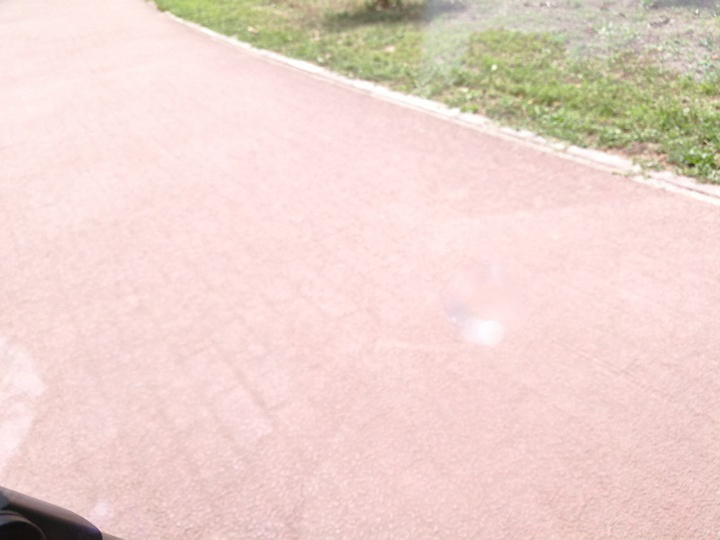

Supplement: S1 Data — (ZIP) [file pone.0301439.s001.zip › test_b/data/172_rain.jpg]

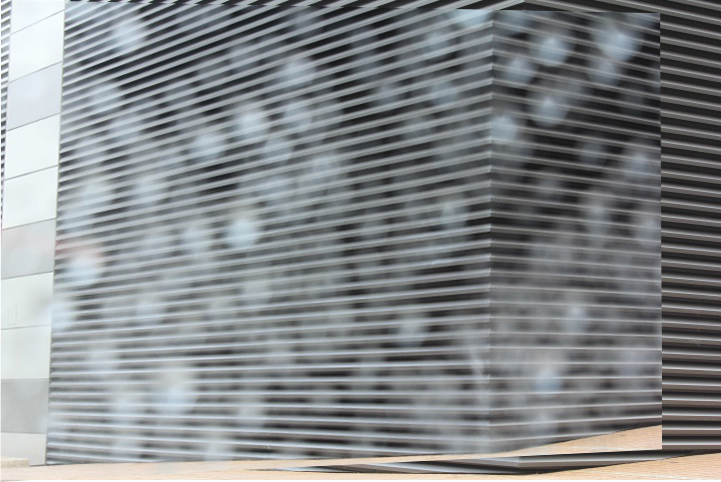

Supplement: S1 Data — (ZIP) [file pone.0301439.s001.zip › test_b/data/173_rain.jpg]

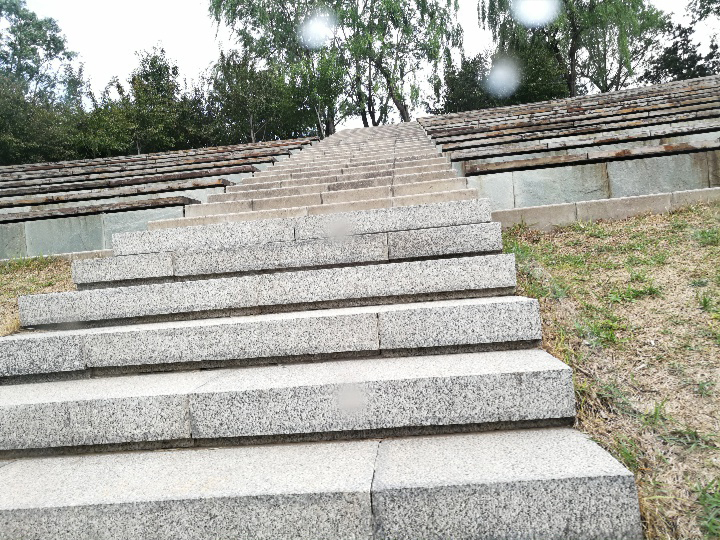

Supplement: S1 Data — (ZIP) [file pone.0301439.s001.zip › test_b/data/174_rain.jpg]

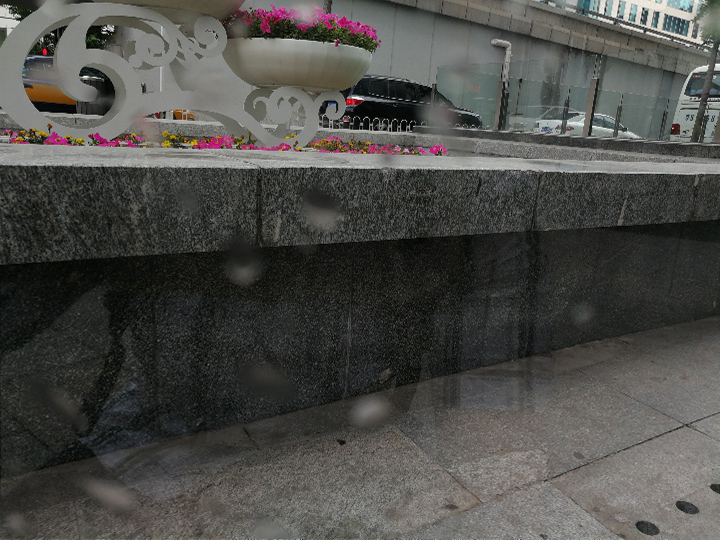

Supplement: S1 Data — (ZIP) [file pone.0301439.s001.zip › test_b/data/175_rain.jpg]

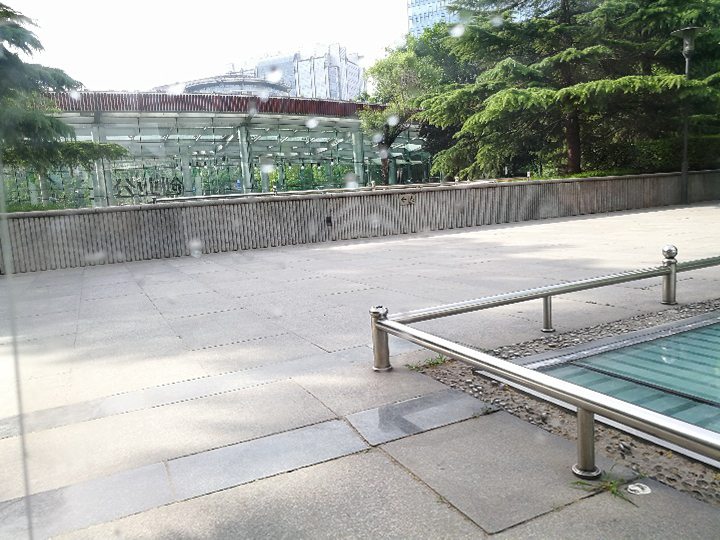

Supplement: S1 Data — (ZIP) [file pone.0301439.s001.zip › test_b/data/176_rain.jpg]

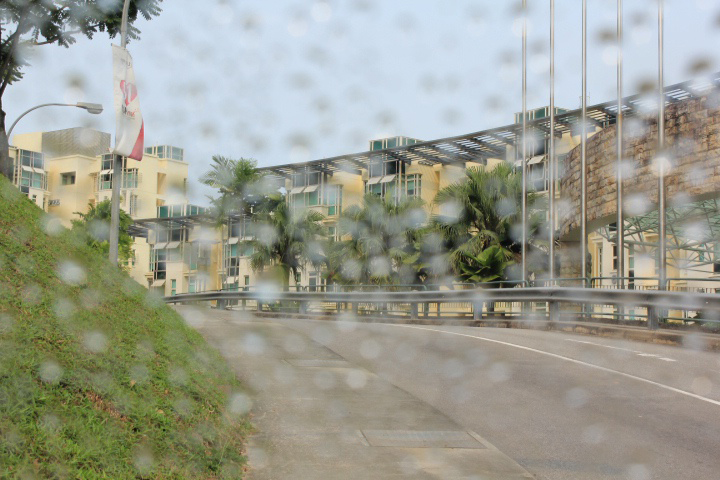

Supplement: S1 Data — (ZIP) [file pone.0301439.s001.zip › test_b/data/177_rain.jpg]

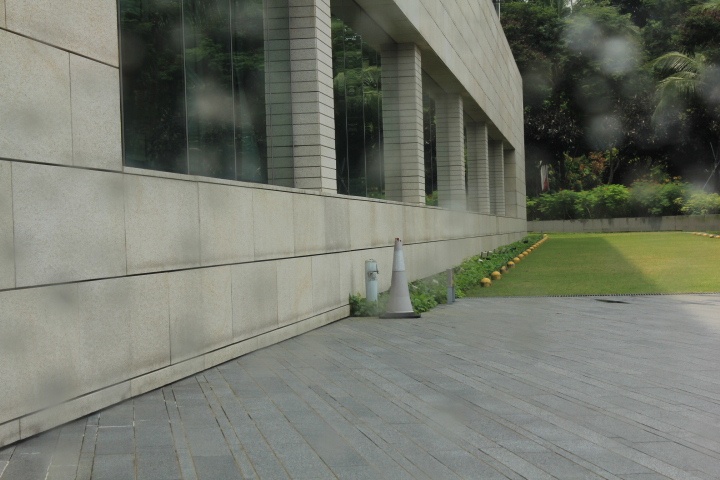

Supplement: S1 Data — (ZIP) [file pone.0301439.s001.zip › test_b/data/178_rain.jpg]

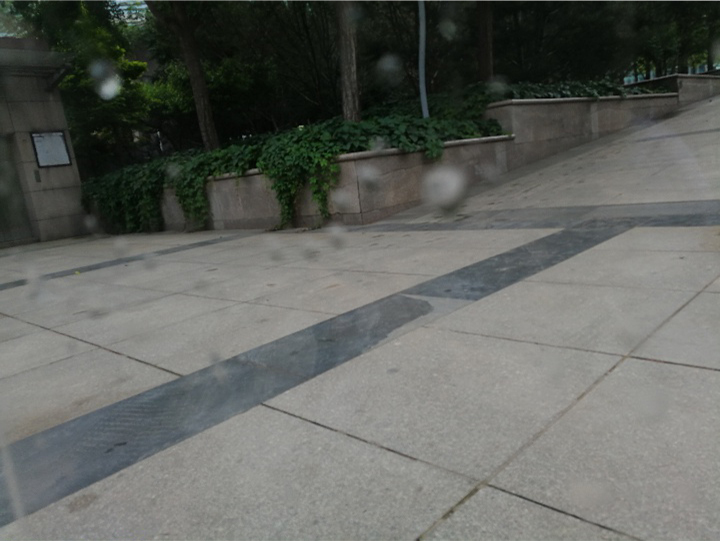

Supplement: S1 Data — (ZIP) [file pone.0301439.s001.zip › test_b/data/179_rain.jpg]

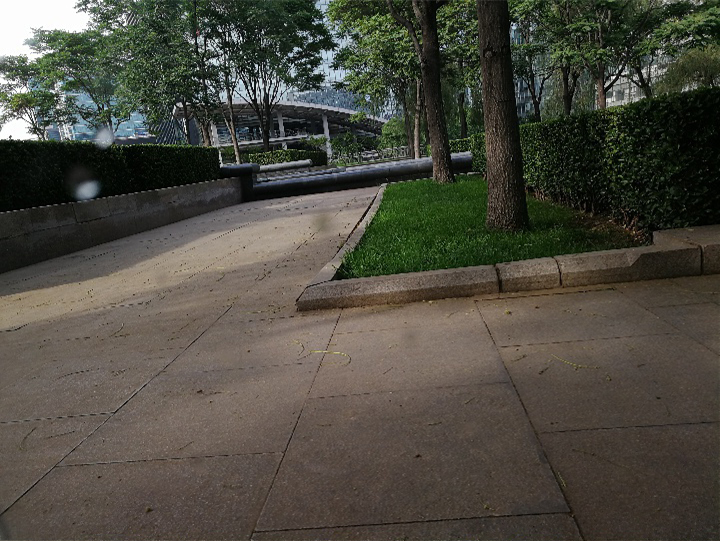

Supplement: S1 Data — (ZIP) [file pone.0301439.s001.zip › test_b/data/17_rain.jpg]

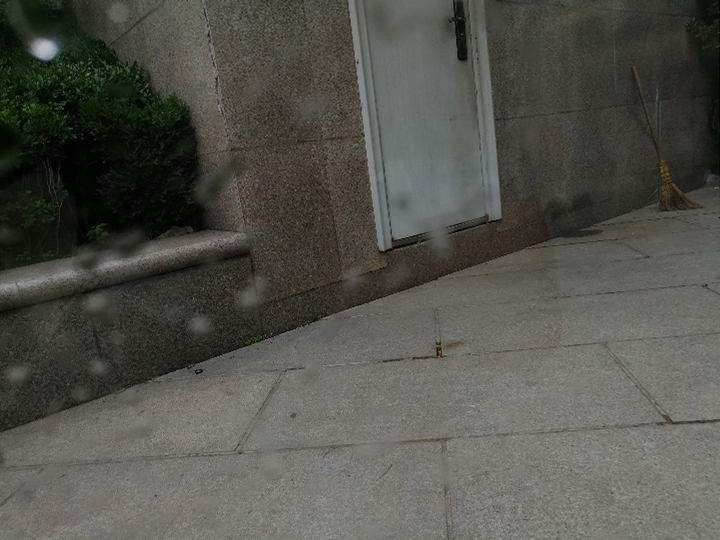

Supplement: S1 Data — (ZIP) [file pone.0301439.s001.zip › test_b/data/180_rain.jpg]

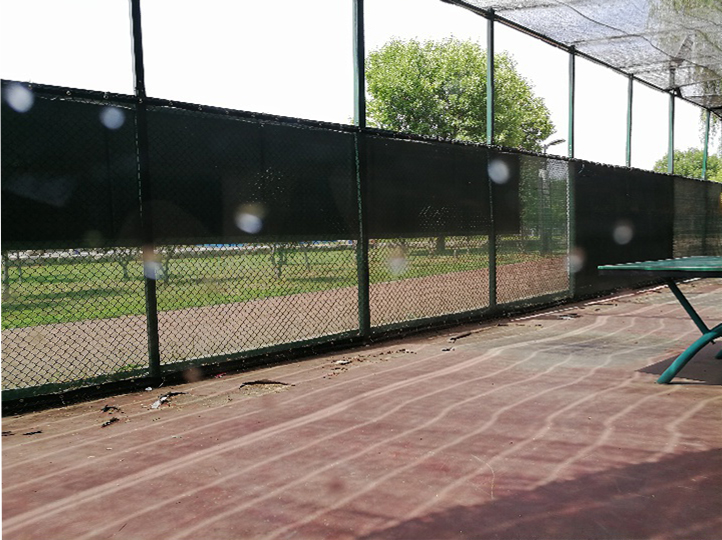

Supplement: S1 Data — (ZIP) [file pone.0301439.s001.zip › test_b/data/181_rain.jpg]

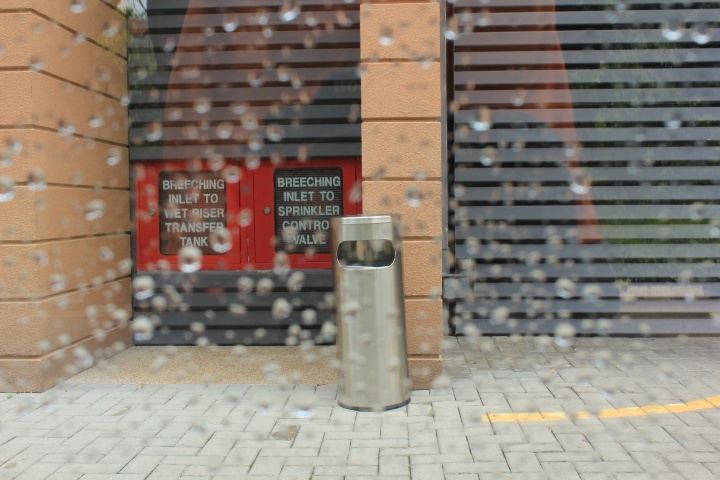

Supplement: S1 Data — (ZIP) [file pone.0301439.s001.zip › test_b/data/182_rain.jpg]

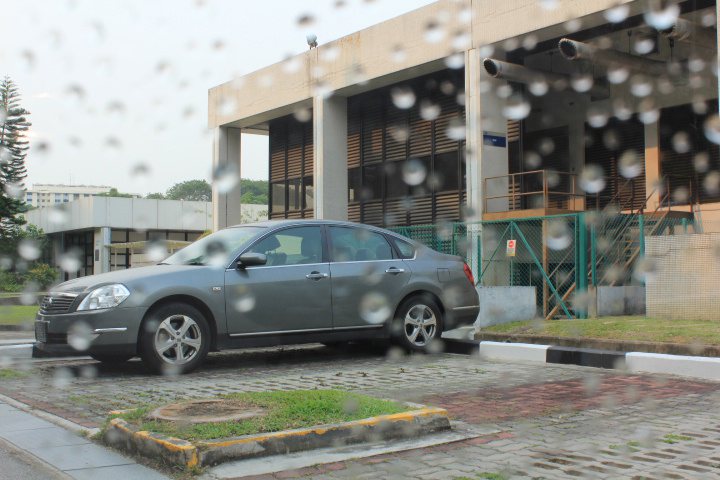

Supplement: S1 Data — (ZIP) [file pone.0301439.s001.zip › test_b/data/183_rain.jpg]

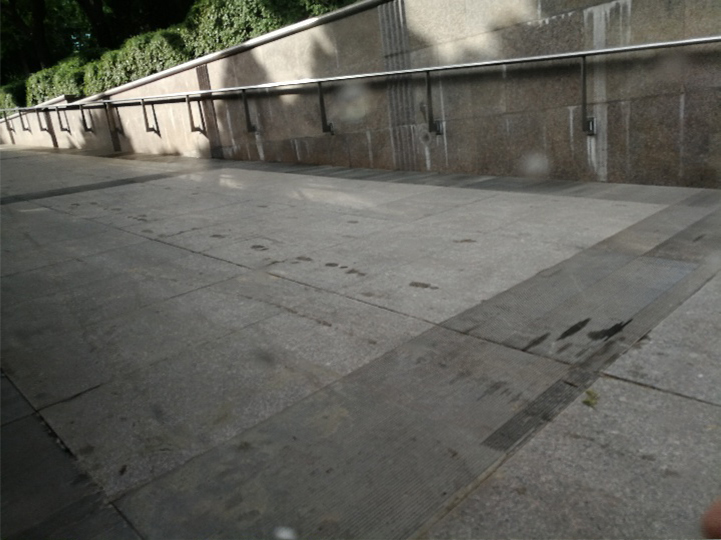

Supplement: S1 Data — (ZIP) [file pone.0301439.s001.zip › test_b/data/184_rain.jpg]

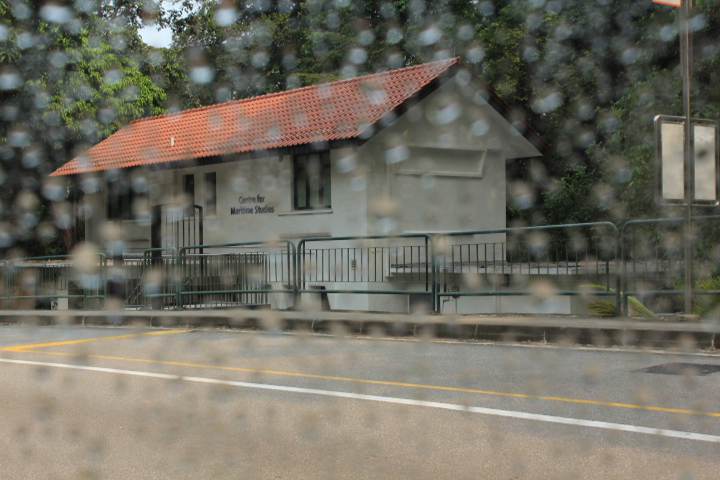

Supplement: S1 Data — (ZIP) [file pone.0301439.s001.zip › test_b/data/185_rain.jpg]

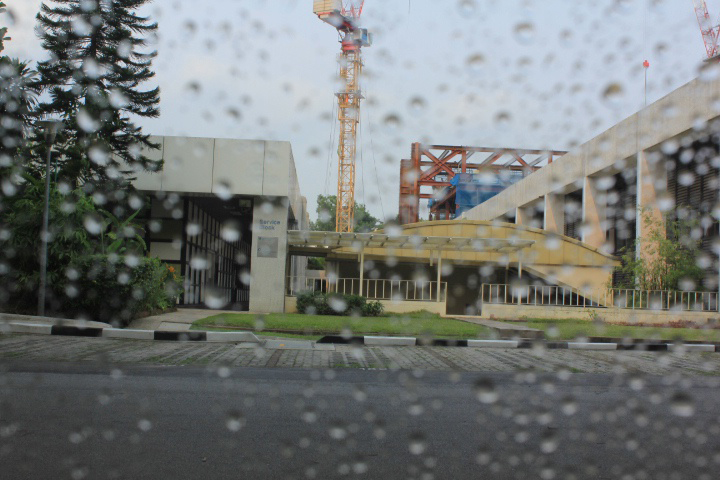

Supplement: S1 Data — (ZIP) [file pone.0301439.s001.zip › test_b/data/186_rain.jpg]

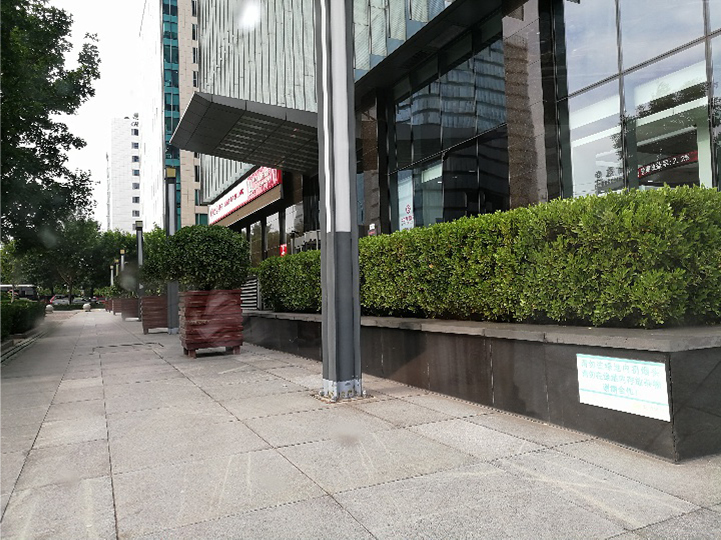

Supplement: S1 Data — (ZIP) [file pone.0301439.s001.zip › test_b/data/187_rain.jpg]

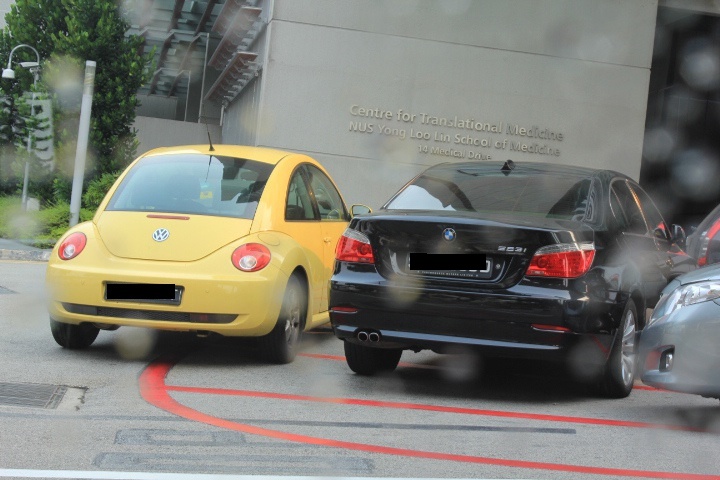

Supplement: S1 Data — (ZIP) [file pone.0301439.s001.zip › test_b/data/188_rain.jpg]

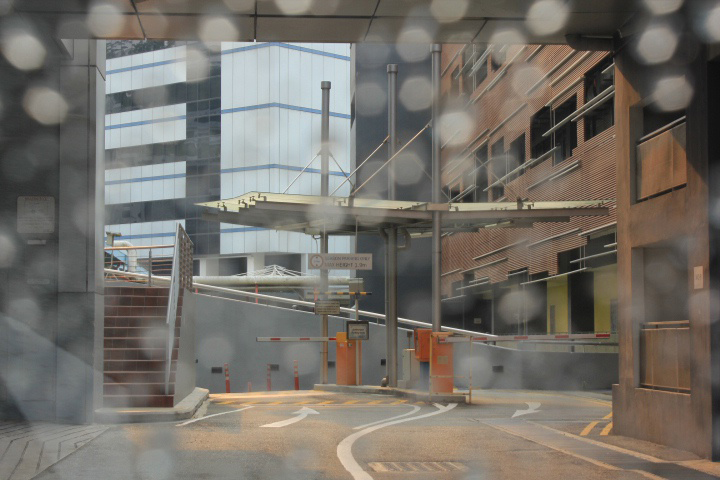

Supplement: S1 Data — (ZIP) [file pone.0301439.s001.zip › test_b/data/189_rain.jpg]

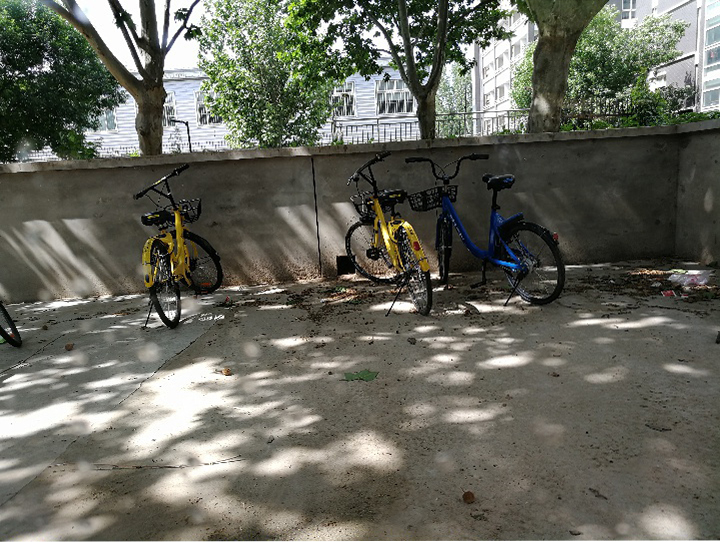

Supplement: S1 Data — (ZIP) [file pone.0301439.s001.zip › test_b/data/18_rain.jpg]

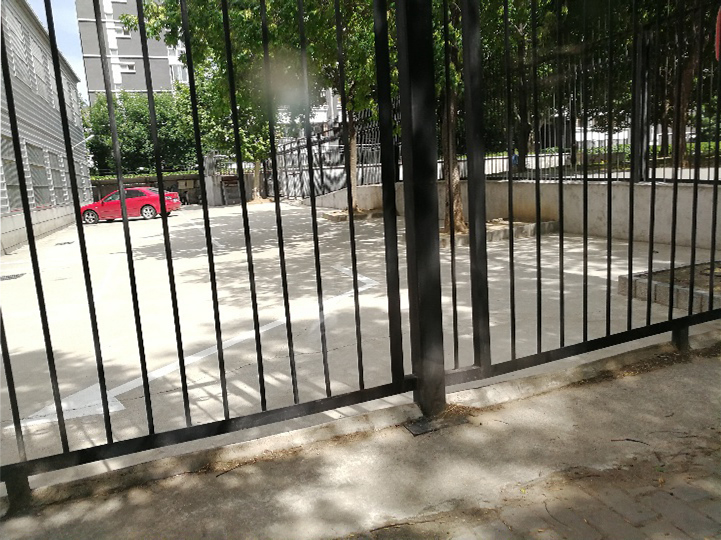

Supplement: S1 Data — (ZIP) [file pone.0301439.s001.zip › test_b/data/190_rain.jpg]

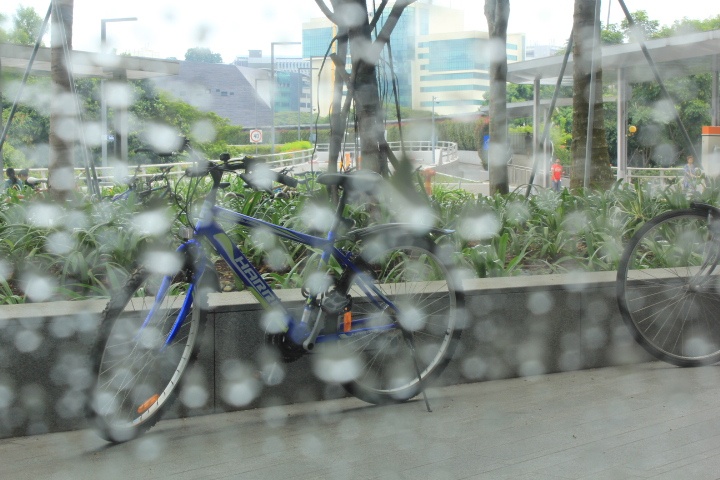

Supplement: S1 Data — (ZIP) [file pone.0301439.s001.zip › test_b/data/191_rain.jpg]

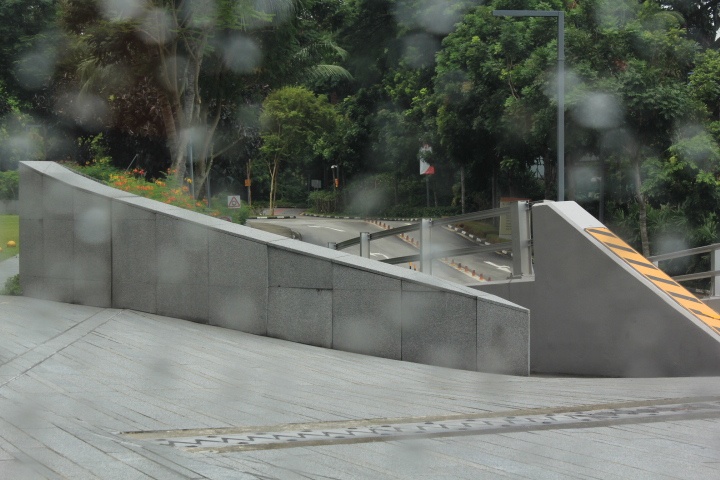

Supplement: S1 Data — (ZIP) [file pone.0301439.s001.zip › test_b/data/192_rain.jpg]

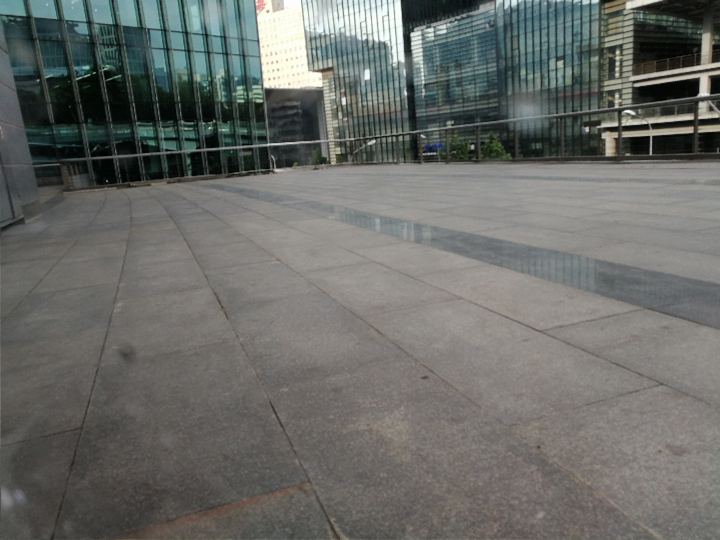

Supplement: S1 Data — (ZIP) [file pone.0301439.s001.zip › test_b/data/193_rain.jpg]

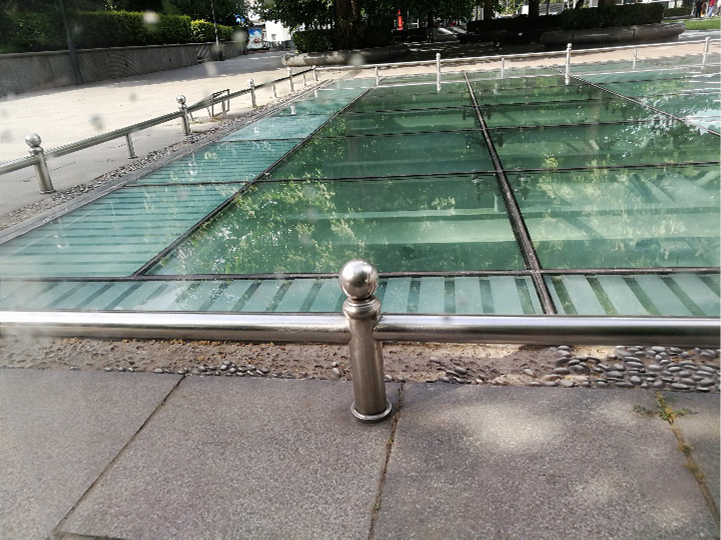

Supplement: S1 Data — (ZIP) [file pone.0301439.s001.zip › test_b/data/194_rain.jpg]

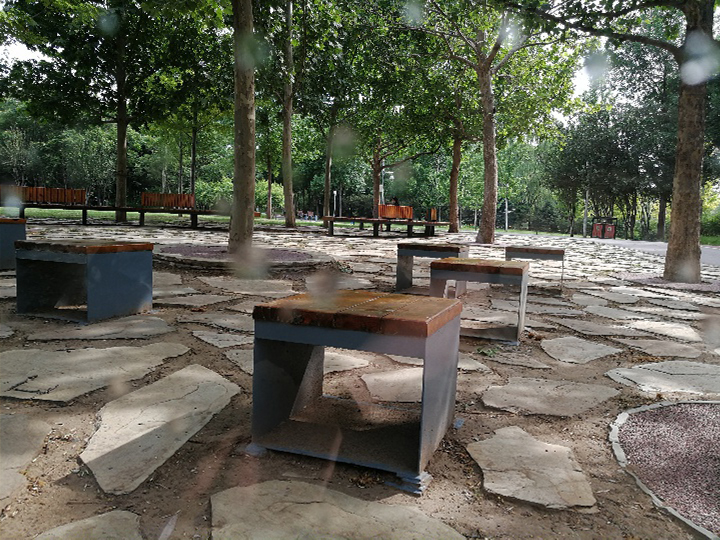

Supplement: S1 Data — (ZIP) [file pone.0301439.s001.zip › test_b/data/195_rain.jpg]

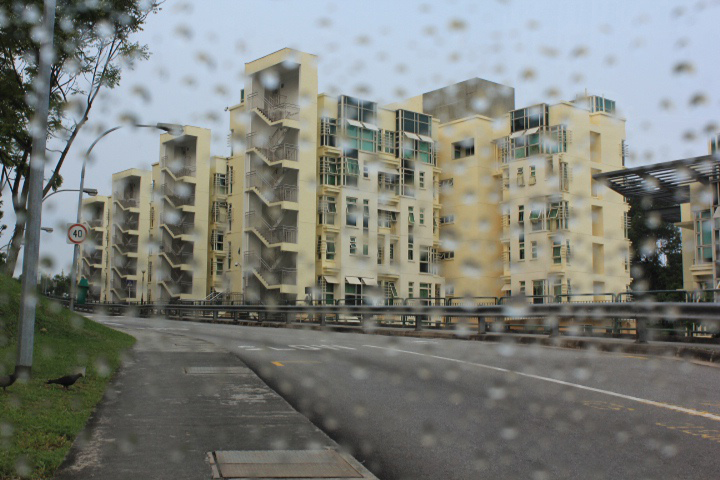

Supplement: S1 Data — (ZIP) [file pone.0301439.s001.zip › test_b/data/196_rain.jpg]

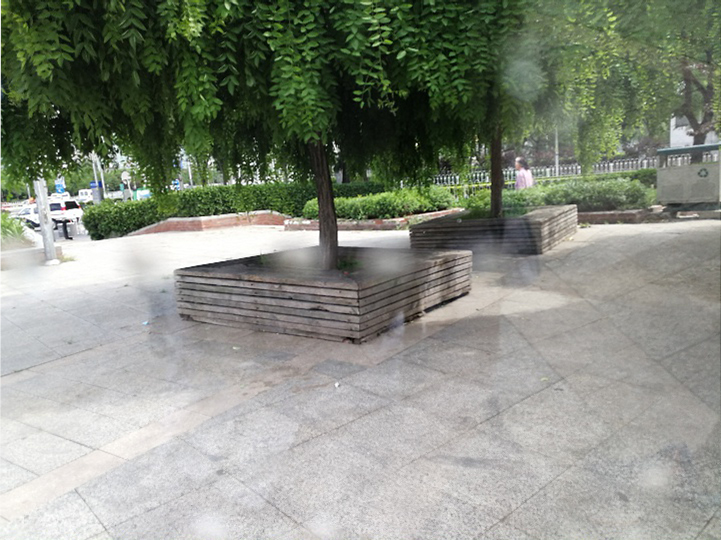

Supplement: S1 Data — (ZIP) [file pone.0301439.s001.zip › test_b/data/197_rain.jpg]

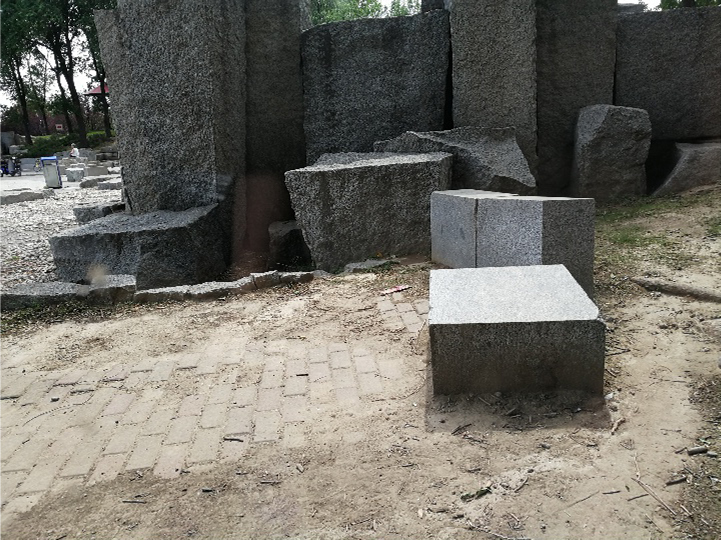

Supplement: S1 Data — (ZIP) [file pone.0301439.s001.zip › test_b/data/198_rain.jpg]

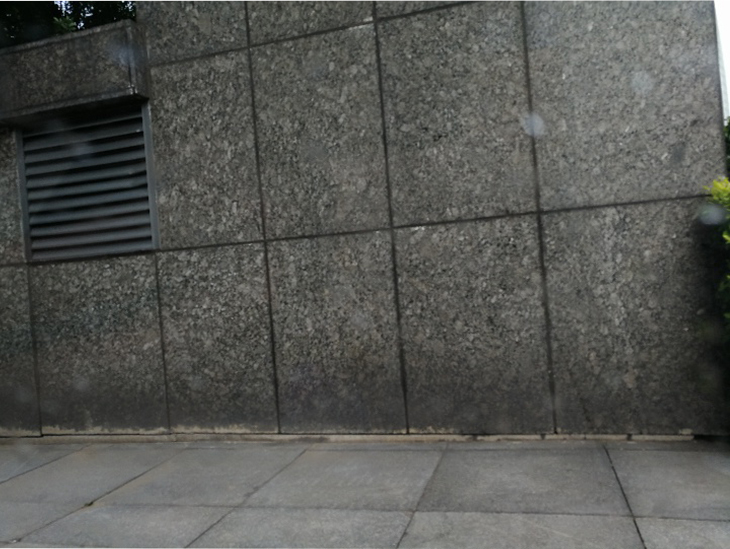

Supplement: S1 Data — (ZIP) [file pone.0301439.s001.zip › test_b/data/199_rain.jpg]

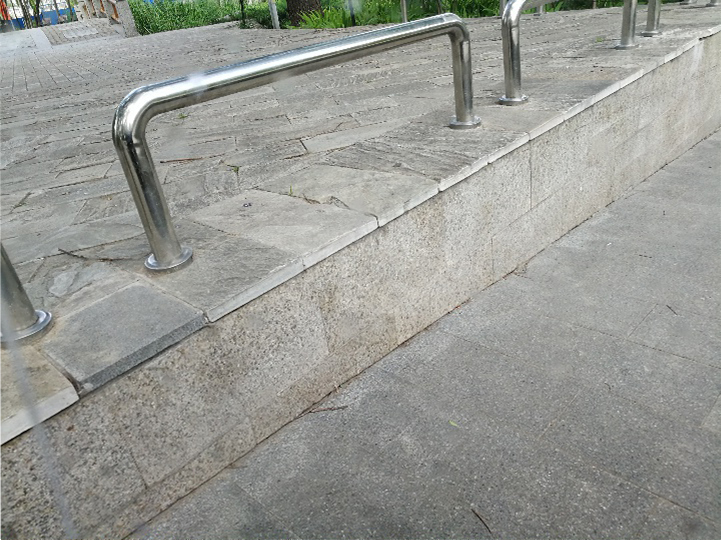

Supplement: S1 Data — (ZIP) [file pone.0301439.s001.zip › test_b/data/19_rain.jpg]

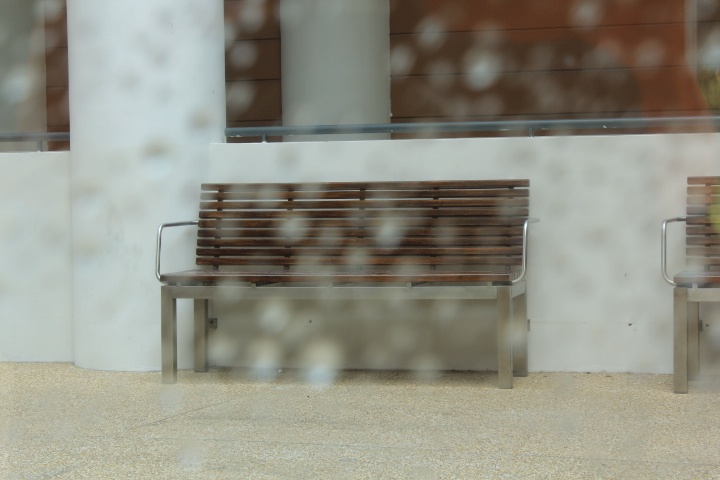

Supplement: S1 Data — (ZIP) [file pone.0301439.s001.zip › test_b/data/1_rain.jpg]

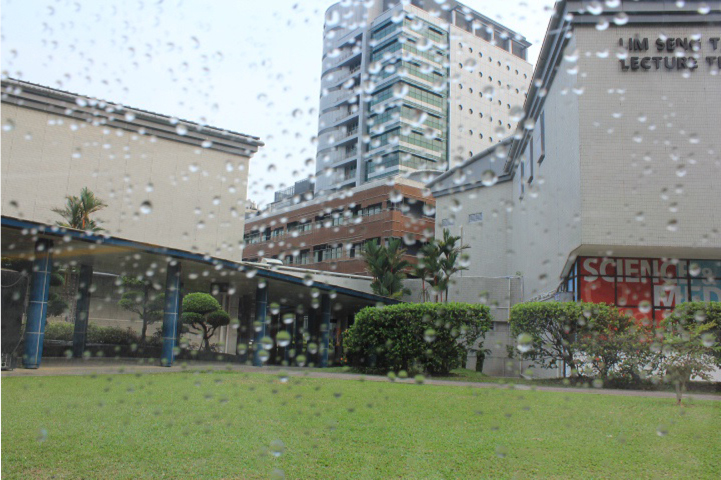

Supplement: S1 Data — (ZIP) [file pone.0301439.s001.zip › test_b/data/200_rain.jpg]

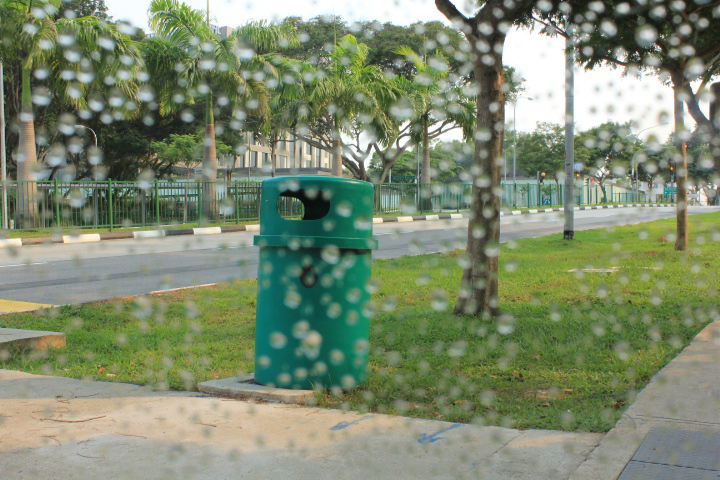

Supplement: S1 Data — (ZIP) [file pone.0301439.s001.zip › test_b/data/201_rain.jpg]

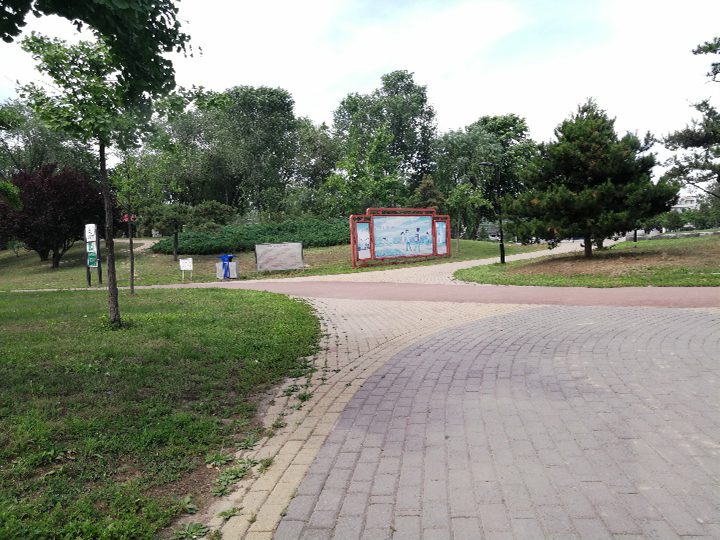

Supplement: S1 Data — (ZIP) [file pone.0301439.s001.zip › test_b/data/202_rain.jpg]

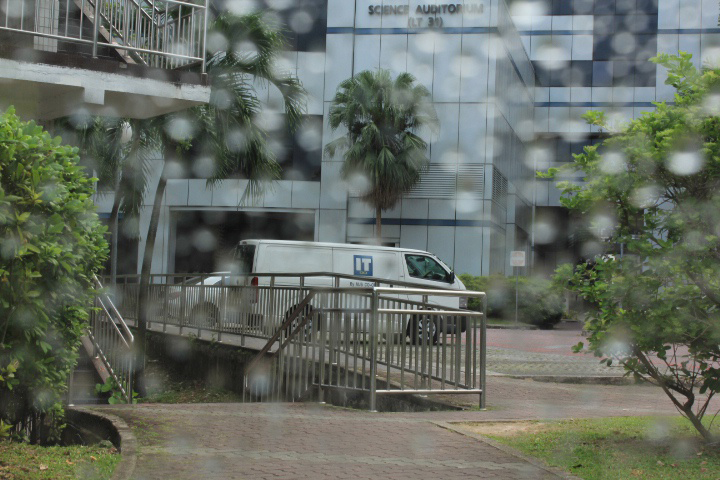

Supplement: S1 Data — (ZIP) [file pone.0301439.s001.zip › test_b/data/203_rain.jpg]

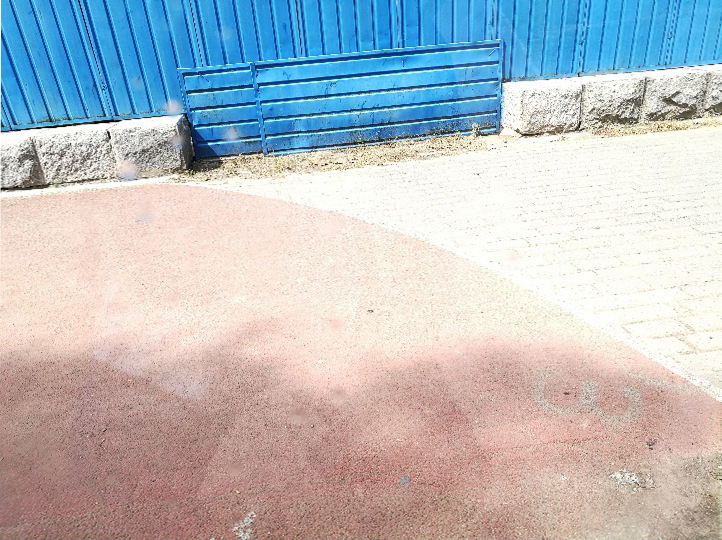

Supplement: S1 Data — (ZIP) [file pone.0301439.s001.zip › test_b/data/204_rain.jpg]

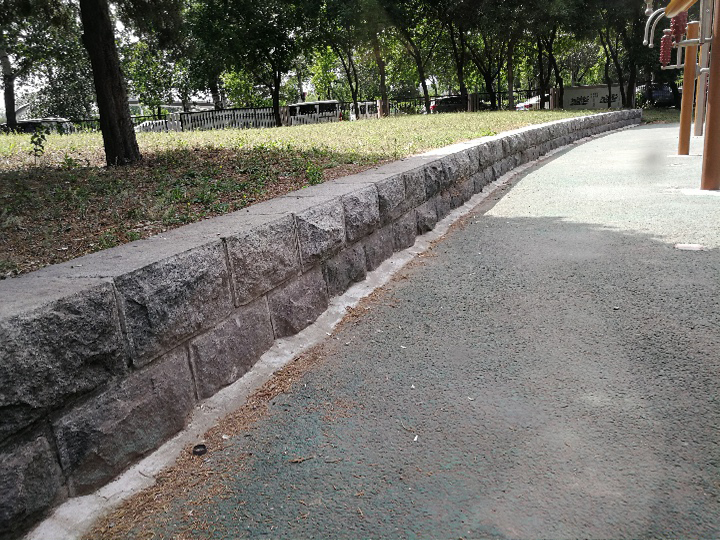

Supplement: S1 Data — (ZIP) [file pone.0301439.s001.zip › test_b/data/205_rain.jpg]

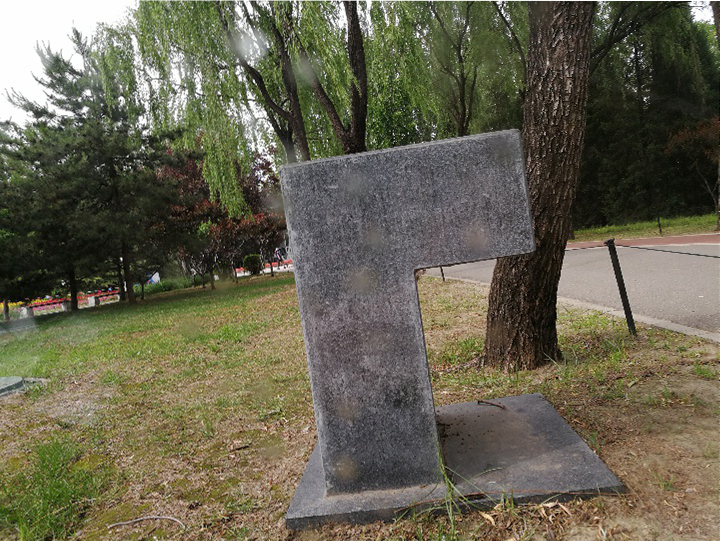

Supplement: S1 Data — (ZIP) [file pone.0301439.s001.zip › test_b/data/206_rain.jpg]

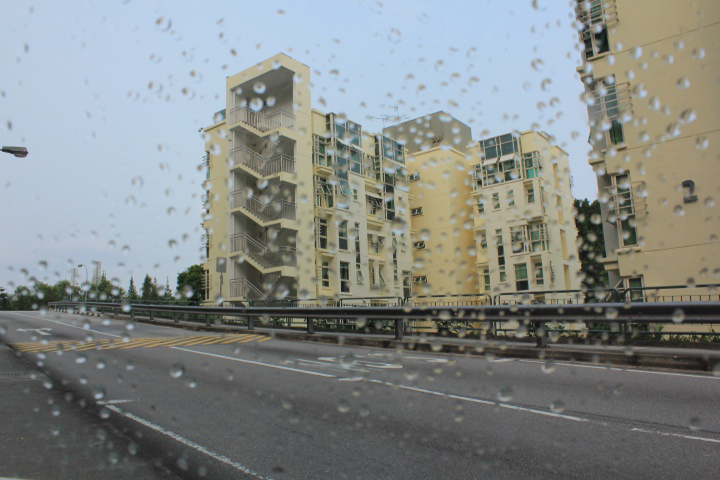

Supplement: S1 Data — (ZIP) [file pone.0301439.s001.zip › test_b/data/207_rain.jpg]

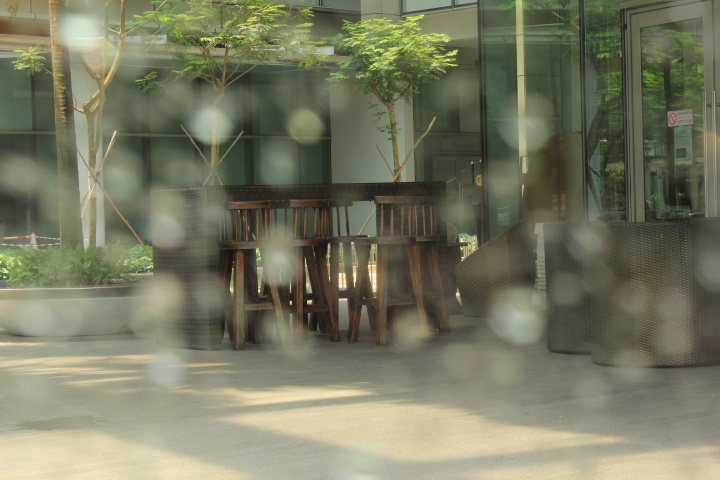

Supplement: S1 Data — (ZIP) [file pone.0301439.s001.zip › test_b/data/208_rain.jpg]

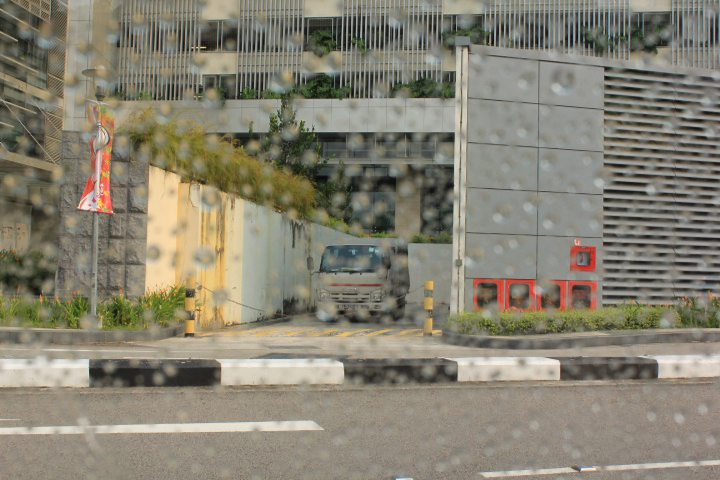

Supplement: S1 Data — (ZIP) [file pone.0301439.s001.zip › test_b/data/209_rain.jpg]

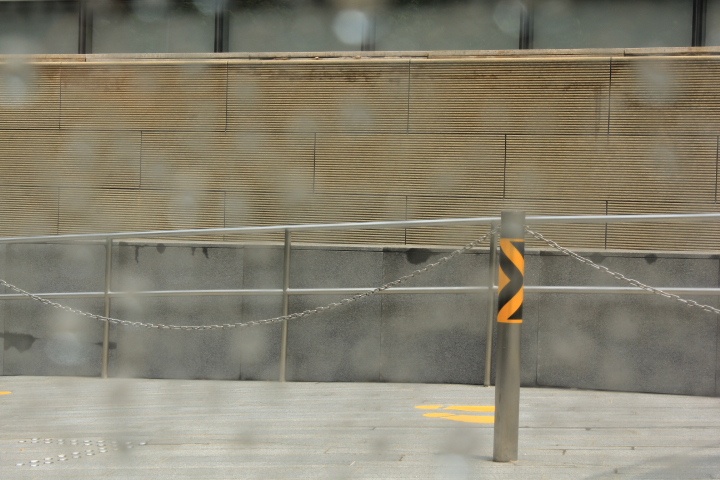

Supplement: S1 Data — (ZIP) [file pone.0301439.s001.zip › test_b/data/20_rain.jpg]

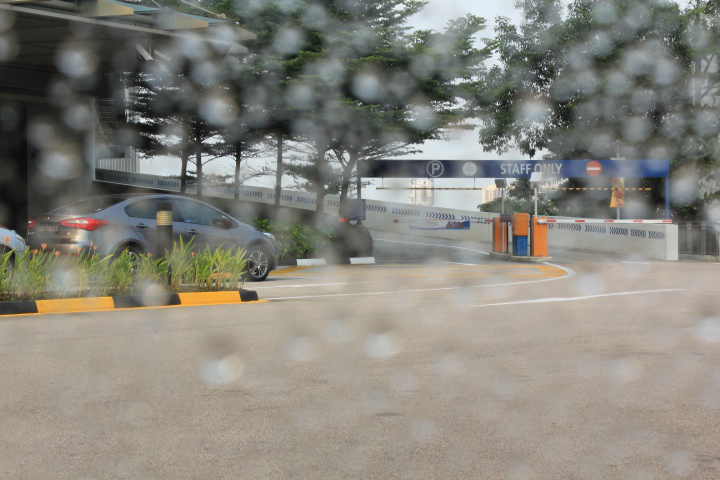

Supplement: S1 Data — (ZIP) [file pone.0301439.s001.zip › test_b/data/210_rain.jpg]

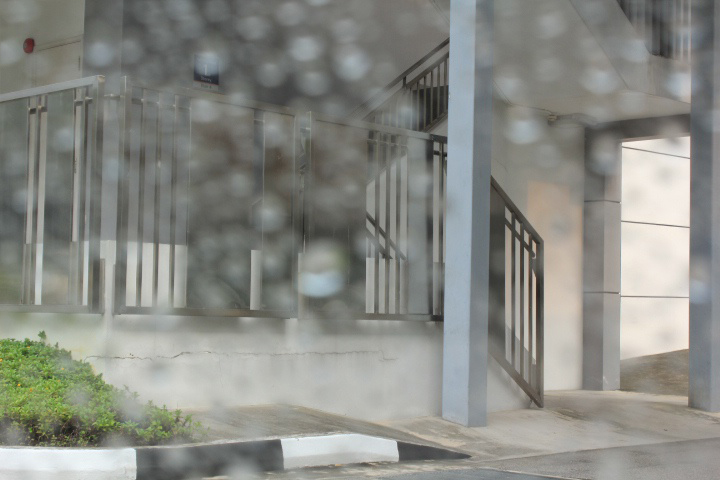

Supplement: S1 Data — (ZIP) [file pone.0301439.s001.zip › test_b/data/211_rain.jpg]

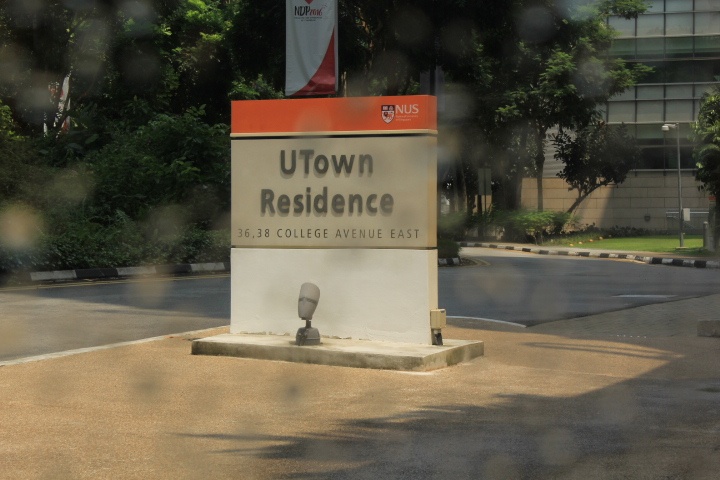

Supplement: S1 Data — (ZIP) [file pone.0301439.s001.zip › test_b/data/212_rain.jpg]

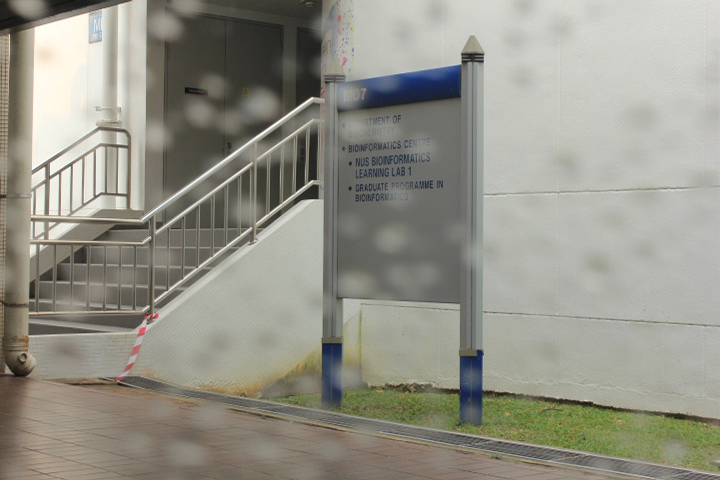

Supplement: S1 Data — (ZIP) [file pone.0301439.s001.zip › test_b/data/213_rain.jpg]

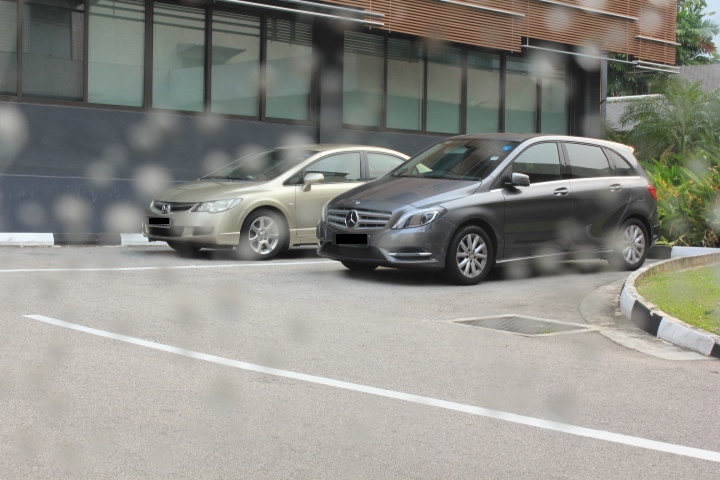

Supplement: S1 Data — (ZIP) [file pone.0301439.s001.zip › test_b/data/214_rain.jpg]

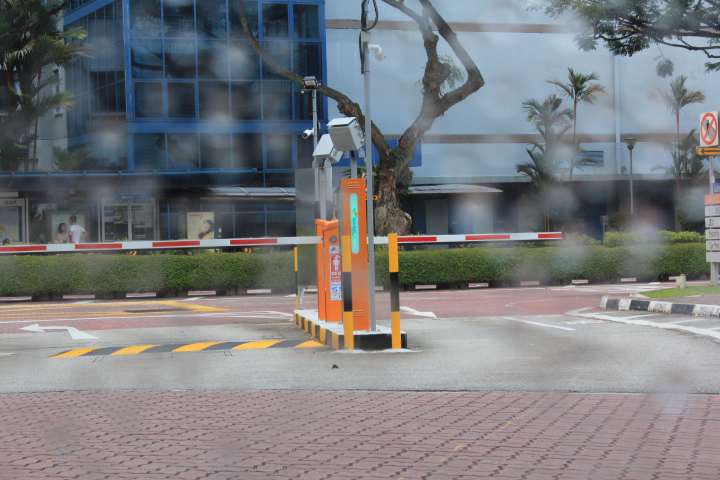

Supplement: S1 Data — (ZIP) [file pone.0301439.s001.zip › test_b/data/215_rain.jpg]

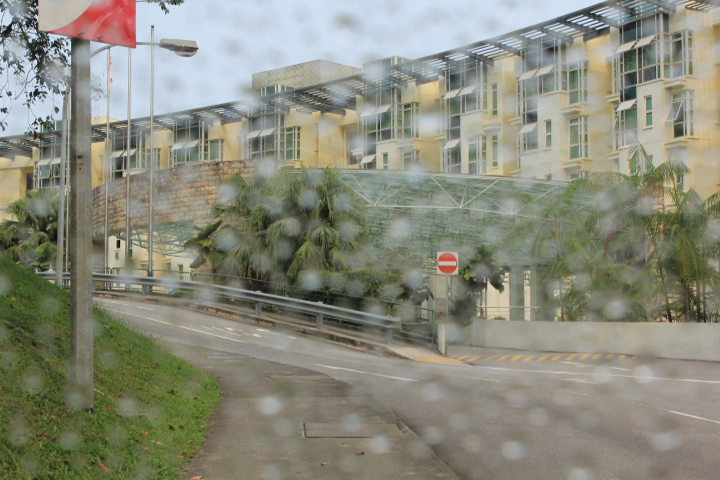

Supplement: S1 Data — (ZIP) [file pone.0301439.s001.zip › test_b/data/216_rain.jpg]

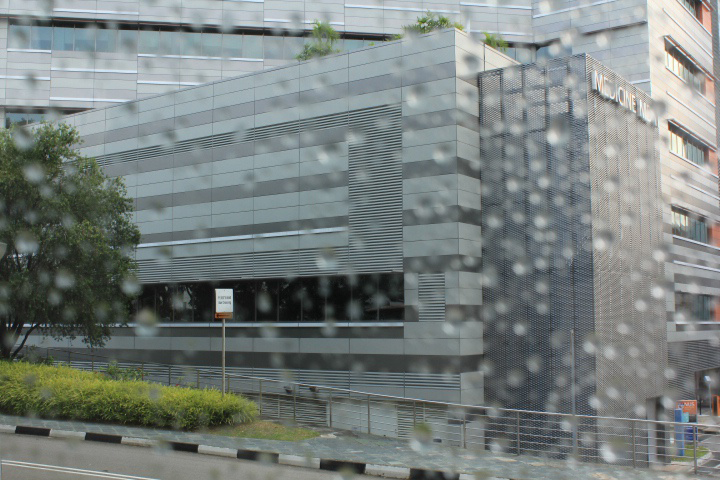

Supplement: S1 Data — (ZIP) [file pone.0301439.s001.zip › test_b/data/217_rain.jpg]

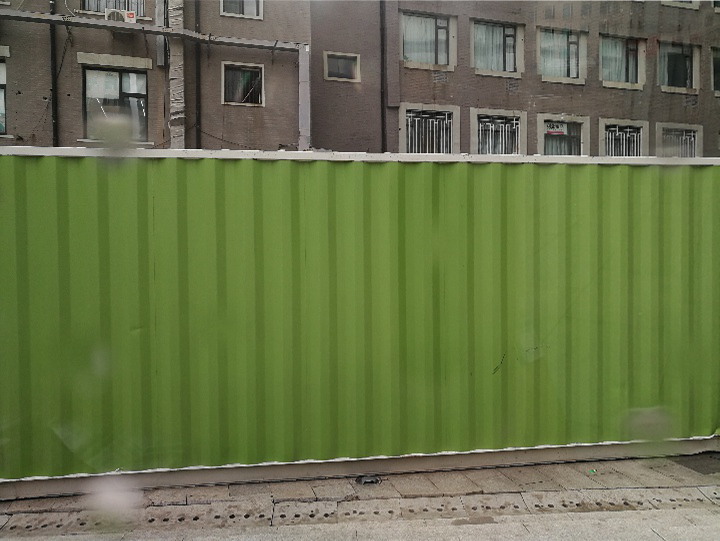

Supplement: S1 Data — (ZIP) [file pone.0301439.s001.zip › test_b/data/218_rain.jpg]

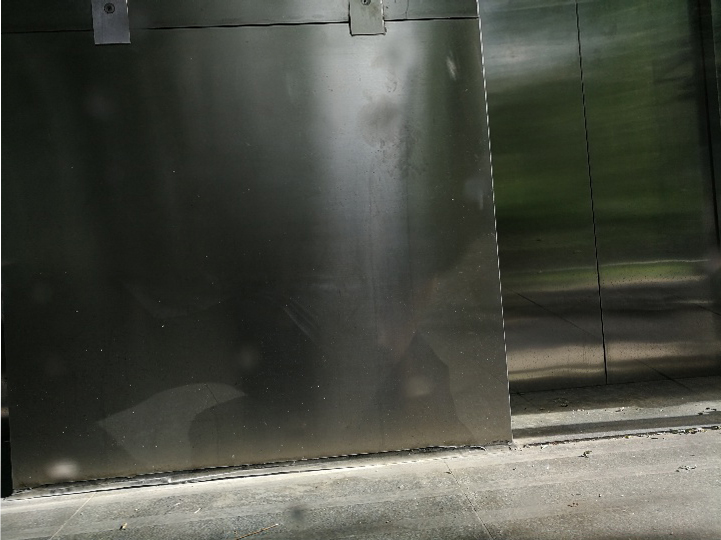

Supplement: S1 Data — (ZIP) [file pone.0301439.s001.zip › test_b/data/219_rain.jpg]

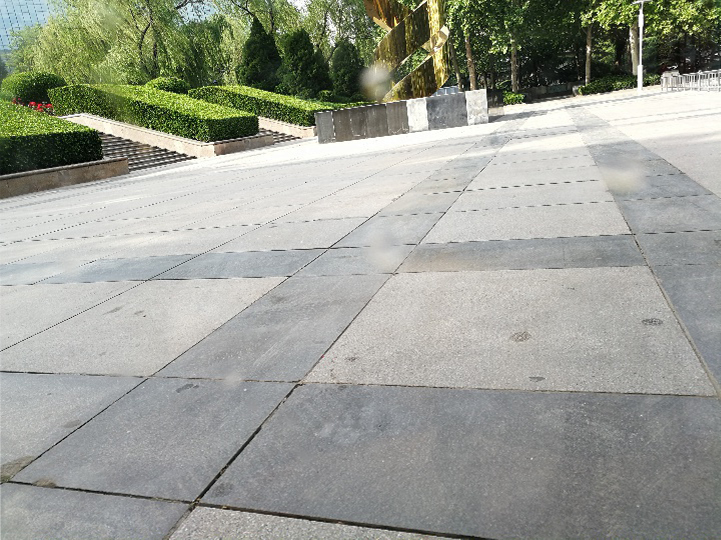

Supplement: S1 Data — (ZIP) [file pone.0301439.s001.zip › test_b/data/21_rain.jpg]

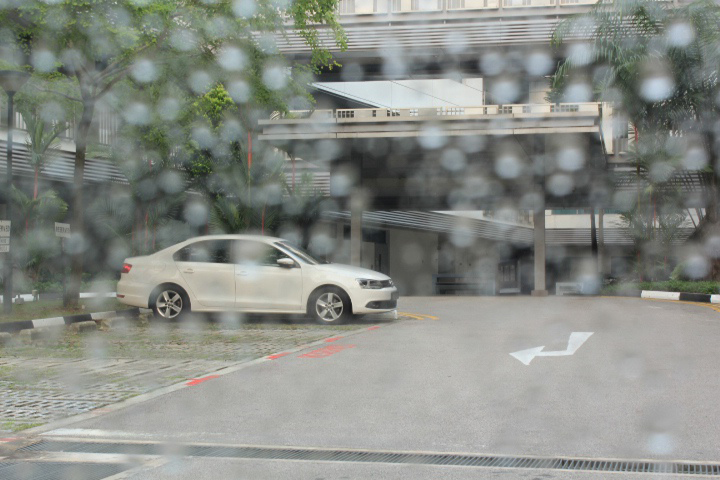

Supplement: S1 Data — (ZIP) [file pone.0301439.s001.zip › test_b/data/220_rain.jpg]

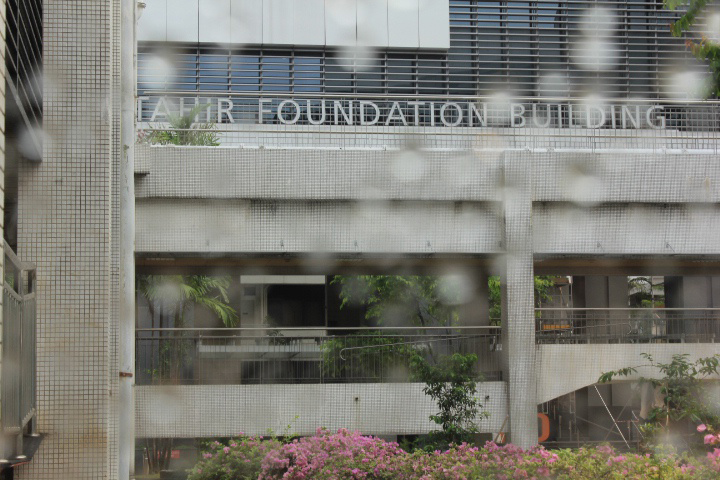

Supplement: S1 Data — (ZIP) [file pone.0301439.s001.zip › test_b/data/221_rain.jpg]

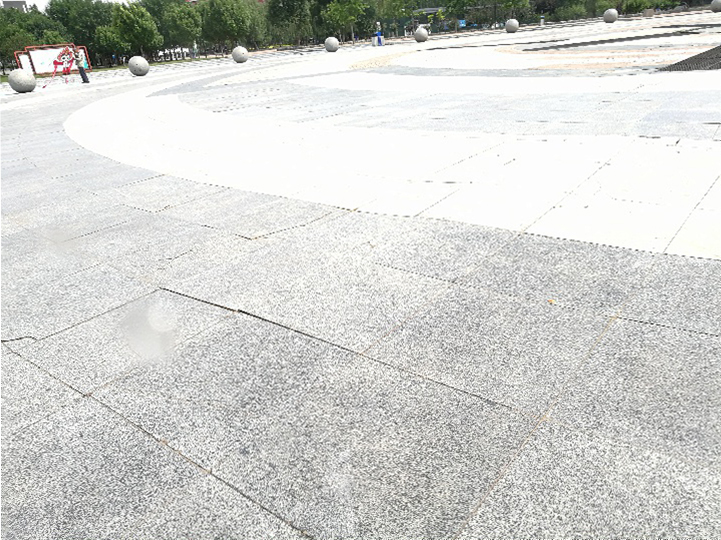

Supplement: S1 Data — (ZIP) [file pone.0301439.s001.zip › test_b/data/222_rain.jpg]

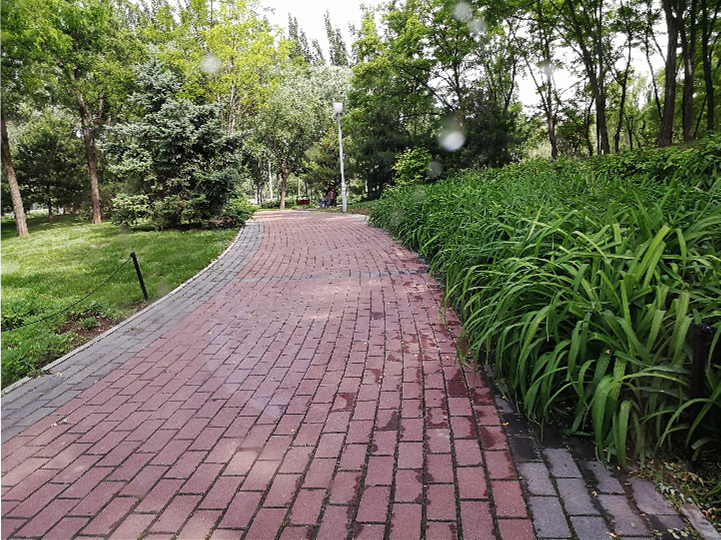

Supplement: S1 Data — (ZIP) [file pone.0301439.s001.zip › test_b/data/223_rain.jpg]

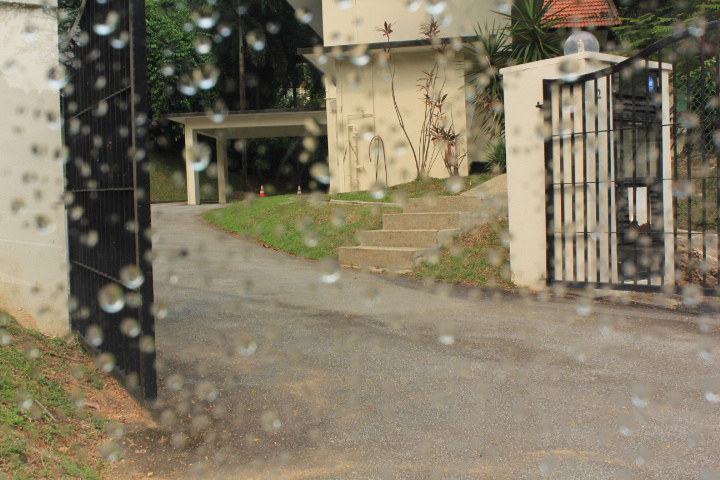

Supplement: S1 Data — (ZIP) [file pone.0301439.s001.zip › test_b/data/224_rain.jpg]

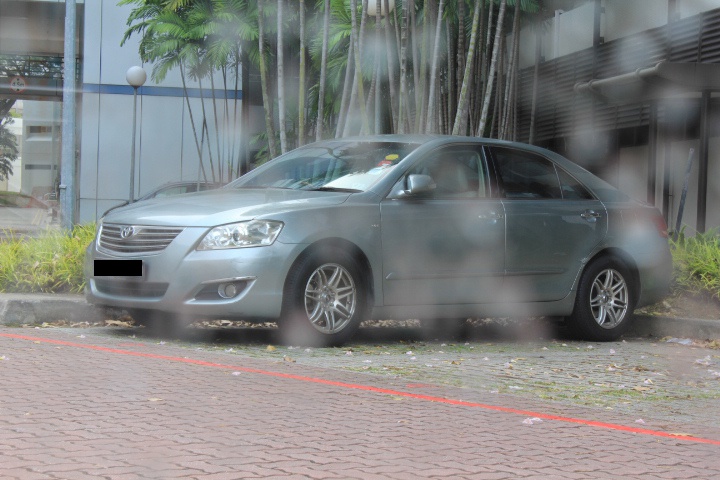

Supplement: S1 Data — (ZIP) [file pone.0301439.s001.zip › test_b/data/225_rain.jpg]

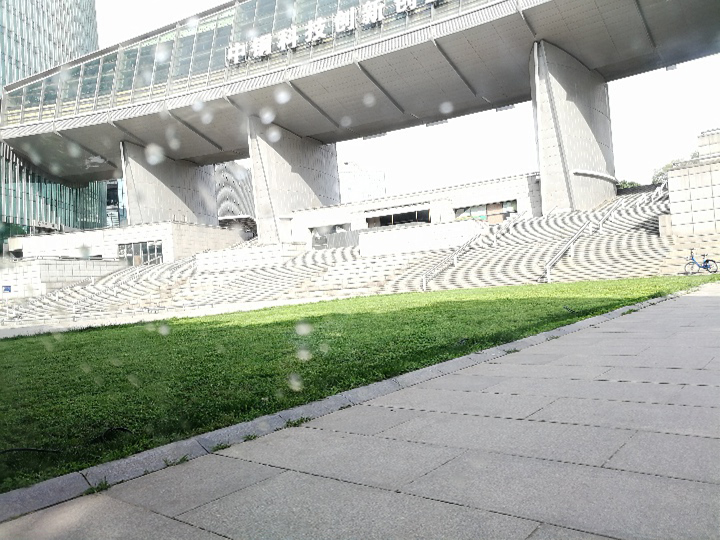

Supplement: S1 Data — (ZIP) [file pone.0301439.s001.zip › test_b/data/226_rain.jpg]

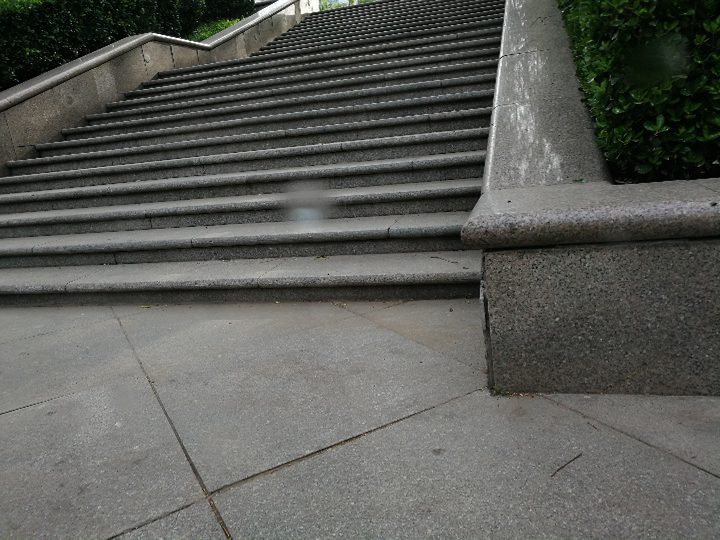

Supplement: S1 Data — (ZIP) [file pone.0301439.s001.zip › test_b/data/227_rain.jpg]
