# Supplementary figures and images for: Frequency-oriented hierarchical fusion network for single image raindrop removal (part 1 of 2)
Source: PLoS One. 2024 May 23;19(5):e0301439. doi: 10.1371/journal.pone.0301439 (PMC11115234; doi:10.1371/journal.pone.0301439)

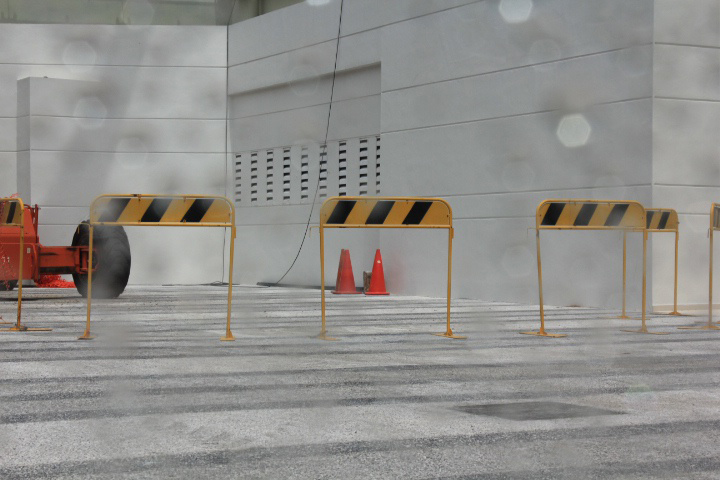

Supplement: S1 Data — (ZIP) [file pone.0301439.s001.zip › test_a/data/0_rain.png]

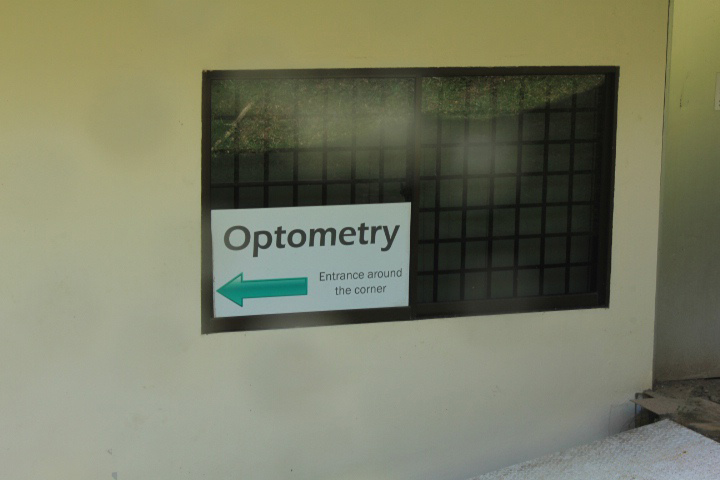

Supplement: S1 Data — (ZIP) [file pone.0301439.s001.zip › test_a/data/10_rain.png]

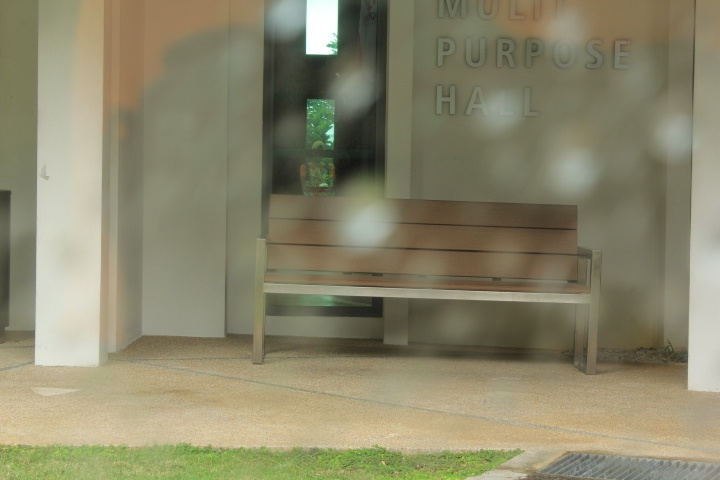

Supplement: S1 Data — (ZIP) [file pone.0301439.s001.zip › test_a/data/11_rain.png]

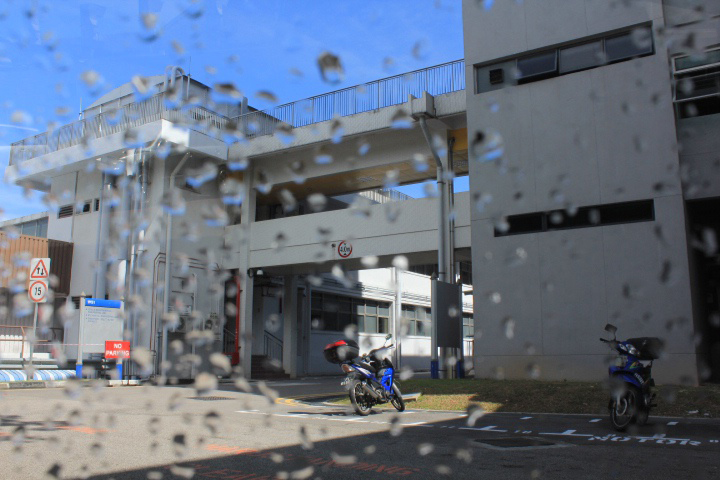

Supplement: S1 Data — (ZIP) [file pone.0301439.s001.zip › test_a/data/12_rain.png]

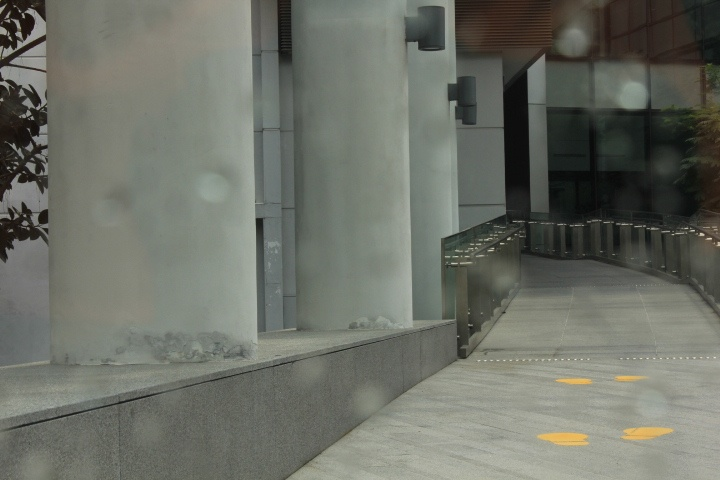

Supplement: S1 Data — (ZIP) [file pone.0301439.s001.zip › test_a/data/13_rain.png]

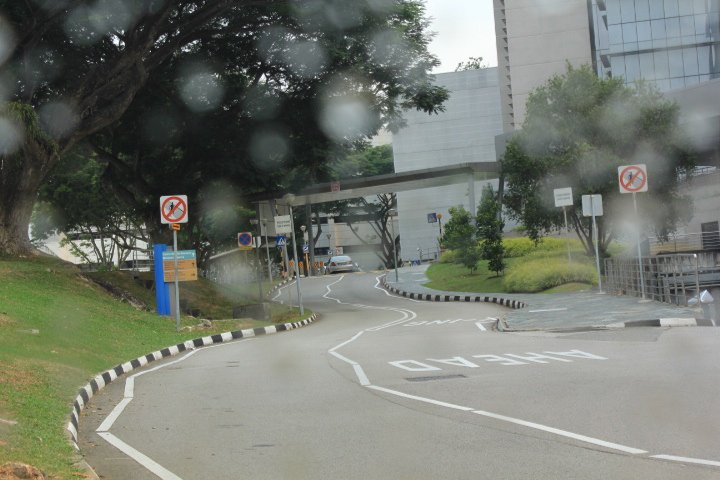

Supplement: S1 Data — (ZIP) [file pone.0301439.s001.zip › test_a/data/14_rain.png]

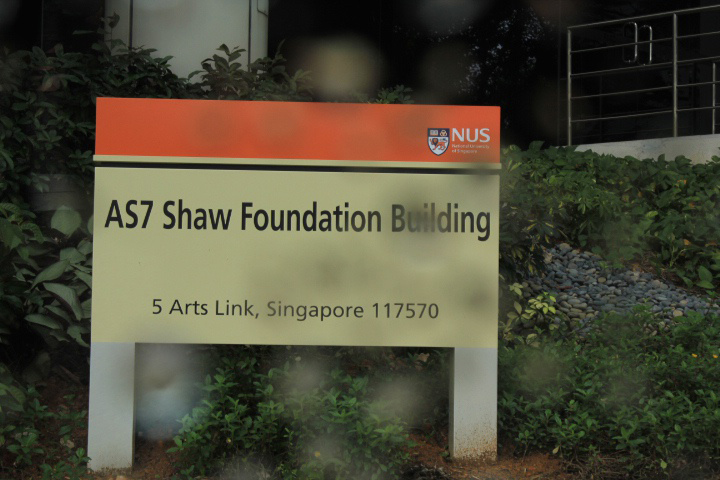

Supplement: S1 Data — (ZIP) [file pone.0301439.s001.zip › test_a/data/15_rain.png]

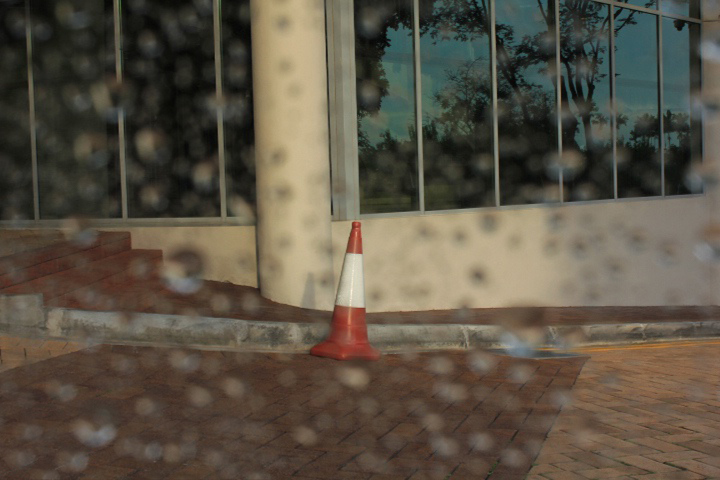

Supplement: S1 Data — (ZIP) [file pone.0301439.s001.zip › test_a/data/16_rain.png]

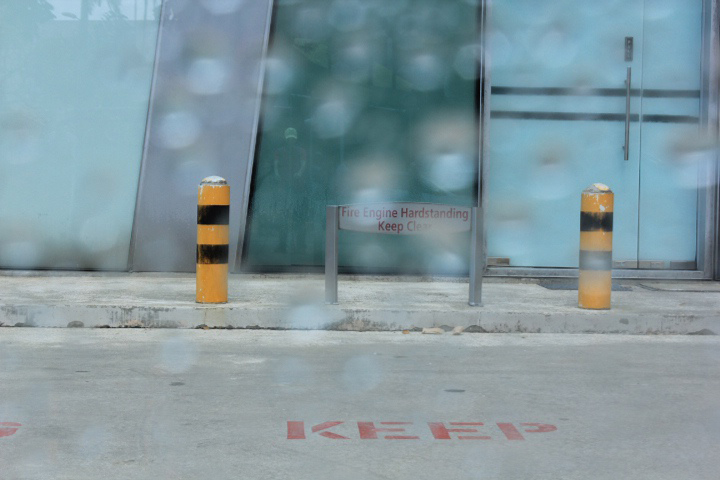

Supplement: S1 Data — (ZIP) [file pone.0301439.s001.zip › test_a/data/17_rain.png]

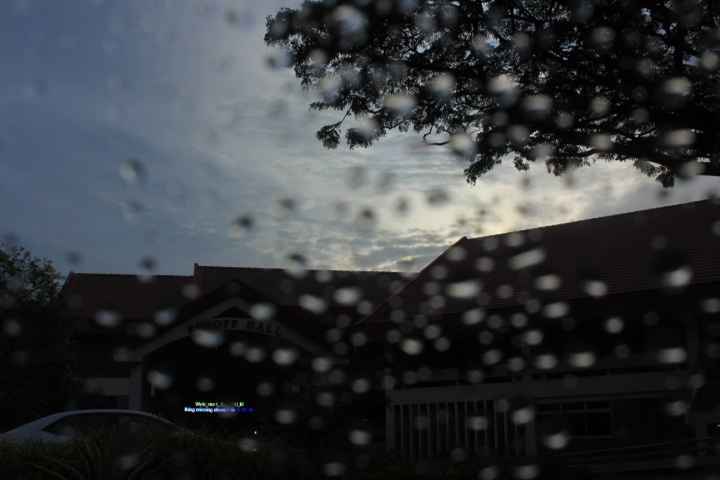

Supplement: S1 Data — (ZIP) [file pone.0301439.s001.zip › test_a/data/18_rain.png]

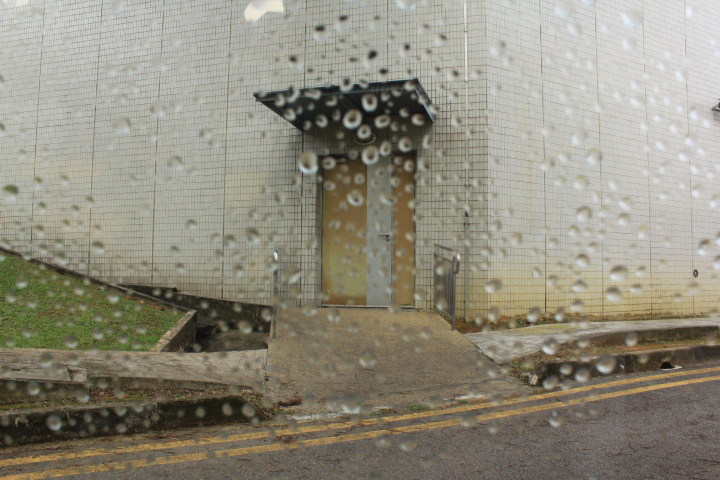

Supplement: S1 Data — (ZIP) [file pone.0301439.s001.zip › test_a/data/19_rain.png]

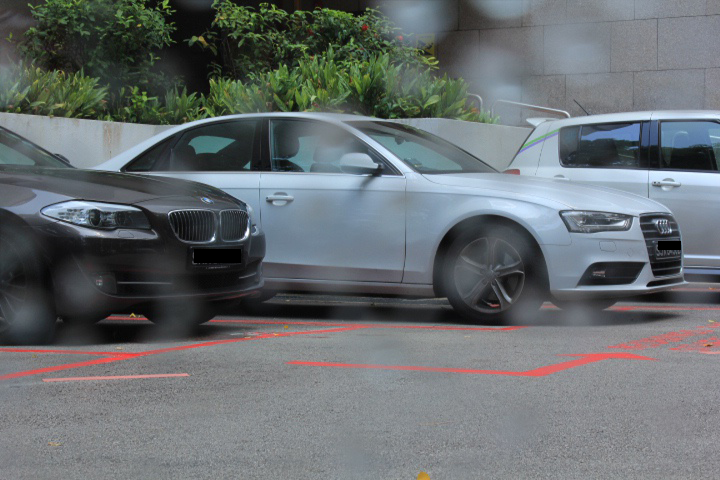

Supplement: S1 Data — (ZIP) [file pone.0301439.s001.zip › test_a/data/1_rain.png]

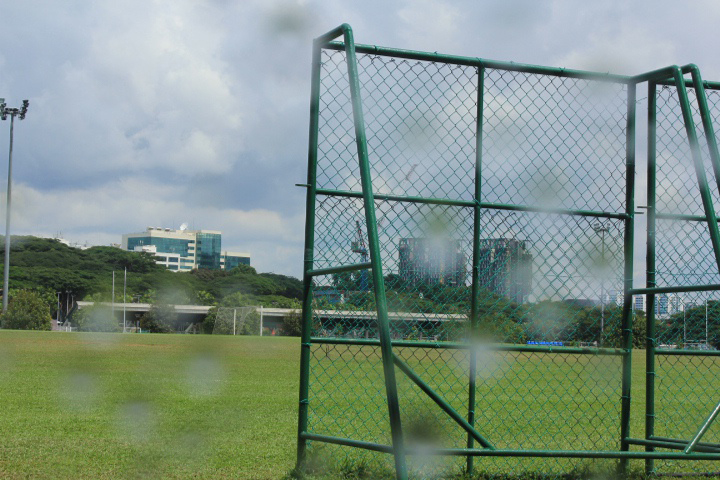

Supplement: S1 Data — (ZIP) [file pone.0301439.s001.zip › test_a/data/20_rain.png]

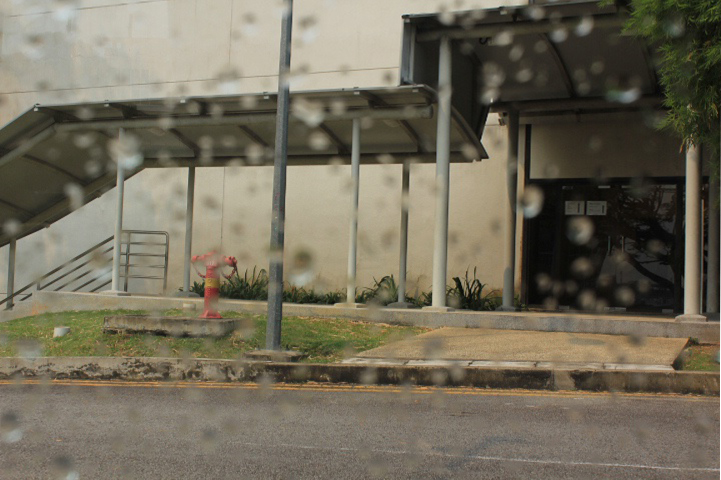

Supplement: S1 Data — (ZIP) [file pone.0301439.s001.zip › test_a/data/21_rain.png]

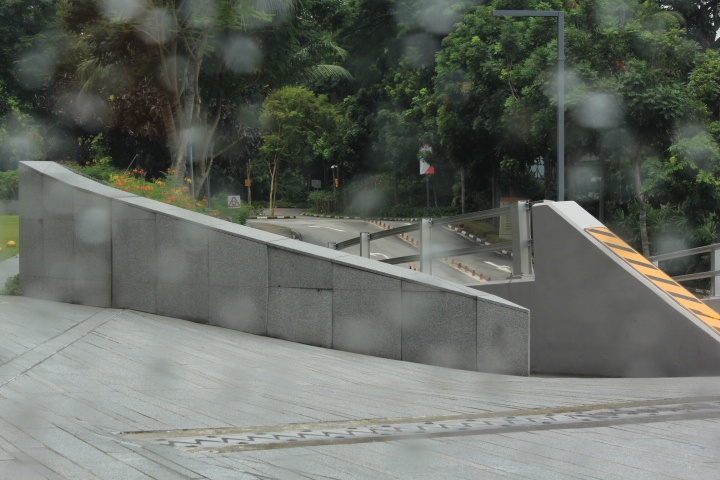

Supplement: S1 Data — (ZIP) [file pone.0301439.s001.zip › test_a/data/22_rain.png]

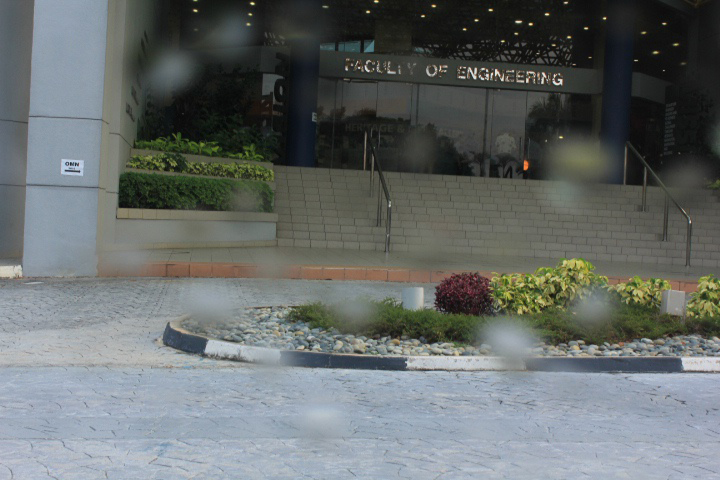

Supplement: S1 Data — (ZIP) [file pone.0301439.s001.zip › test_a/data/23_rain.png]

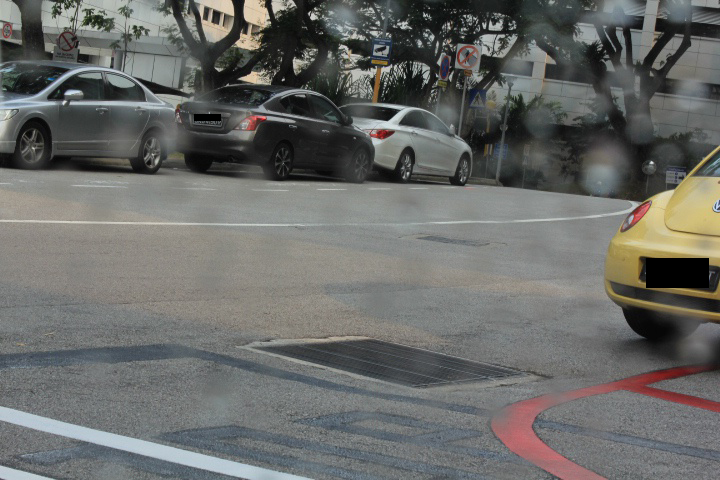

Supplement: S1 Data — (ZIP) [file pone.0301439.s001.zip › test_a/data/24_rain.png]

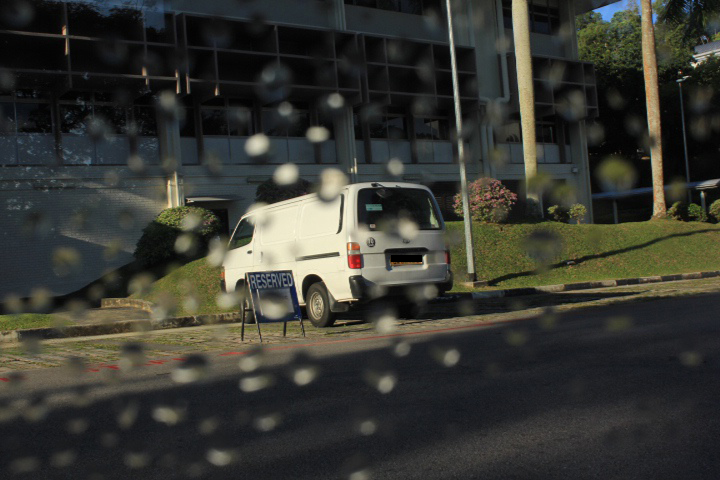

Supplement: S1 Data — (ZIP) [file pone.0301439.s001.zip › test_a/data/25_rain.png]

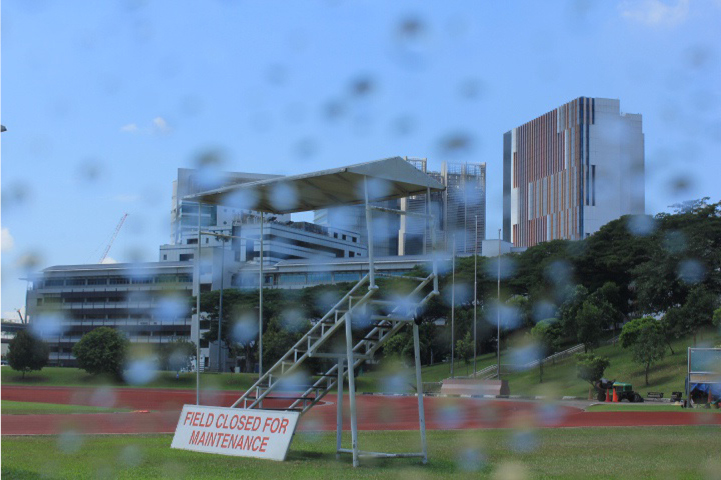

Supplement: S1 Data — (ZIP) [file pone.0301439.s001.zip › test_a/data/26_rain.png]

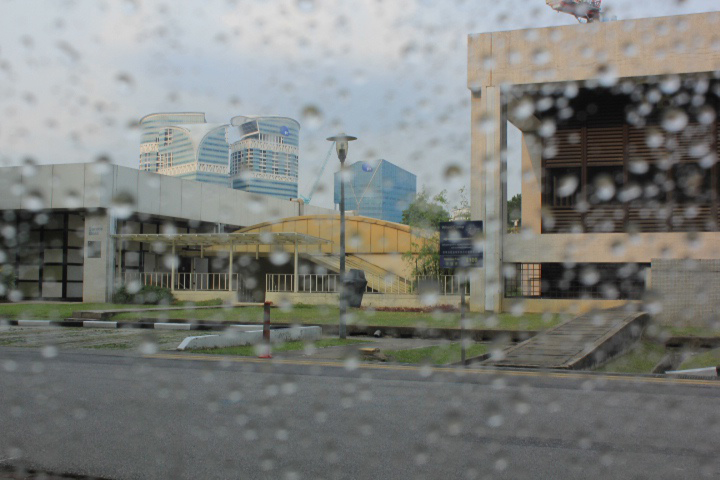

Supplement: S1 Data — (ZIP) [file pone.0301439.s001.zip › test_a/data/27_rain.png]

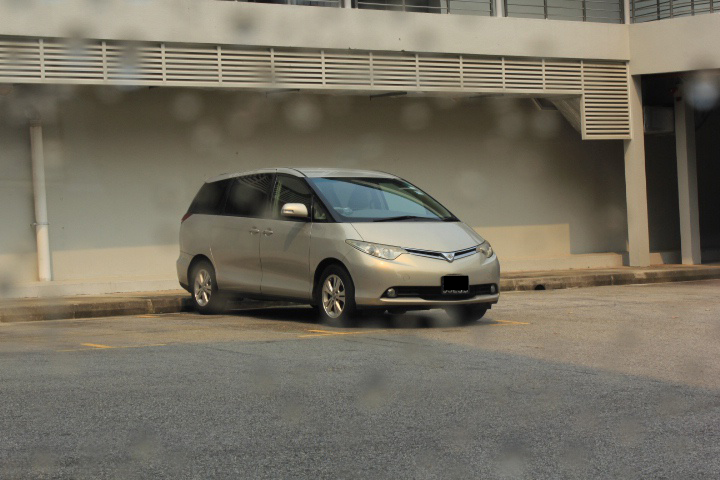

Supplement: S1 Data — (ZIP) [file pone.0301439.s001.zip › test_a/data/28_rain.png]

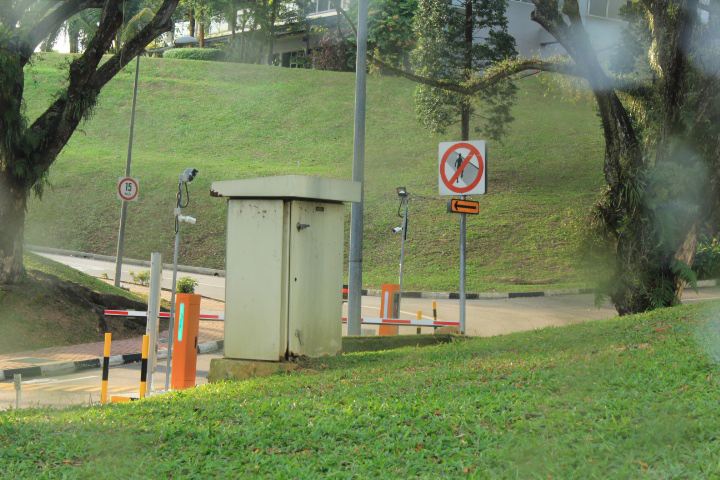

Supplement: S1 Data — (ZIP) [file pone.0301439.s001.zip › test_a/data/29_rain.png]

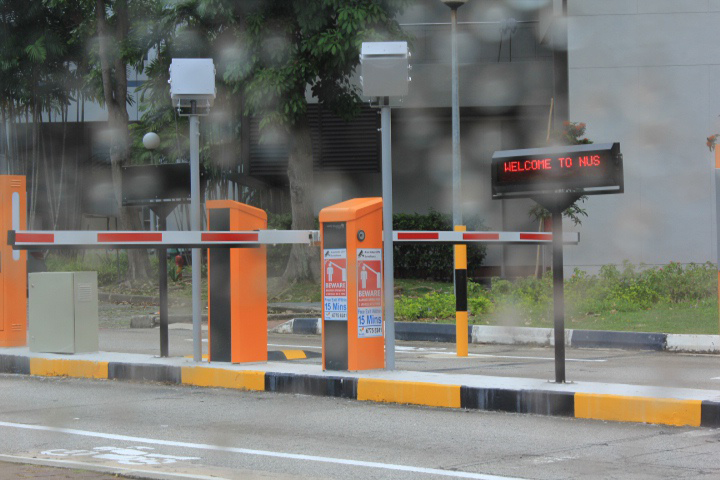

Supplement: S1 Data — (ZIP) [file pone.0301439.s001.zip › test_a/data/2_rain.png]

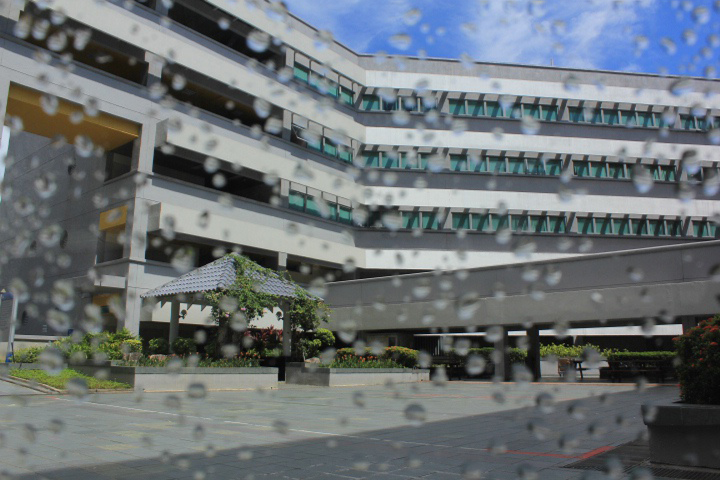

Supplement: S1 Data — (ZIP) [file pone.0301439.s001.zip › test_a/data/30_rain.png]

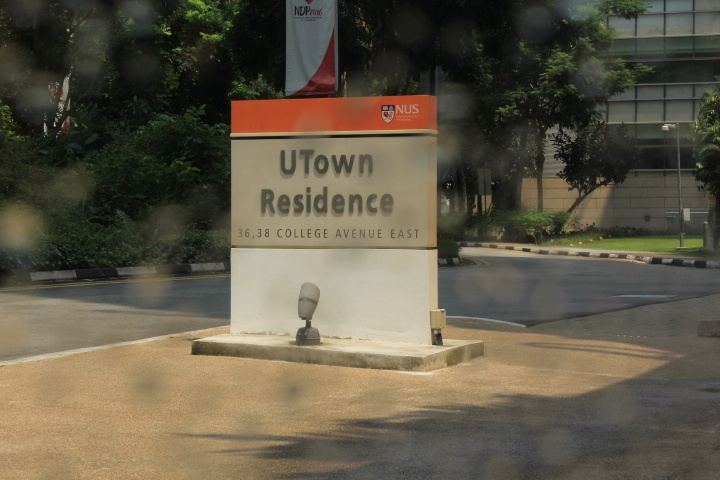

Supplement: S1 Data — (ZIP) [file pone.0301439.s001.zip › test_a/data/31_rain.png]

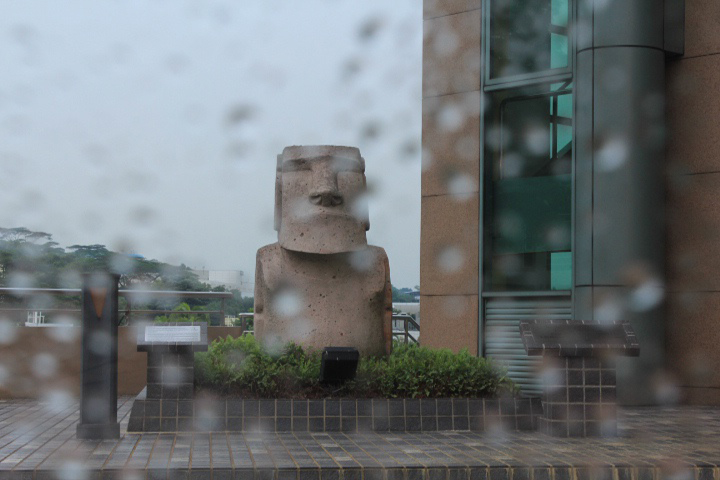

Supplement: S1 Data — (ZIP) [file pone.0301439.s001.zip › test_a/data/32_rain.png]

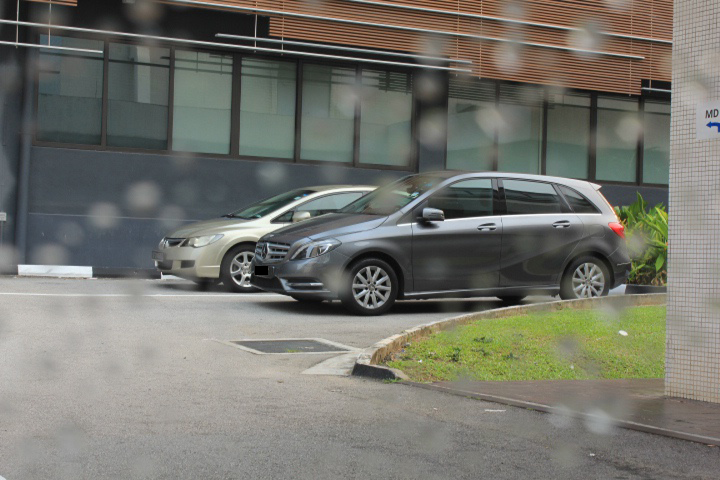

Supplement: S1 Data — (ZIP) [file pone.0301439.s001.zip › test_a/data/33_rain.png]

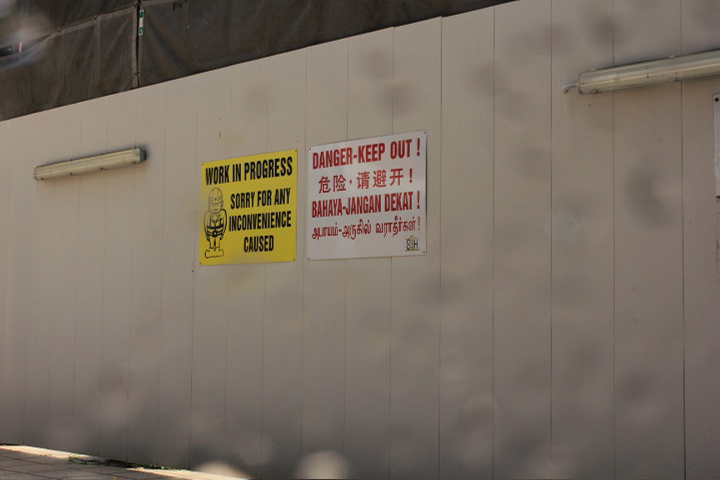

Supplement: S1 Data — (ZIP) [file pone.0301439.s001.zip › test_a/data/34_rain.png]

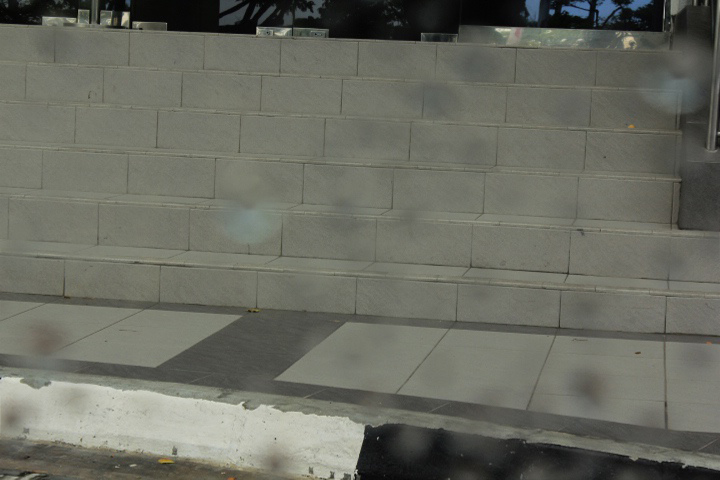

Supplement: S1 Data — (ZIP) [file pone.0301439.s001.zip › test_a/data/35_rain.png]

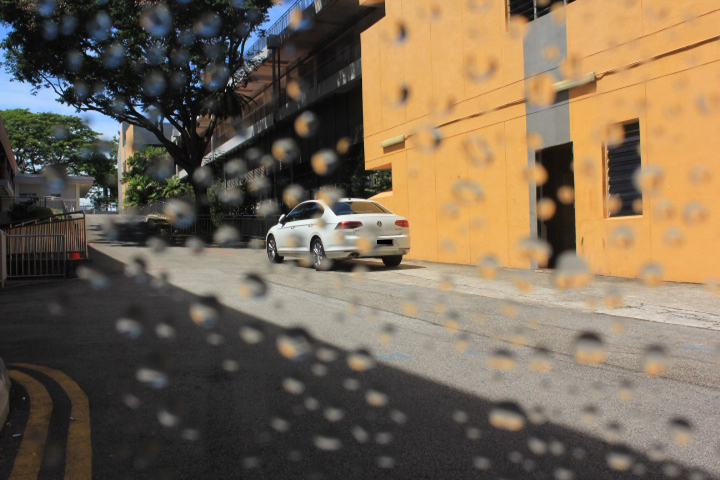

Supplement: S1 Data — (ZIP) [file pone.0301439.s001.zip › test_a/data/36_rain.png]

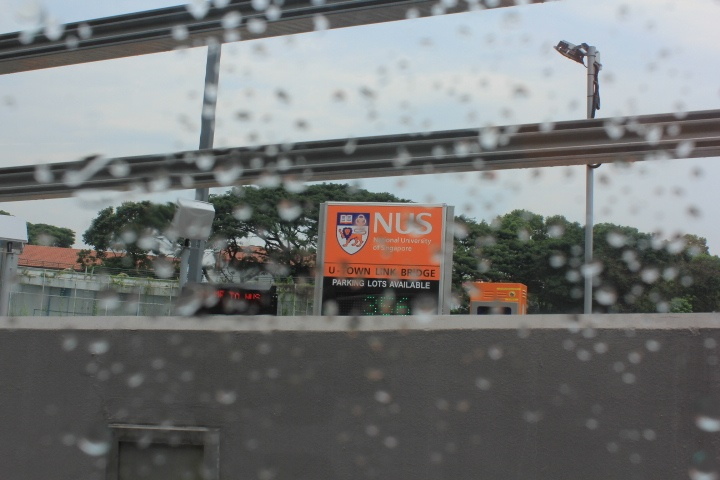

Supplement: S1 Data — (ZIP) [file pone.0301439.s001.zip › test_a/data/37_rain.png]

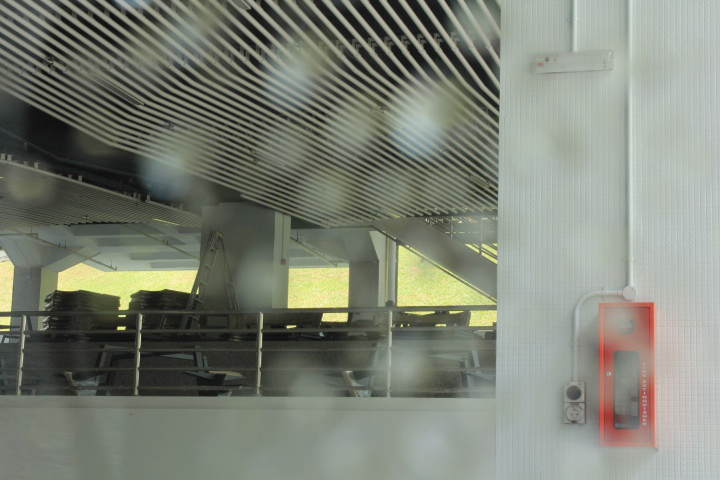

Supplement: S1 Data — (ZIP) [file pone.0301439.s001.zip › test_a/data/38_rain.png]

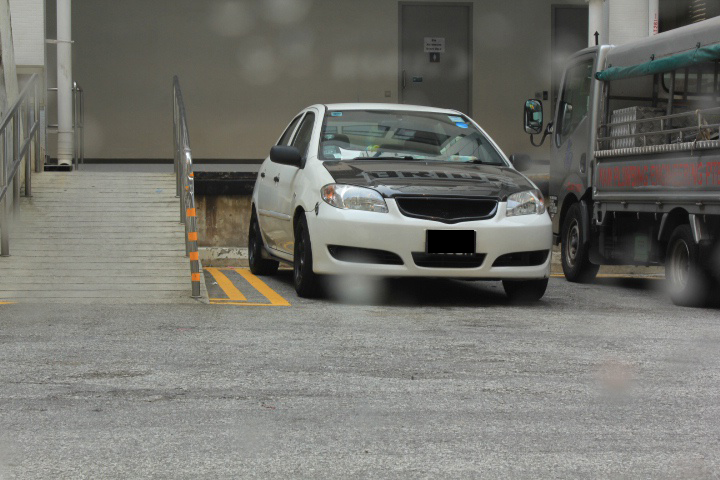

Supplement: S1 Data — (ZIP) [file pone.0301439.s001.zip › test_a/data/39_rain.png]

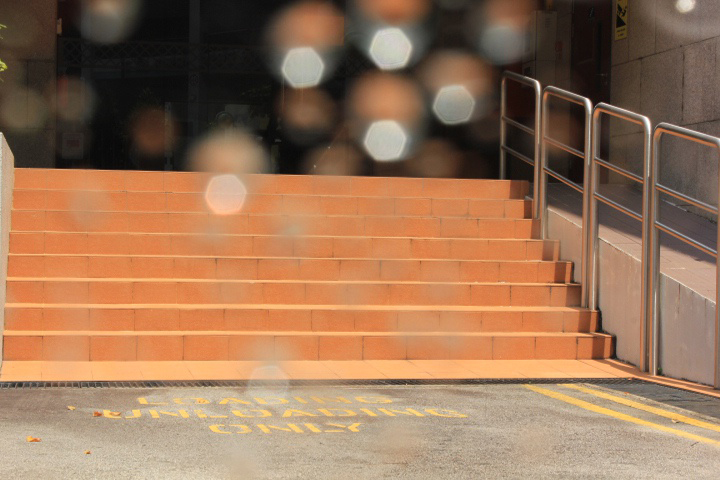

Supplement: S1 Data — (ZIP) [file pone.0301439.s001.zip › test_a/data/3_rain.png]

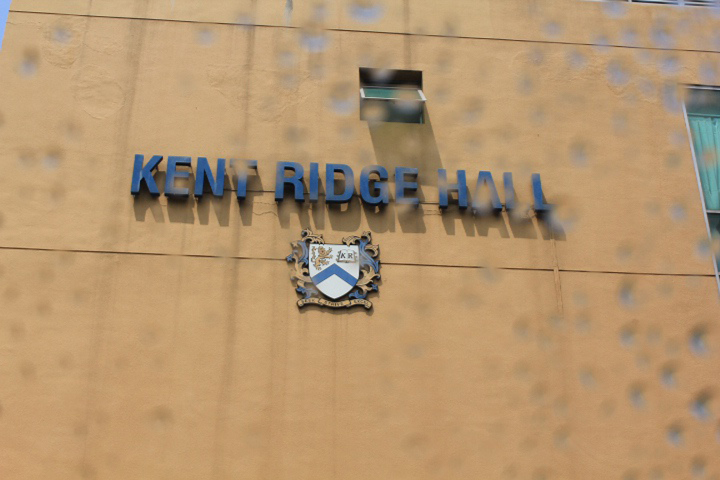

Supplement: S1 Data — (ZIP) [file pone.0301439.s001.zip › test_a/data/40_rain.png]

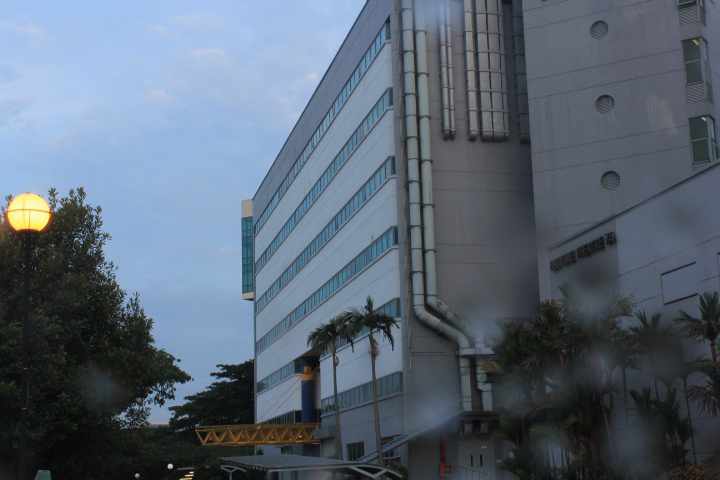

Supplement: S1 Data — (ZIP) [file pone.0301439.s001.zip › test_a/data/41_rain.png]

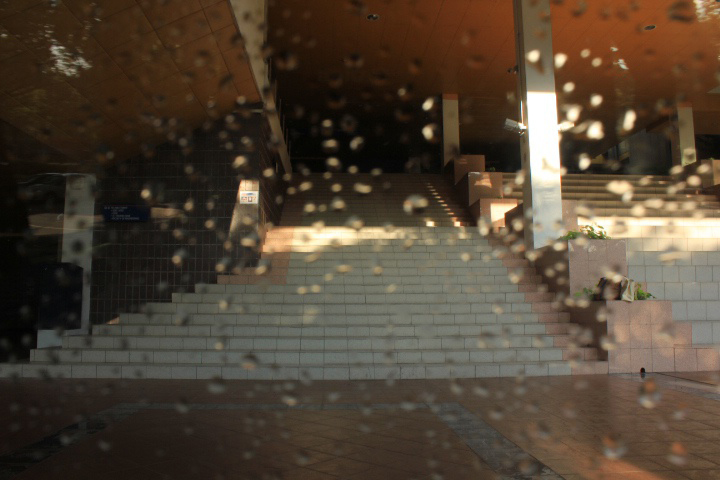

Supplement: S1 Data — (ZIP) [file pone.0301439.s001.zip › test_a/data/42_rain.png]

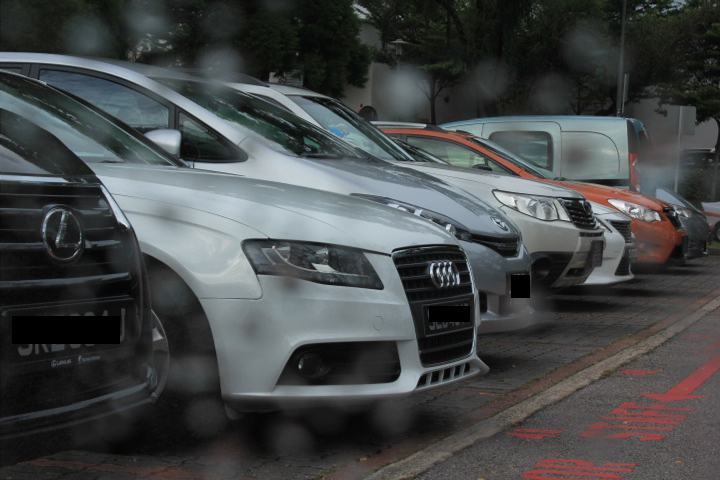

Supplement: S1 Data — (ZIP) [file pone.0301439.s001.zip › test_a/data/43_rain.png]

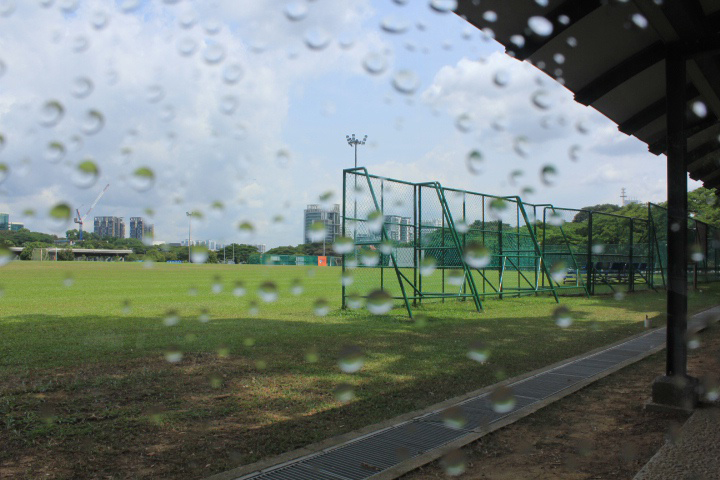

Supplement: S1 Data — (ZIP) [file pone.0301439.s001.zip › test_a/data/44_rain.png]

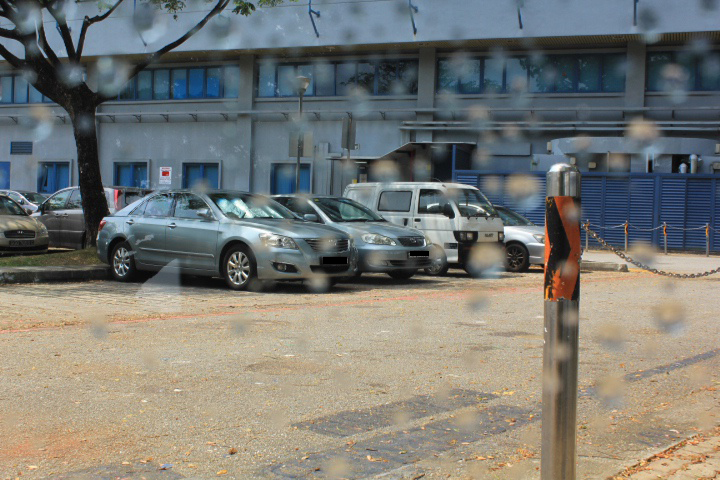

Supplement: S1 Data — (ZIP) [file pone.0301439.s001.zip › test_a/data/45_rain.png]

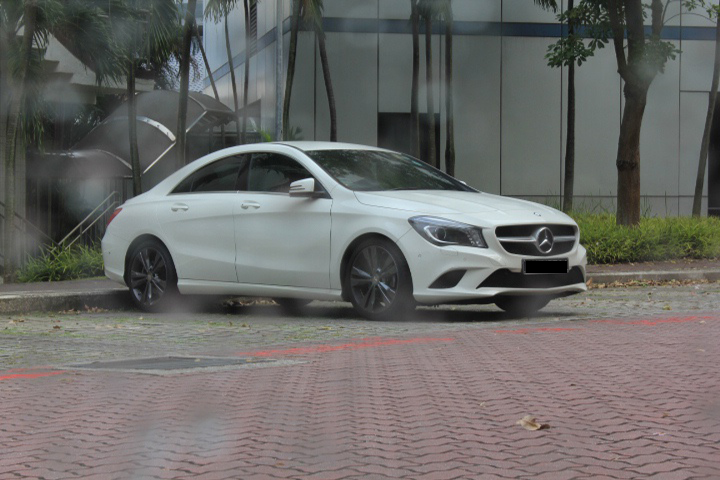

Supplement: S1 Data — (ZIP) [file pone.0301439.s001.zip › test_a/data/46_rain.png]

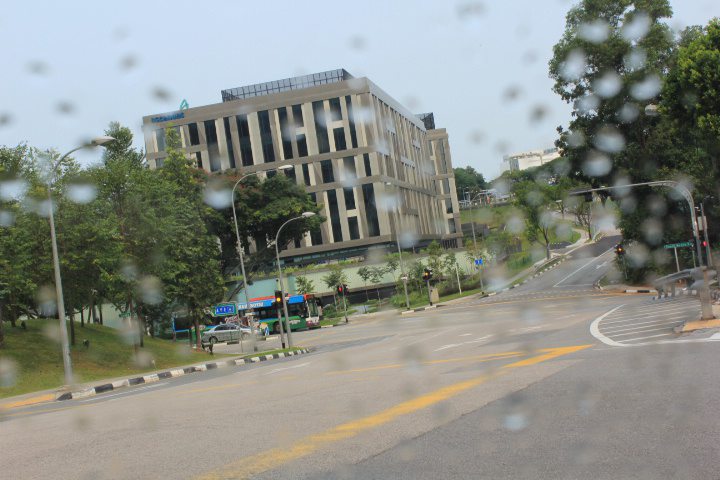

Supplement: S1 Data — (ZIP) [file pone.0301439.s001.zip › test_a/data/47_rain.png]

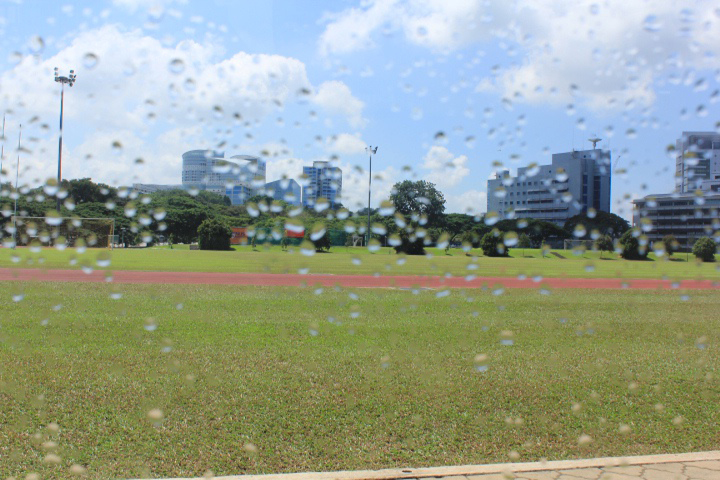

Supplement: S1 Data — (ZIP) [file pone.0301439.s001.zip › test_a/data/48_rain.png]

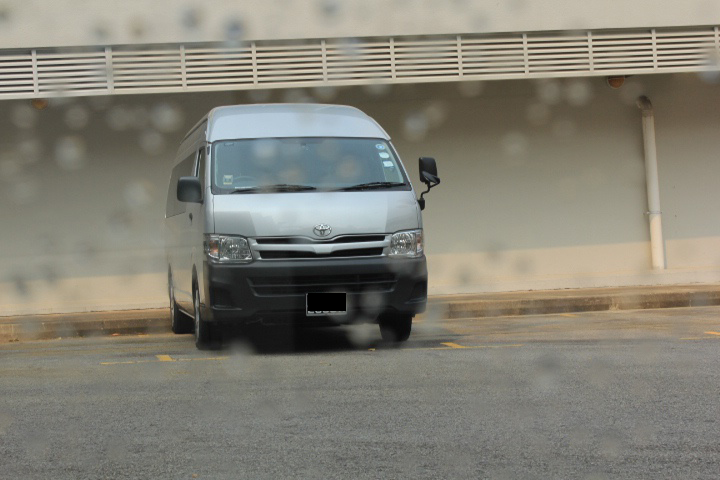

Supplement: S1 Data — (ZIP) [file pone.0301439.s001.zip › test_a/data/49_rain.png]

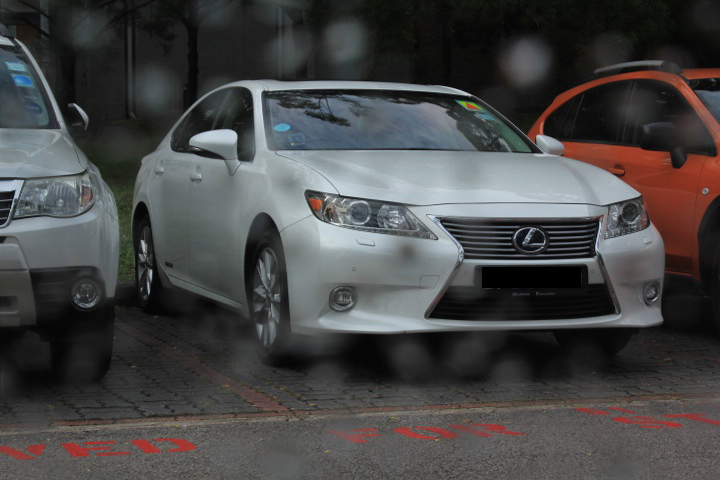

Supplement: S1 Data — (ZIP) [file pone.0301439.s001.zip › test_a/data/4_rain.png]

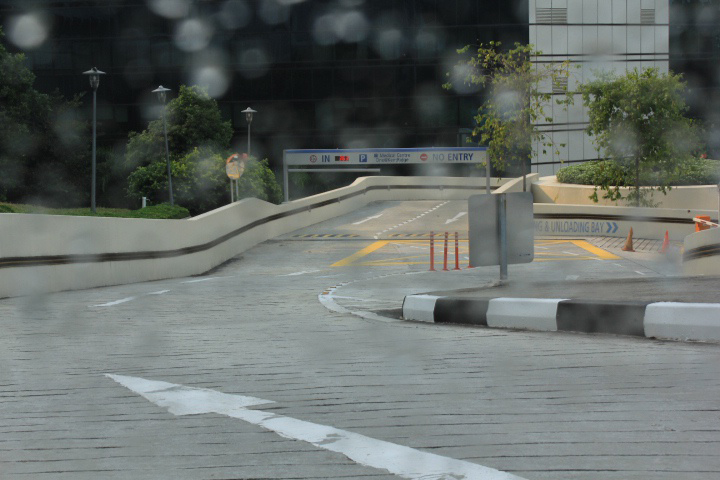

Supplement: S1 Data — (ZIP) [file pone.0301439.s001.zip › test_a/data/50_rain.png]

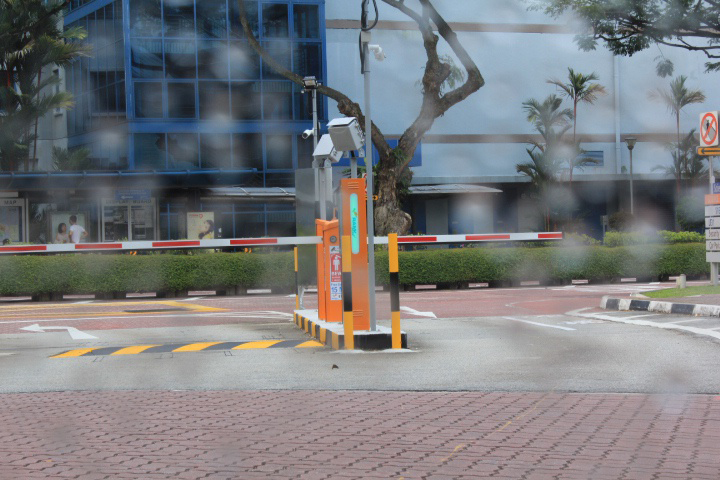

Supplement: S1 Data — (ZIP) [file pone.0301439.s001.zip › test_a/data/51_rain.png]

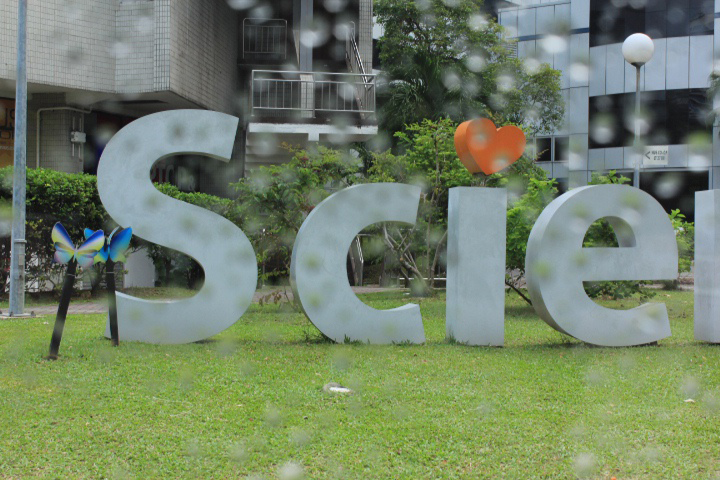

Supplement: S1 Data — (ZIP) [file pone.0301439.s001.zip › test_a/data/52_rain.png]

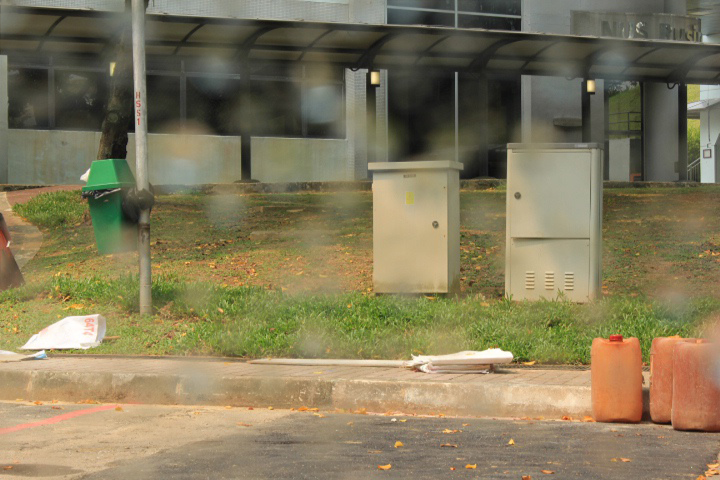

Supplement: S1 Data — (ZIP) [file pone.0301439.s001.zip › test_a/data/53_rain.png]

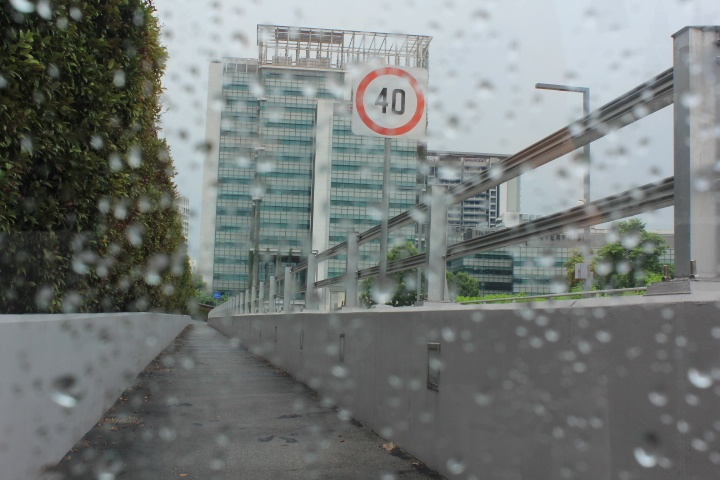

Supplement: S1 Data — (ZIP) [file pone.0301439.s001.zip › test_a/data/54_rain.png]

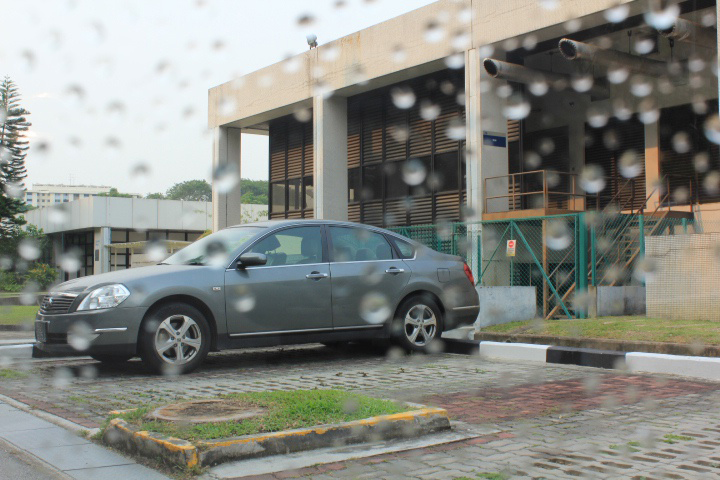

Supplement: S1 Data — (ZIP) [file pone.0301439.s001.zip › test_a/data/55_rain.png]

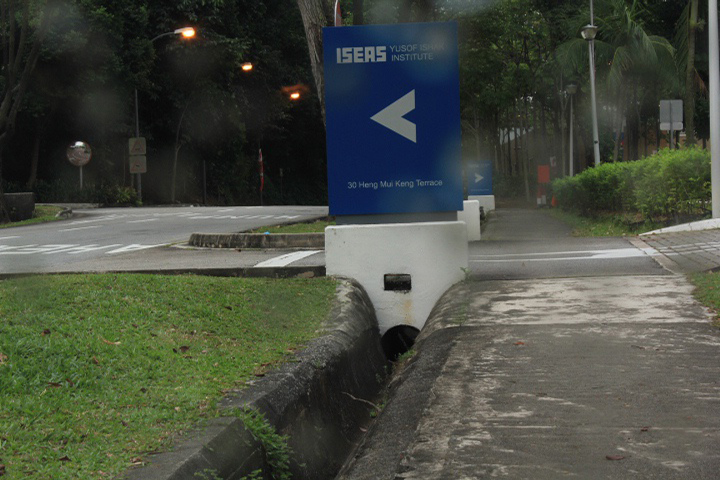

Supplement: S1 Data — (ZIP) [file pone.0301439.s001.zip › test_a/data/56_rain.png]

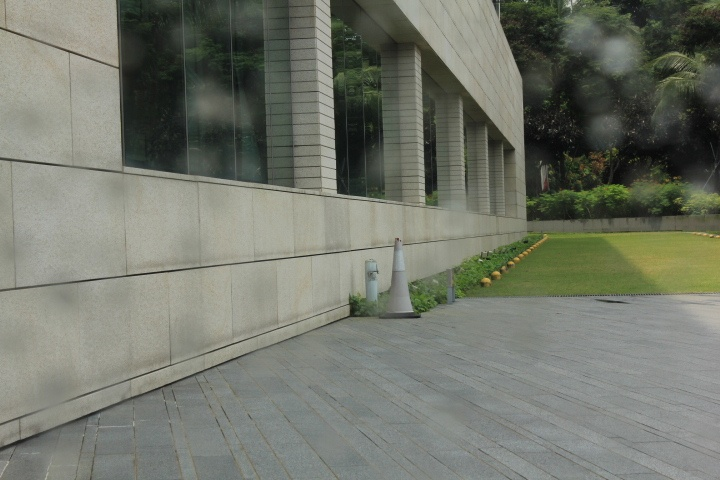

Supplement: S1 Data — (ZIP) [file pone.0301439.s001.zip › test_a/data/57_rain.png]

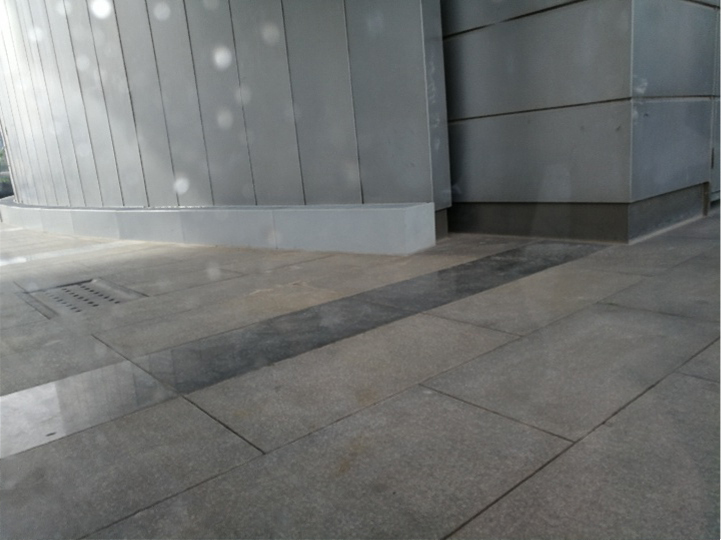

Supplement: S1 Data — (ZIP) [file pone.0301439.s001.zip › test_a/data/5_rain.png]

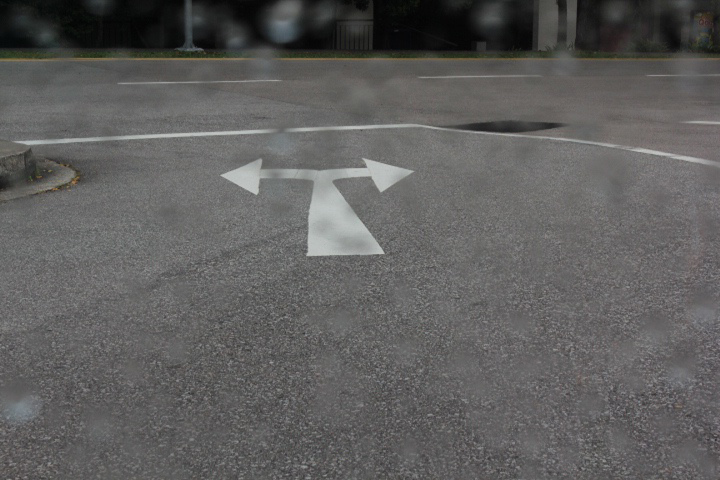

Supplement: S1 Data — (ZIP) [file pone.0301439.s001.zip › test_a/data/6_rain.png]

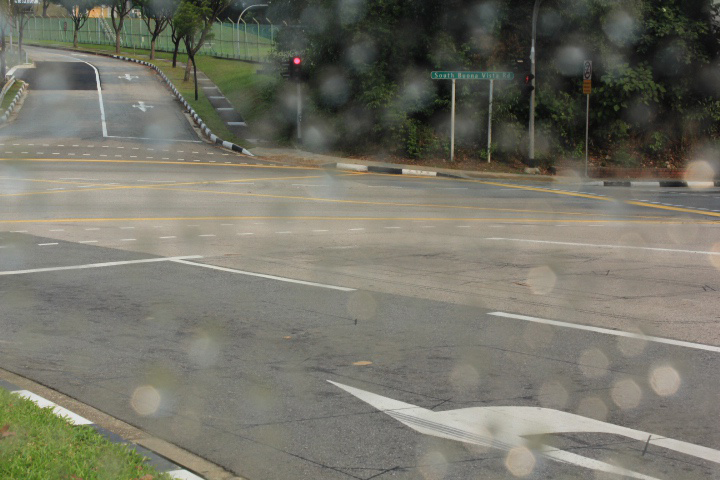

Supplement: S1 Data — (ZIP) [file pone.0301439.s001.zip › test_a/data/7_rain.png]

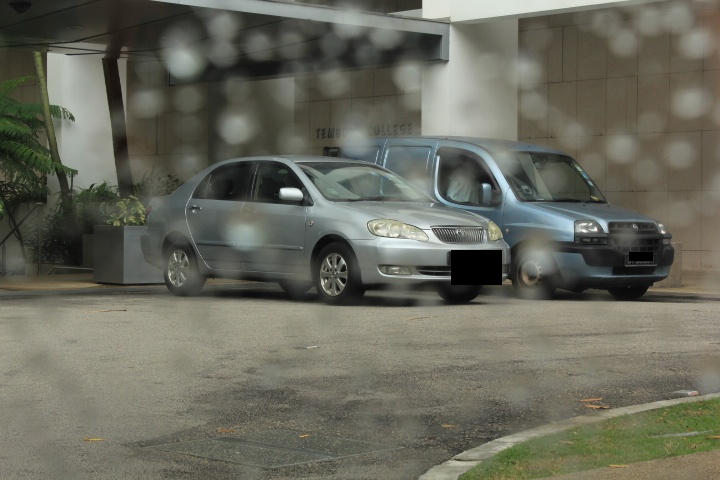

Supplement: S1 Data — (ZIP) [file pone.0301439.s001.zip › test_a/data/8_rain.png]

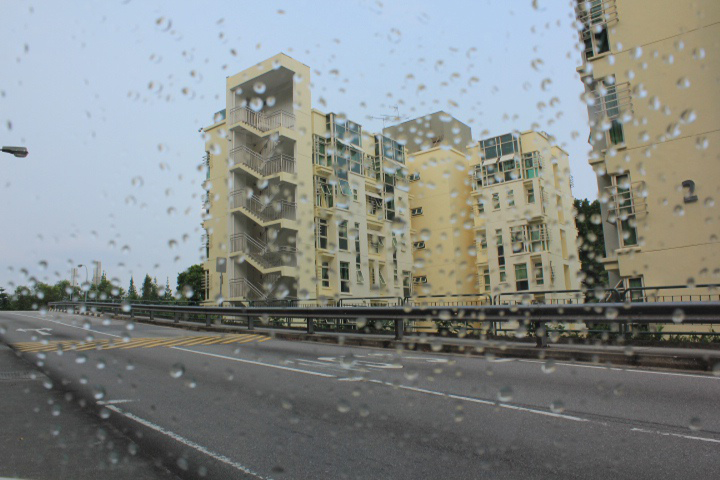

Supplement: S1 Data — (ZIP) [file pone.0301439.s001.zip › test_a/data/9_rain.png]

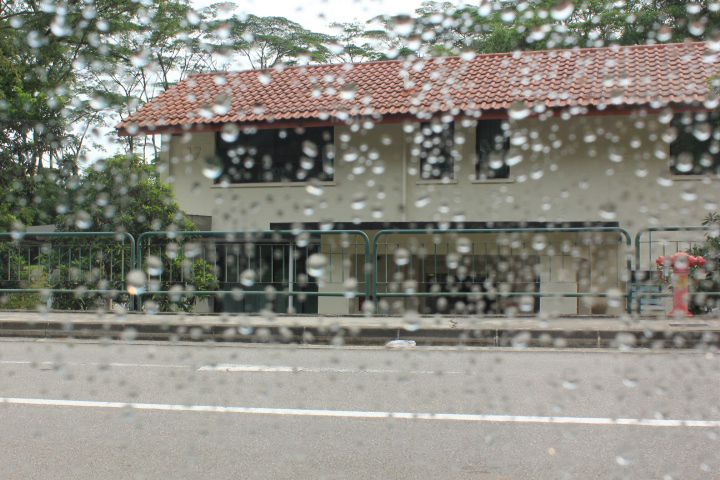

Supplement: S1 Data — (ZIP) [file pone.0301439.s001.zip › test_b/data/0_rain.jpg]

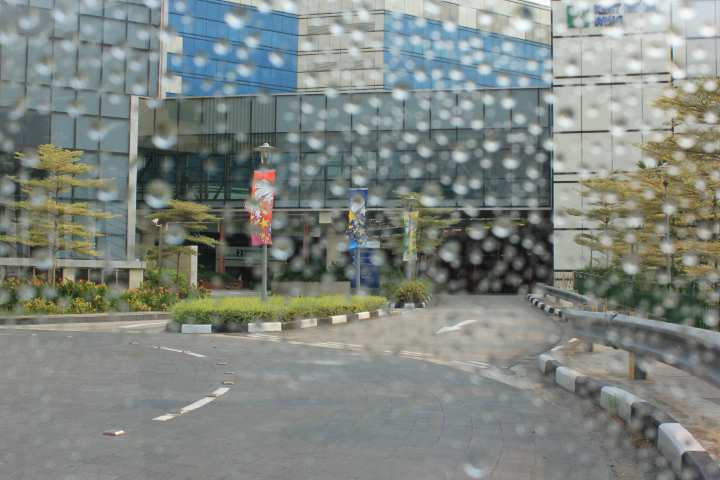

Supplement: S1 Data — (ZIP) [file pone.0301439.s001.zip › test_b/data/100_rain.jpg]

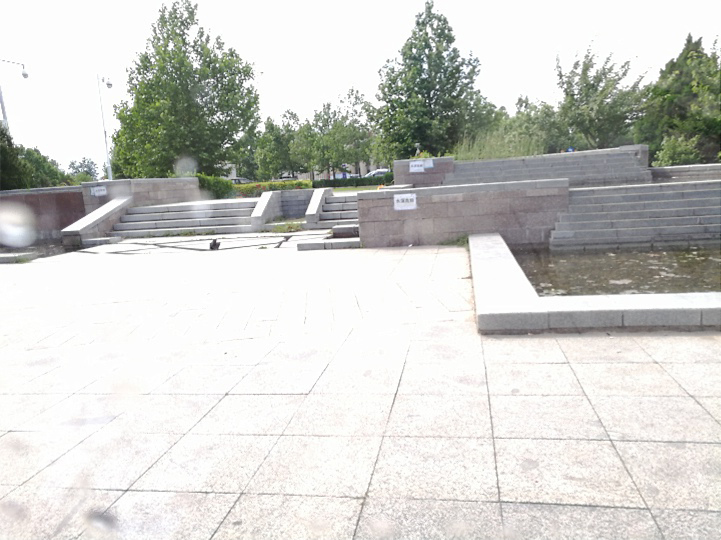

Supplement: S1 Data — (ZIP) [file pone.0301439.s001.zip › test_b/data/101_rain.jpg]

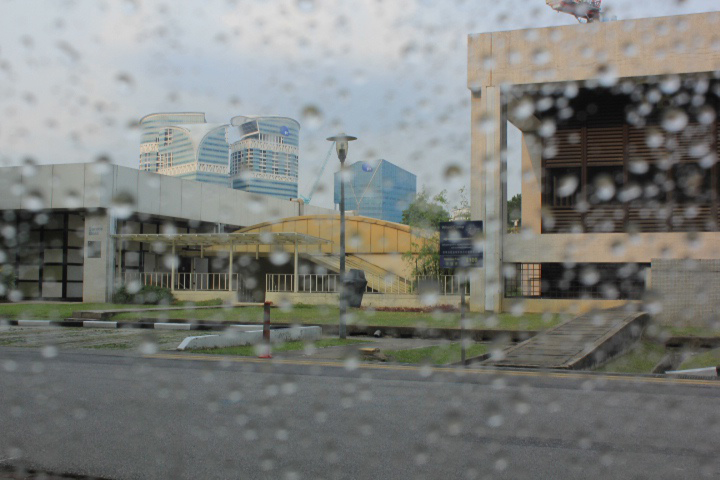

Supplement: S1 Data — (ZIP) [file pone.0301439.s001.zip › test_b/data/102_rain.jpg]

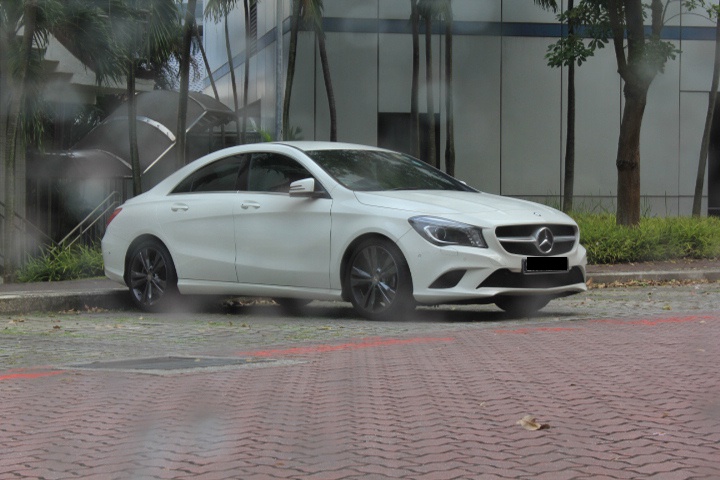

Supplement: S1 Data — (ZIP) [file pone.0301439.s001.zip › test_b/data/103_rain.jpg]

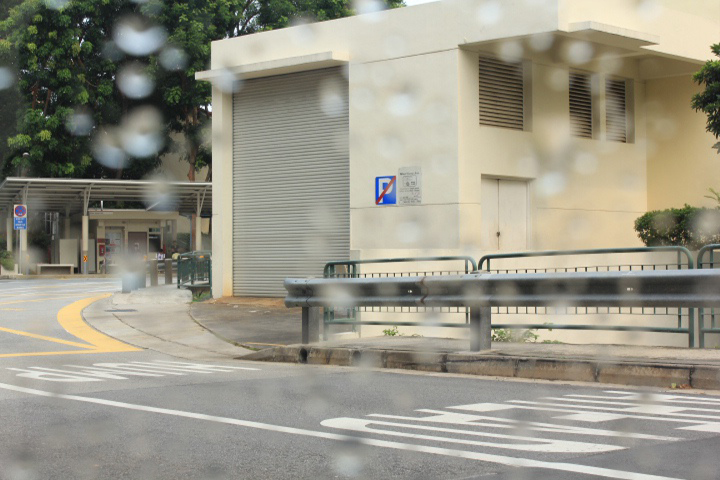

Supplement: S1 Data — (ZIP) [file pone.0301439.s001.zip › test_b/data/104_rain.jpg]

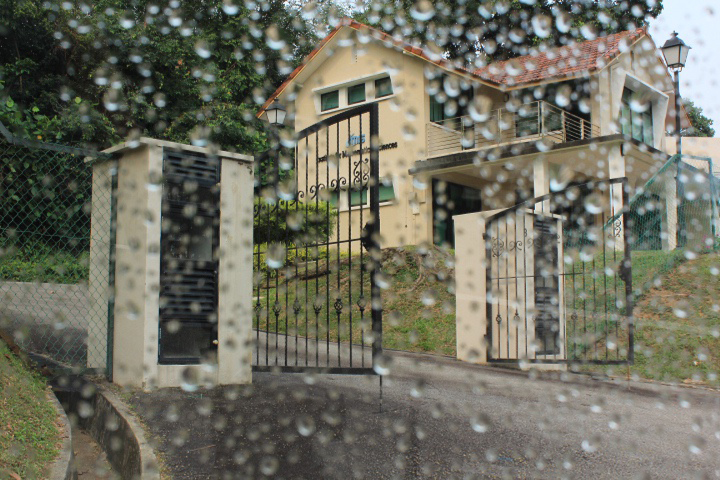

Supplement: S1 Data — (ZIP) [file pone.0301439.s001.zip › test_b/data/105_rain.jpg]

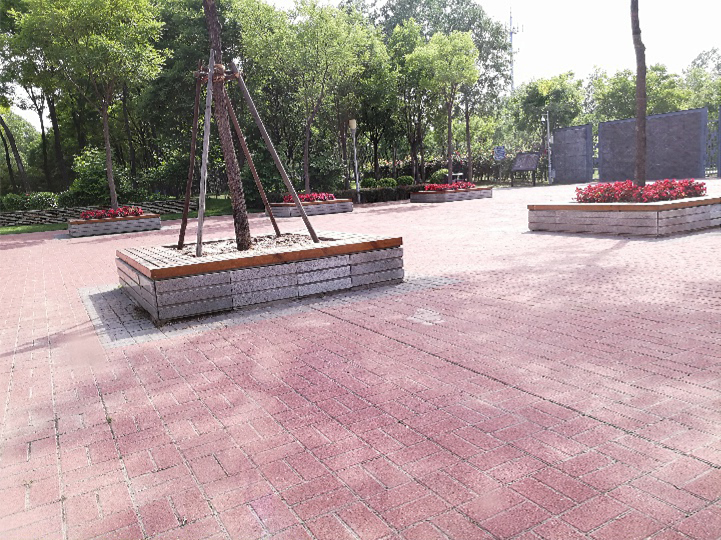

Supplement: S1 Data — (ZIP) [file pone.0301439.s001.zip › test_b/data/106_rain.jpg]

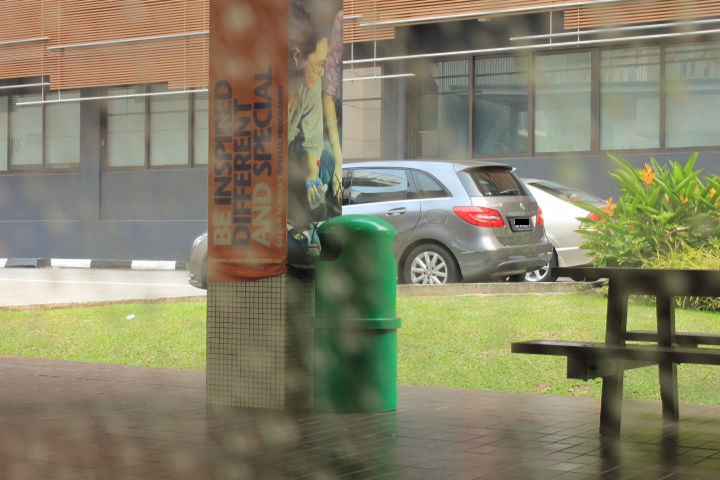

Supplement: S1 Data — (ZIP) [file pone.0301439.s001.zip › test_b/data/107_rain.jpg]

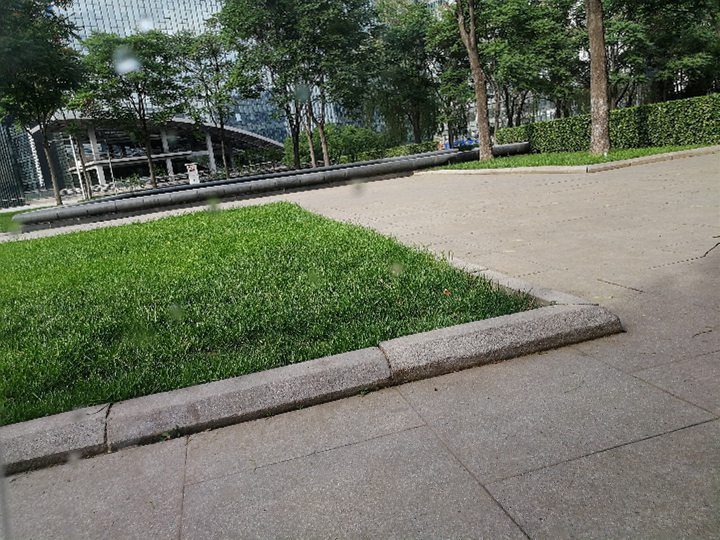

Supplement: S1 Data — (ZIP) [file pone.0301439.s001.zip › test_b/data/108_rain.jpg]

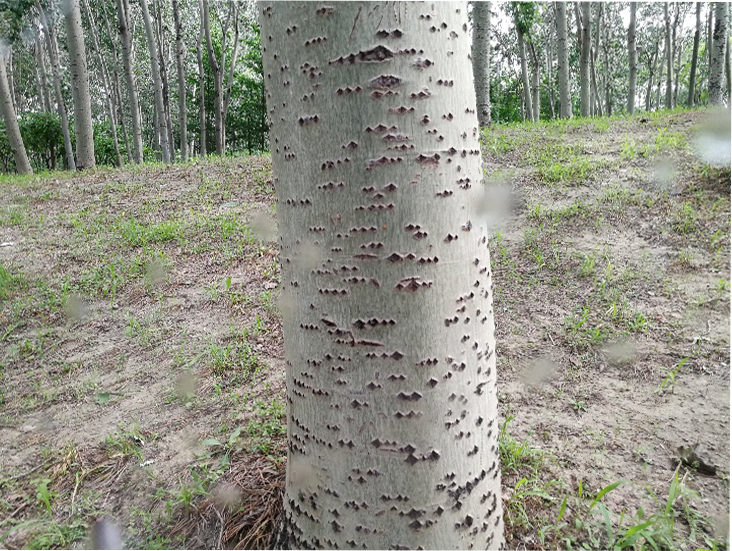

Supplement: S1 Data — (ZIP) [file pone.0301439.s001.zip › test_b/data/109_rain.jpg]

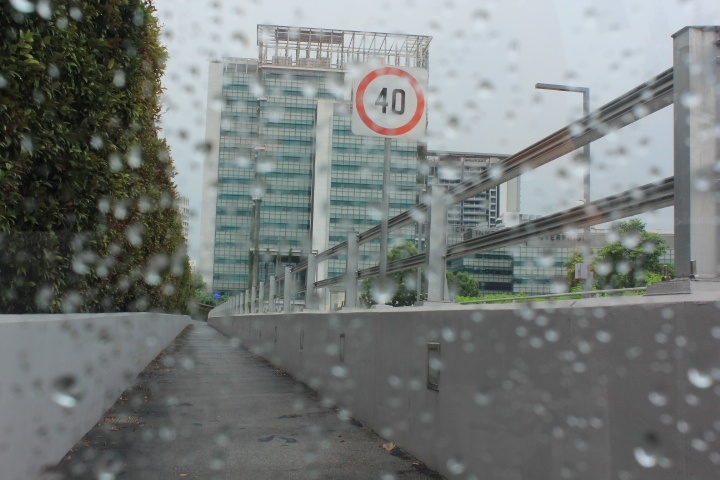

Supplement: S1 Data — (ZIP) [file pone.0301439.s001.zip › test_b/data/10_rain.jpg]

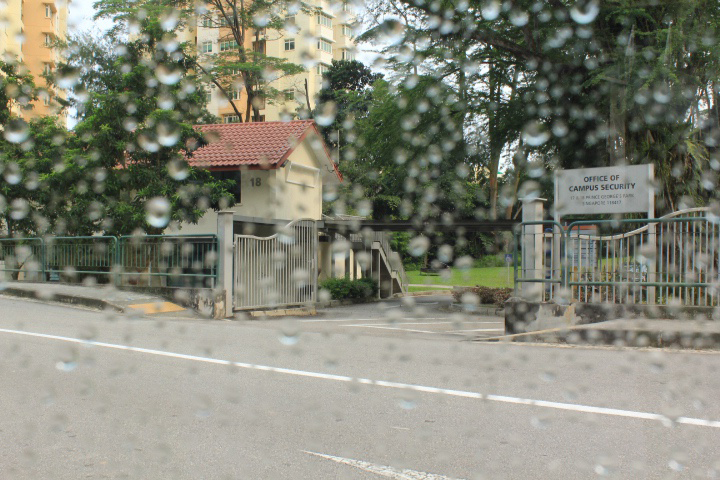

Supplement: S1 Data — (ZIP) [file pone.0301439.s001.zip › test_b/data/110_rain.jpg]

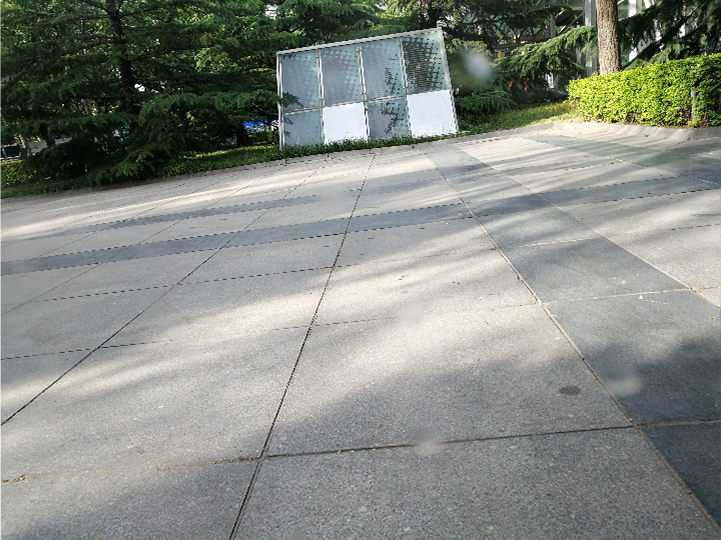

Supplement: S1 Data — (ZIP) [file pone.0301439.s001.zip › test_b/data/111_rain.jpg]

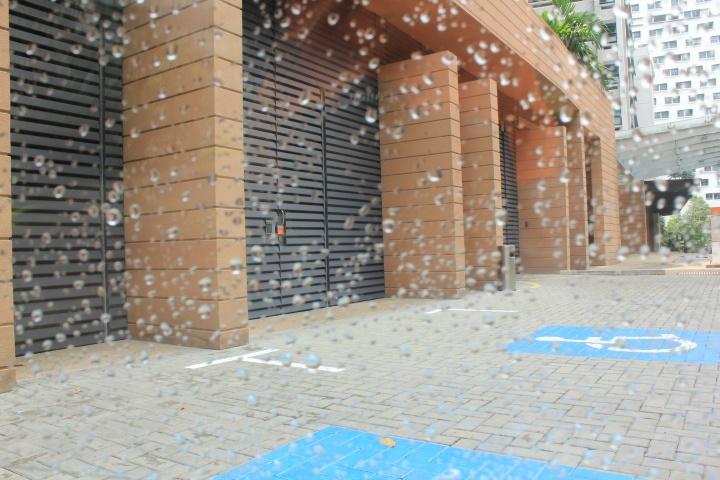

Supplement: S1 Data — (ZIP) [file pone.0301439.s001.zip › test_b/data/112_rain.jpg]

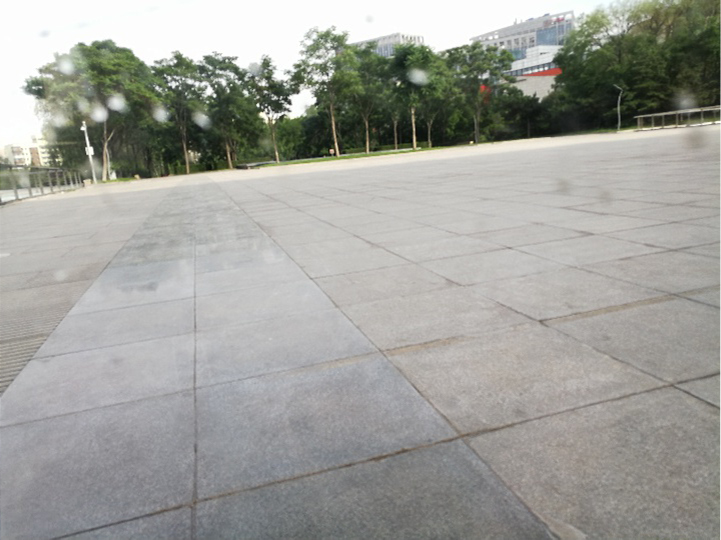

Supplement: S1 Data — (ZIP) [file pone.0301439.s001.zip › test_b/data/113_rain.jpg]

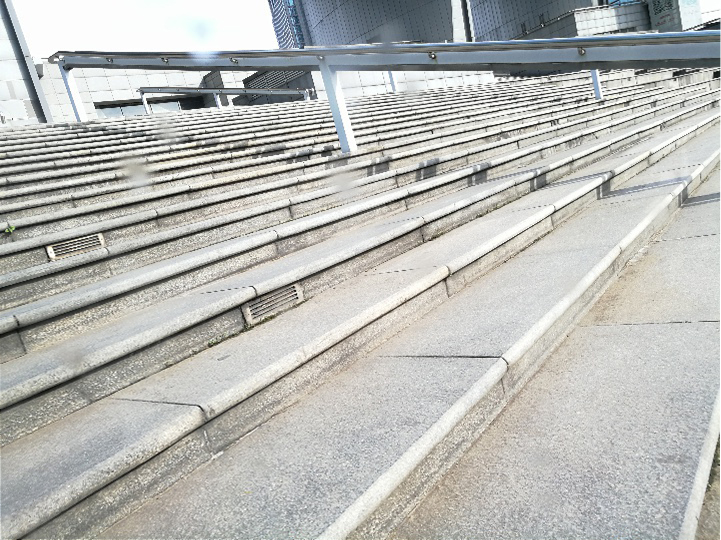

Supplement: S1 Data — (ZIP) [file pone.0301439.s001.zip › test_b/data/114_rain.jpg]

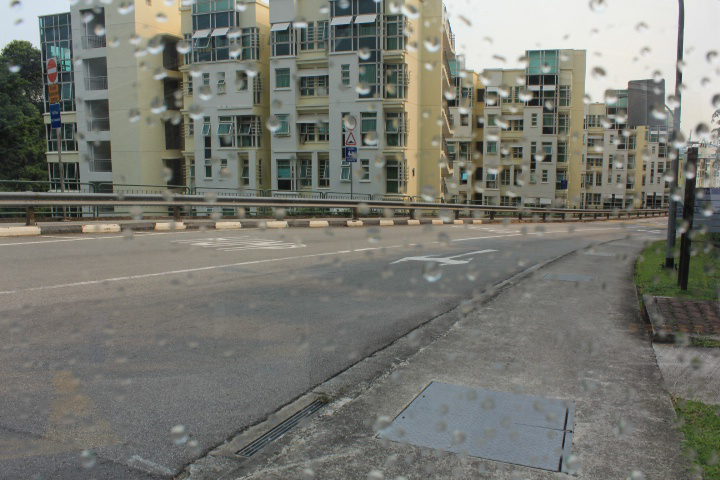

Supplement: S1 Data — (ZIP) [file pone.0301439.s001.zip › test_b/data/115_rain.jpg]

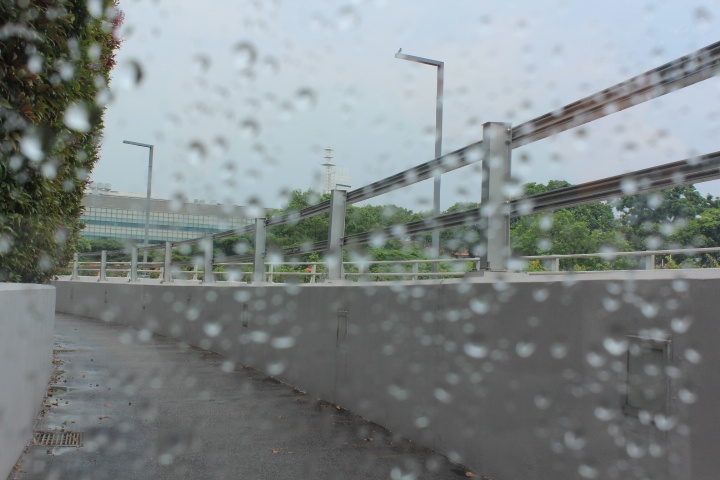

Supplement: S1 Data — (ZIP) [file pone.0301439.s001.zip › test_b/data/116_rain.jpg]

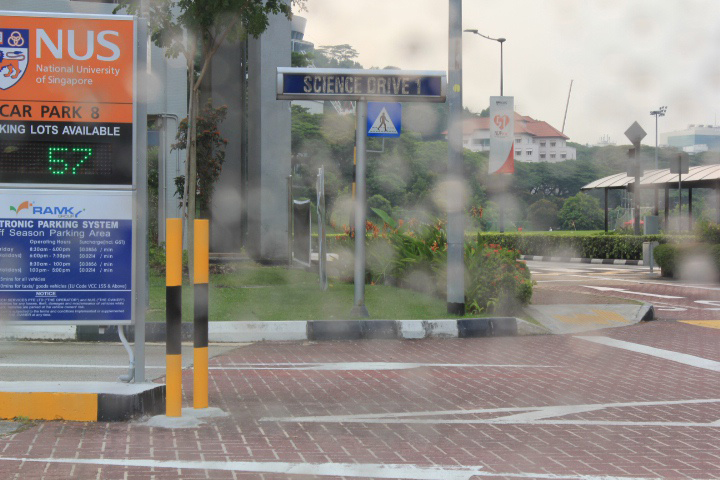

Supplement: S1 Data — (ZIP) [file pone.0301439.s001.zip › test_b/data/117_rain.jpg]

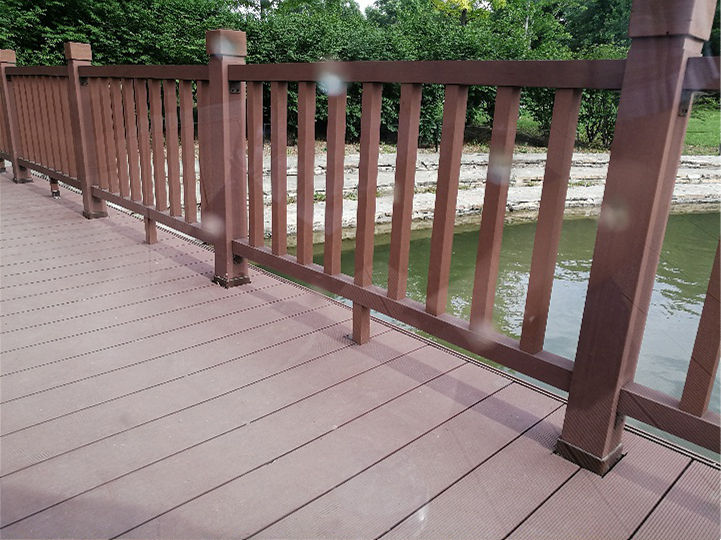

Supplement: S1 Data — (ZIP) [file pone.0301439.s001.zip › test_b/data/118_rain.jpg]

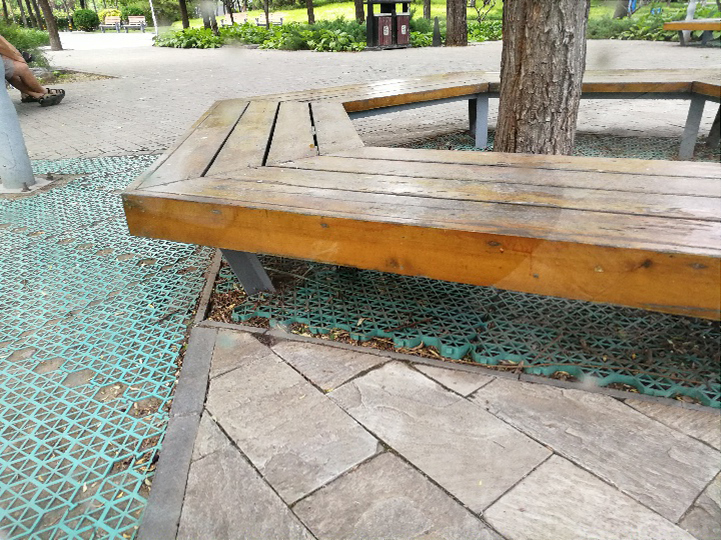

Supplement: S1 Data — (ZIP) [file pone.0301439.s001.zip › test_b/data/119_rain.jpg]

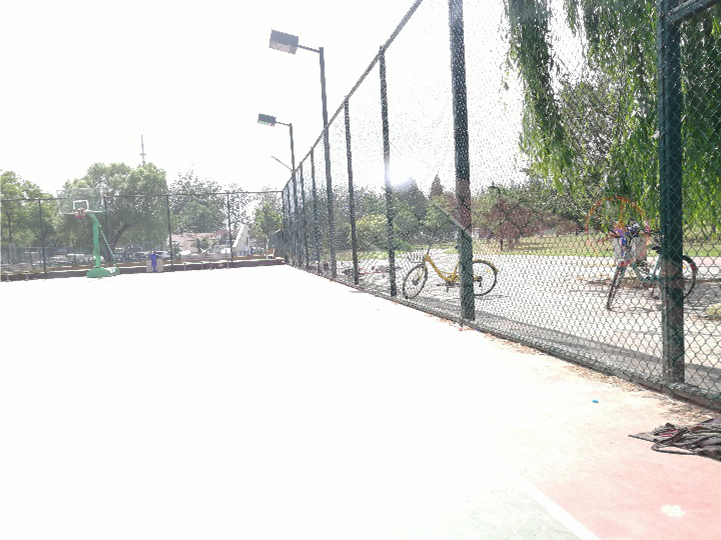

Supplement: S1 Data — (ZIP) [file pone.0301439.s001.zip › test_b/data/11_rain.jpg]

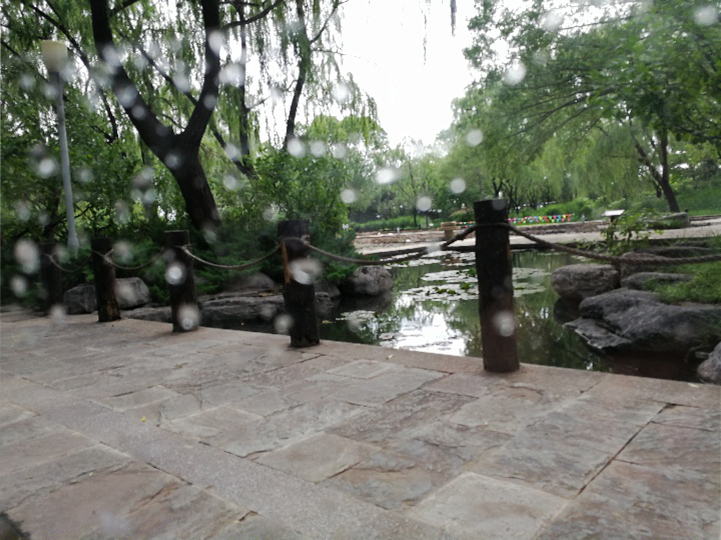

Supplement: S1 Data — (ZIP) [file pone.0301439.s001.zip › test_b/data/120_rain.jpg]

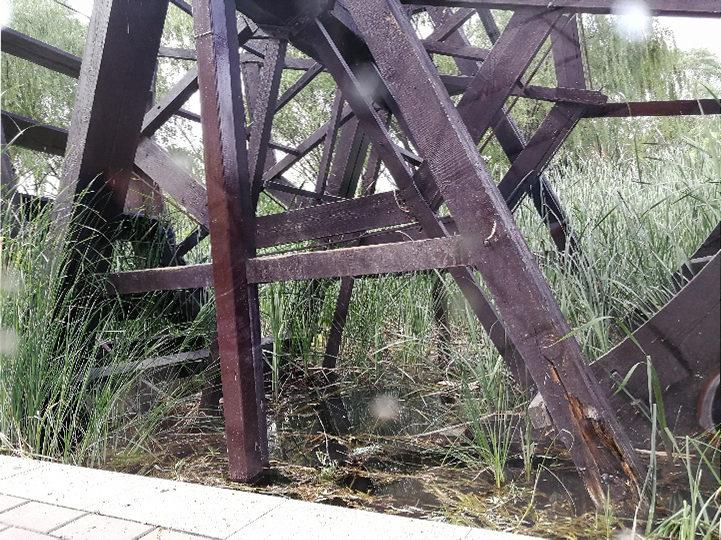

Supplement: S1 Data — (ZIP) [file pone.0301439.s001.zip › test_b/data/121_rain.jpg]

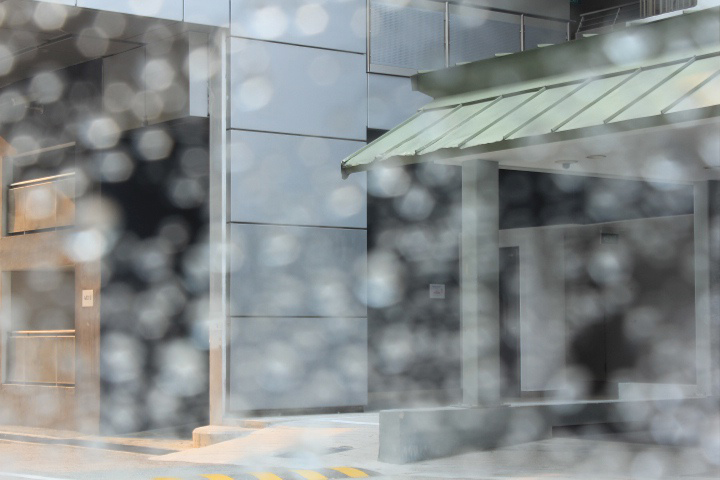

Supplement: S1 Data — (ZIP) [file pone.0301439.s001.zip › test_b/data/122_rain.jpg]

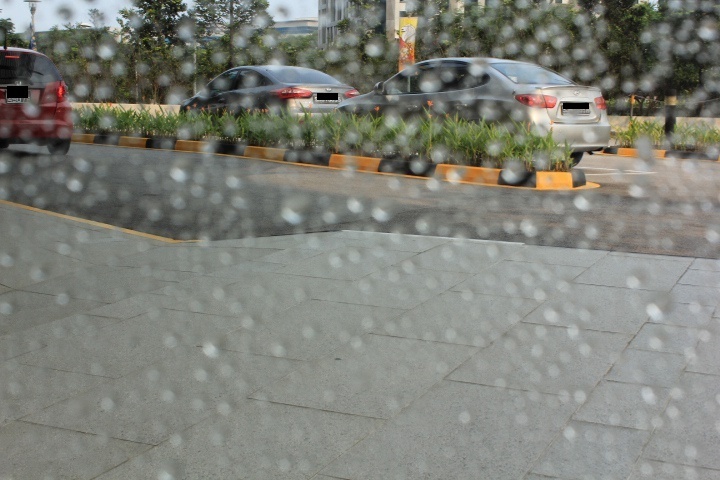

Supplement: S1 Data — (ZIP) [file pone.0301439.s001.zip › test_b/data/123_rain.jpg]

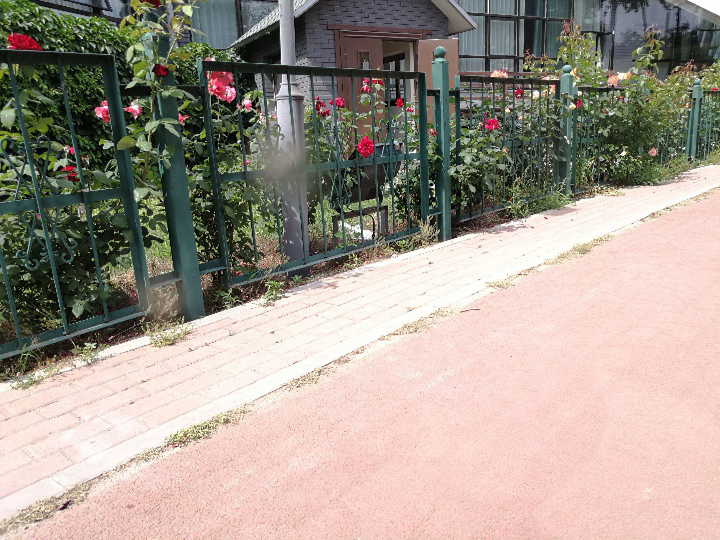

Supplement: S1 Data — (ZIP) [file pone.0301439.s001.zip › test_b/data/124_rain.jpg]

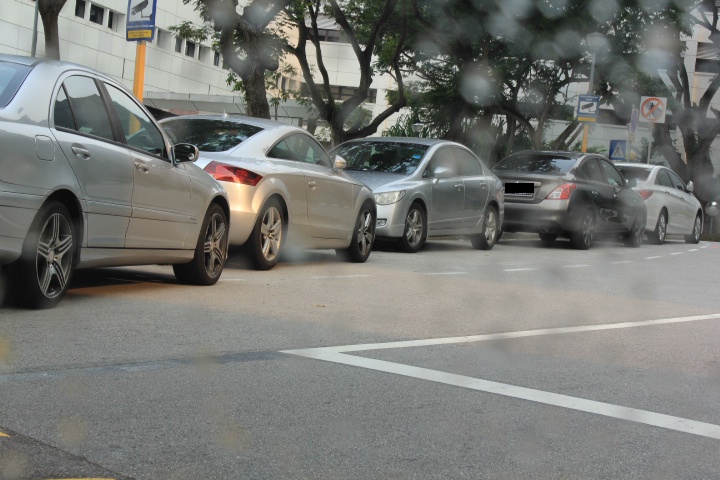

Supplement: S1 Data — (ZIP) [file pone.0301439.s001.zip › test_b/data/125_rain.jpg]

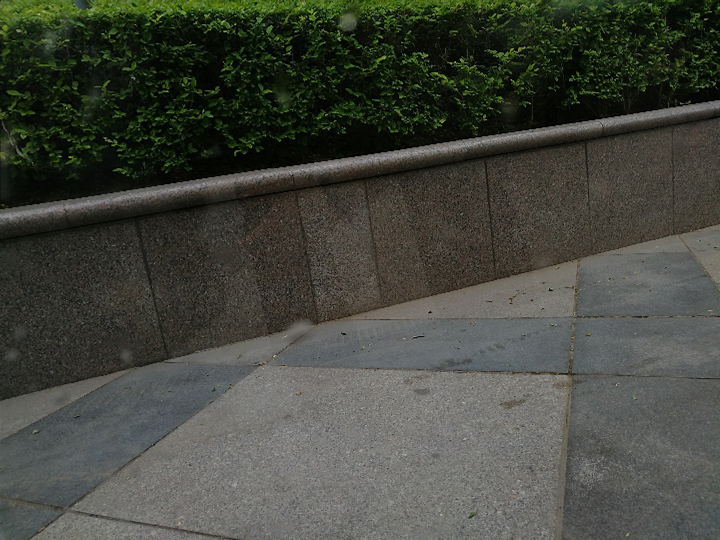

Supplement: S1 Data — (ZIP) [file pone.0301439.s001.zip › test_b/data/126_rain.jpg]

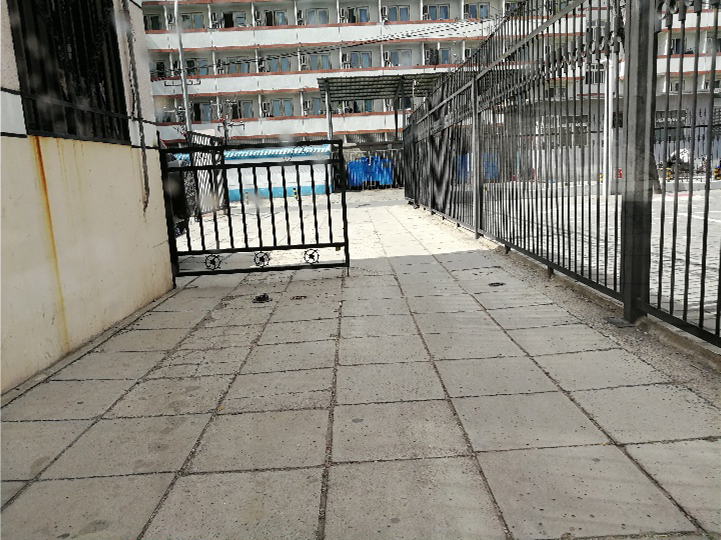

Supplement: S1 Data — (ZIP) [file pone.0301439.s001.zip › test_b/data/127_rain.jpg]

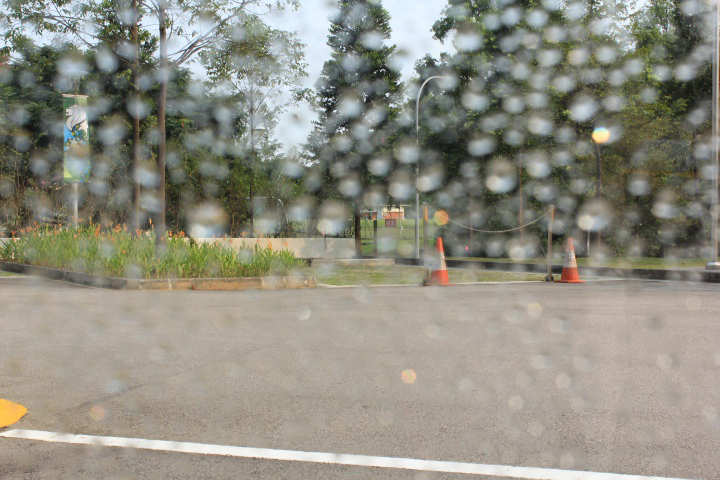

Supplement: S1 Data — (ZIP) [file pone.0301439.s001.zip › test_b/data/128_rain.jpg]

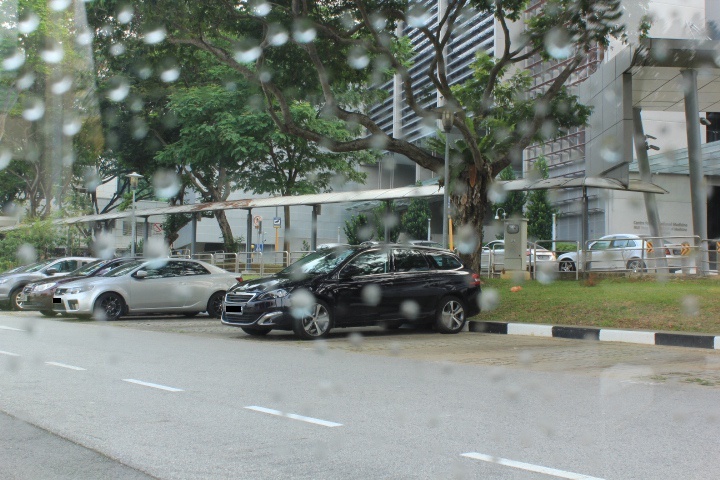

Supplement: S1 Data — (ZIP) [file pone.0301439.s001.zip › test_b/data/129_rain.jpg]

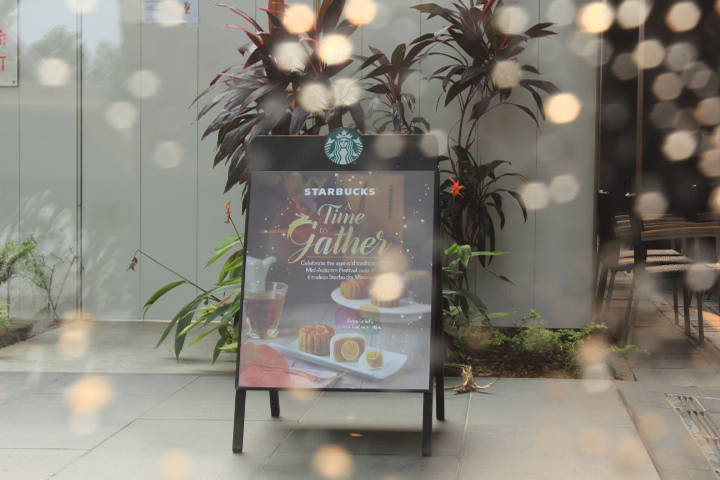

Supplement: S1 Data — (ZIP) [file pone.0301439.s001.zip › test_b/data/12_rain.jpg]

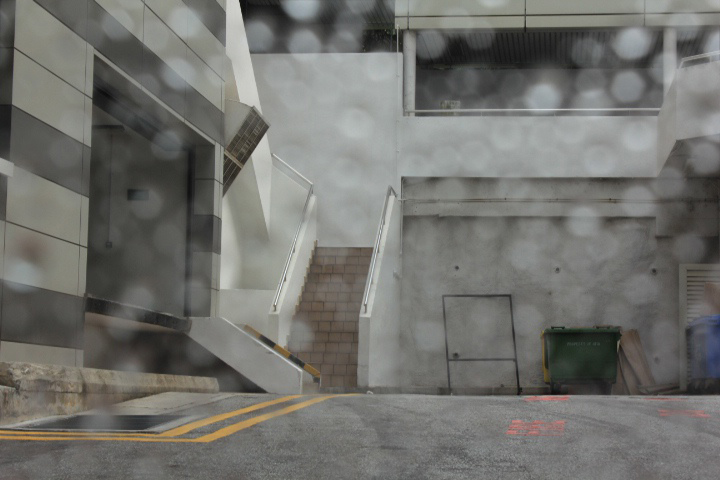

Supplement: S1 Data — (ZIP) [file pone.0301439.s001.zip › test_b/data/130_rain.jpg]

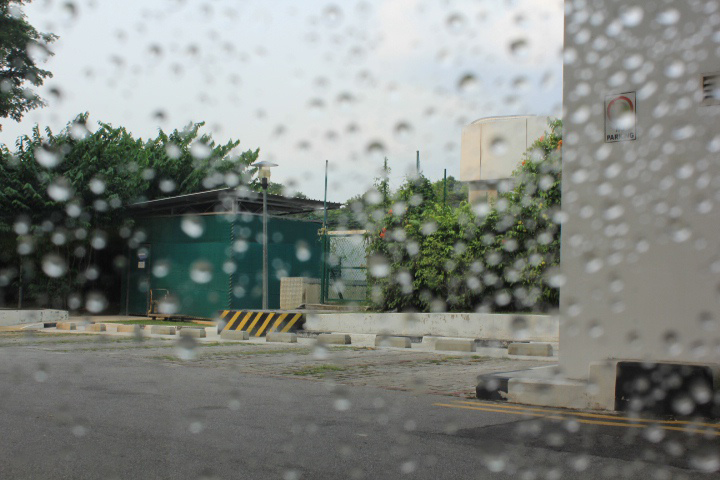

Supplement: S1 Data — (ZIP) [file pone.0301439.s001.zip › test_b/data/131_rain.jpg]

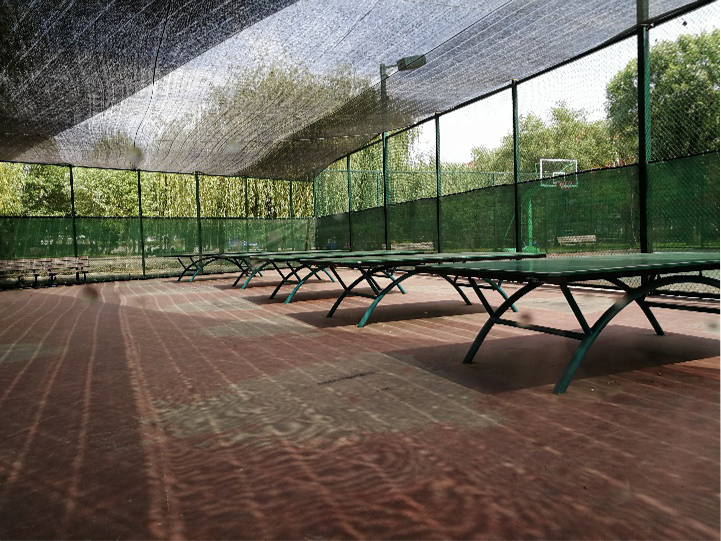

Supplement: S1 Data — (ZIP) [file pone.0301439.s001.zip › test_b/data/132_rain.jpg]

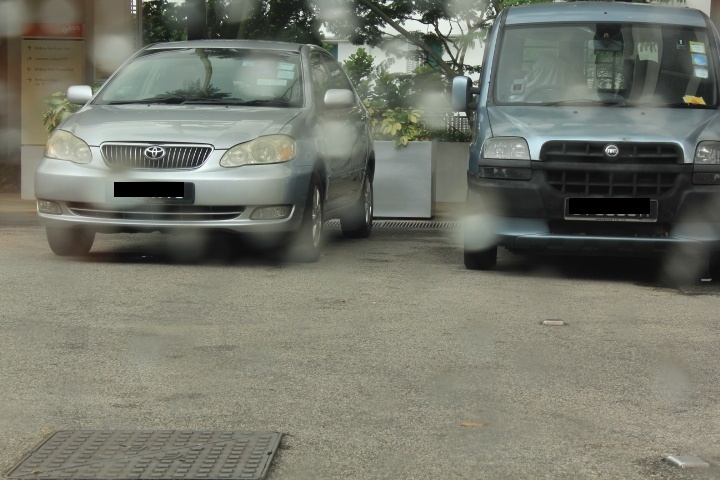

Supplement: S1 Data — (ZIP) [file pone.0301439.s001.zip › test_b/data/133_rain.jpg]

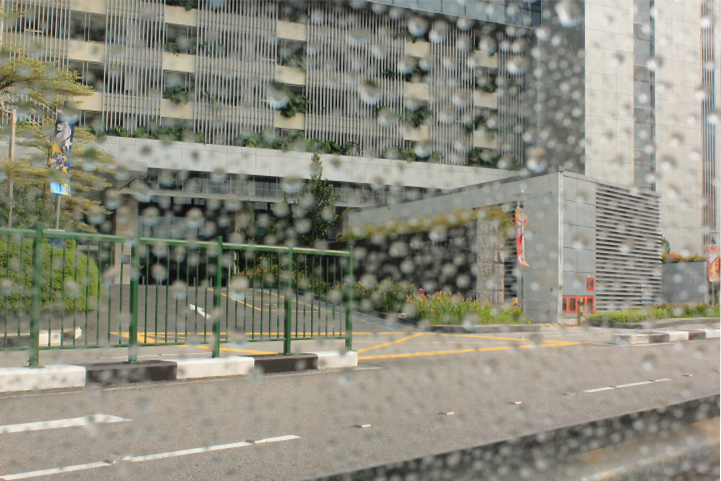

Supplement: S1 Data — (ZIP) [file pone.0301439.s001.zip › test_b/data/134_rain.jpg]

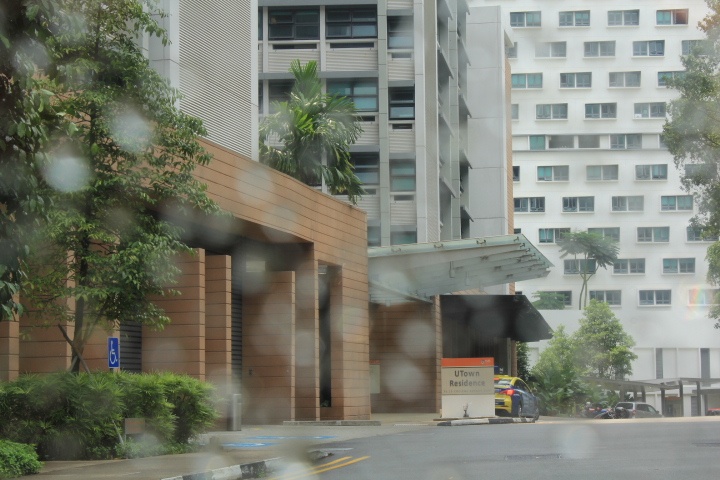

Supplement: S1 Data — (ZIP) [file pone.0301439.s001.zip › test_b/data/135_rain.jpg]

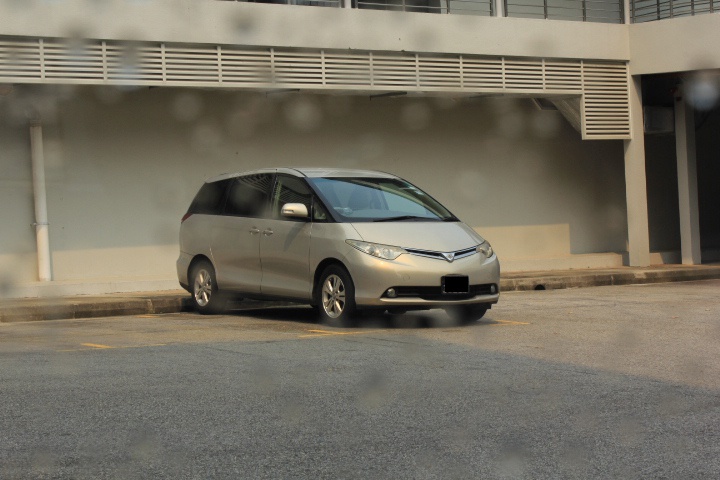

Supplement: S1 Data — (ZIP) [file pone.0301439.s001.zip › test_b/data/136_rain.jpg]

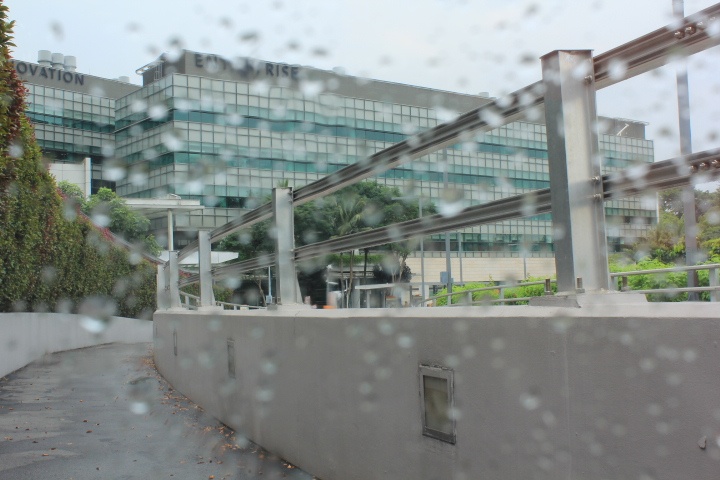

Supplement: S1 Data — (ZIP) [file pone.0301439.s001.zip › test_b/data/137_rain.jpg]
